# Reaction Mechanism of Pd‐Catalyzed “CO‐Free” Carbonylation Reaction Uncovered by In Situ Spectroscopy: The Formyl Mechanism

**DOI:** 10.1002/anie.202011152

**Published:** 2020-12-14

**Authors:** Robert Geitner, Andrei Gurinov, Tianbai Huang, Stephan Kupfer, Stefanie Gräfe, Bert M. Weckhuysen

**Affiliations:** ^1^ Inorganic Chemistry and Catalysis Group Debye Institute for Nanomaterials Science Utrecht University Universiteitsweg 99 3584 CG Utrecht The Netherlands; ^2^ NMR Spectroscopy group Bijvoet Center for Biomolecular Research Utrecht University Padualaan 8, 3584 CH Utrecht The Netherlands; ^3^ Institute for Physical Chemistry and Abbe Center of Photonics Friedrich Schiller University Jena Helmholtzweg 4 07743 Jena Germany

**Keywords:** carbonylation, in situ spectroscopy, kinetics, palladium, reaction mechanisms

## Abstract

“CO‐free” carbonylation reactions, where synthesis gas (CO/H_2_) is substituted by C1 surrogate molecules like formaldehyde or formic acid, have received widespread attention in homogeneous catalysis lately. Although a broad range of organics is available via this method, still relatively little is known about the precise reaction mechanism. In this work, we used in situ nuclear magnetic resonance (NMR) spectroscopy to unravel the mechanism of the alkoxycarbonylation of alkenes using different surrogate molecules. In contrast to previous hypotheses no carbon monoxide could be found during the reaction. Instead the reaction proceeds via the C−H activation of in situ generated methyl formate. On the basis of quantitative NMR experiments, a kinetic model involving all major intermediates is built which enables the knowledge‐driven optimization of the reaction. Finally, a new reaction mechanism is proposed on the basis of in situ observed Pd‐hydride, Pd‐formyl and Pd‐acyl species.

## Graphical abstract

Understanding reaction mechanisms is crucial to knowledge‐driven catalyst design. Here, NMR spectroscopy and DFT are combined to unravel the mechanism of “CO‐free” hydroesterifications. Methyl formate is found to be the key intermediate in this Pd‐catalyzed reaction. This is supported by the first observation of a Pd‐formyl species.

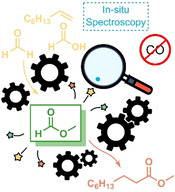

Carbonylation reactions for the production of carbonyl compounds are an important part of industrial and academic chemistry and one of the prime examples of the successful application of homogeneous catalysis.^[1]^ “CO‐free” carbonylations are a special type of reaction where the toxic CO gas is substituted by less harmful surrogate molecules, like formaldehyde or formic acid, and have received increasing attention in recent years as potential building blocks for a carbon‐neutral circular economy.[^2^, ^3^, ^4^, ^5^, ^6^] These surrogate molecules are easier to handle on a small scale than a comparable synthesis gas (a mixture of CO and H_2_) feedstock thus enabling a decentralized chemical industry. It comes as no surprise that this type of carbonylation has been used to synthesize a wide variety of organics ranging from industrially relevant carboxylic acids^[7]^ to pharmaceutical products.^[6]^ Often, “CO‐free” carbonylations are catalyzed by phosphine complexes of late transition metals (i.e., Pd, Rh, Ru or Ir).^[4]^

Although “CO‐free” carbonylation reactions have been successfully applied in organic synthesis^[8]^ relatively little is known about the precise reaction mechanism and the associated catalytic cycle.[^2^, ^9^, ^10^, ^11^] One of the main scientific questions that arises is whether CO is generated in situ or not. If CO gas is generated from the surrogate molecule then the “CO‐free” carbonylation reaction should proceed in the same way as a traditional carbonylation using synthesis gas.^[12]^ If another reaction intermediate is formed, the reaction mechanism of a “CO‐free” carbonylation could deviate from the established mechanism. Rosales et al.^[13]^ elaborated on this hypothesis by investigating the Rh‐catalyzed carbonylation of 1‐hexene with synthesis gas and formaldehyde and found that the selectivity of the reaction changed when formaldehyde was used. This observation hints at the possibility that “CO‐free” carbonylations have a different reaction mechanism than traditional carbonylation reactions, which goes beyond a simple surrogate decomposition reaction. However, in contrast to the study by Rosales et al., other studies focusing mainly on the ligand optimization and substrate scope of “CO‐free” carbonylation reactions hypothesized that formaldehyde and formic acid are decomposed into CO molecules, which are sequentially used in an alkoxy carbonylation of alkenes. This hypothesis is supported by experiments, which reveal the release of CO when no substrate is present.^[5]^

These contradicting results prompted the idea that reaction mechanisms based on CO, as well as based on other, CO‐equivalent species such as formyl groups are possible.^[2]^ As discussed, only a few reports have focused on the mechanistic investigation of “CO‐free” carbonylation reactions and those that do often do not apply molecular techniques^[14]^ but rather employ macroscopic approaches, for example, varying reactant ratios or ligand systems.[^2^, ^10^, ^11^, ^13^] Such a lack of mechanistic insight into this important class of carbonylation reactions prompted us to investigate the reaction mechanism of “CO‐free” carbonylations on the molecular level using both ex situ and in situ NMR spectroscopy. NMR spectroscopy is a powerful technique that enables quantification of multiple species at the same time while also providing significant chemical information to identify unknown intermediates.^[15]^

In this work, we have chosen to focus on the alkoxy carbonylation of 1‐octene using ^13^C‐paraformaldehyde (^13^C‐**PFA**), methyl formate, formic acid or phenyl formate in d^4^‐methanol (d^4^‐MeOH) applying the homogeneous catalyst [Pd(d^*t*^bpx)] (d^*t*^bpx=1,2‐Bis(di‐*tert*‐butylphosphino)xylene, **L**) as a model reaction (see Scheme 1). The chosen model reaction is a good example of “CO‐free” carbonylation reactions as 1‐octene is a chemical commonly processed in industry while d^*t*^bpx also finds application in industry. The associated product, methyl nonanoate, is used as a flavoring.^[17]^ The chosen reaction thus generalizes well in terms of industrial application. Furthermore, by using isotope labelled chemicals it becomes possible to selectively track specific molecular groups over the course of the reaction. Especially the application of ^13^C‐**PFA** is very beneficial to follow the track of the most relevant carbon atoms.

**Scheme 1:**
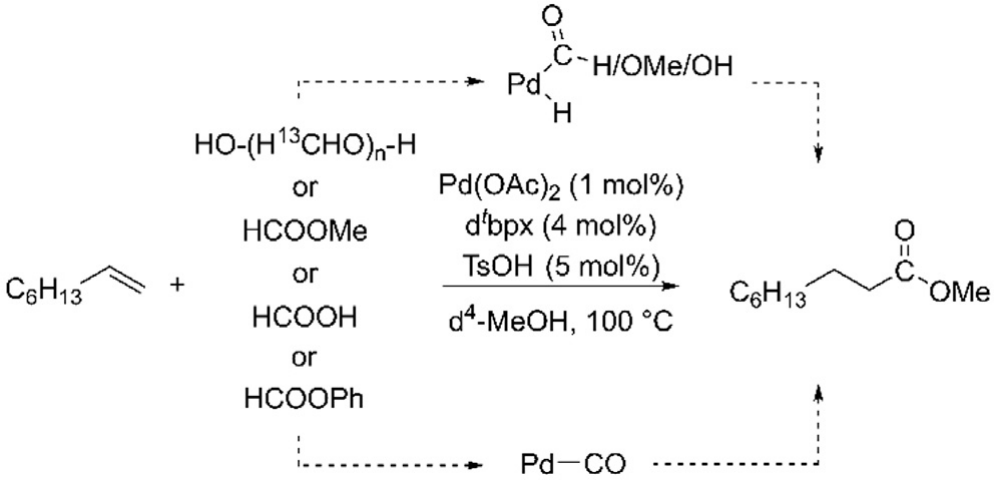
The model reaction studied in this work: Pd‐catalyzed alkoxycarbonylation of 1‐octene with ^13^C‐PFA, methyl formate, formic acid or phenyl formate in d^4^‐MeOH.

Figure 1 a shows ex situ ^1^H NMR spectra of the studied carbonylation reaction using ^13^C‐**PFA** (see also Figures S1–8). As expected, the ^1^H‐NMR spectra are dominated by the signals of 1‐octene in the alkyl region and at 5.41 ppm as well as the signals of MeOH at 3.35 and 4.91 ppm. When having a more detailed look it is possible to identify the intermediates H_2_^13^C(OMe)(OH) (**MM**; 4.65 ppm)^[18]^ H_2_^13^C(OMe)_2_ (**DMM**, 4.53 ppm)^[19]^ HCOOMe (**MF**, 8.07 ppm), the product methyl nonanoate (2.28 and 3.64 ppm), the precatalyst activator *p*‐toluenesulfonic acid (TsOH; 2.36, 7.21 and 7.70 ppm) and the protonated ligand **LH_2_** ^**2+**^ (7.48 and 7.74 ppm). Under the present conditions **DMM** is stable, indicating that it acts as a deactivation product rather than an intermediate.

**Figure 1:**
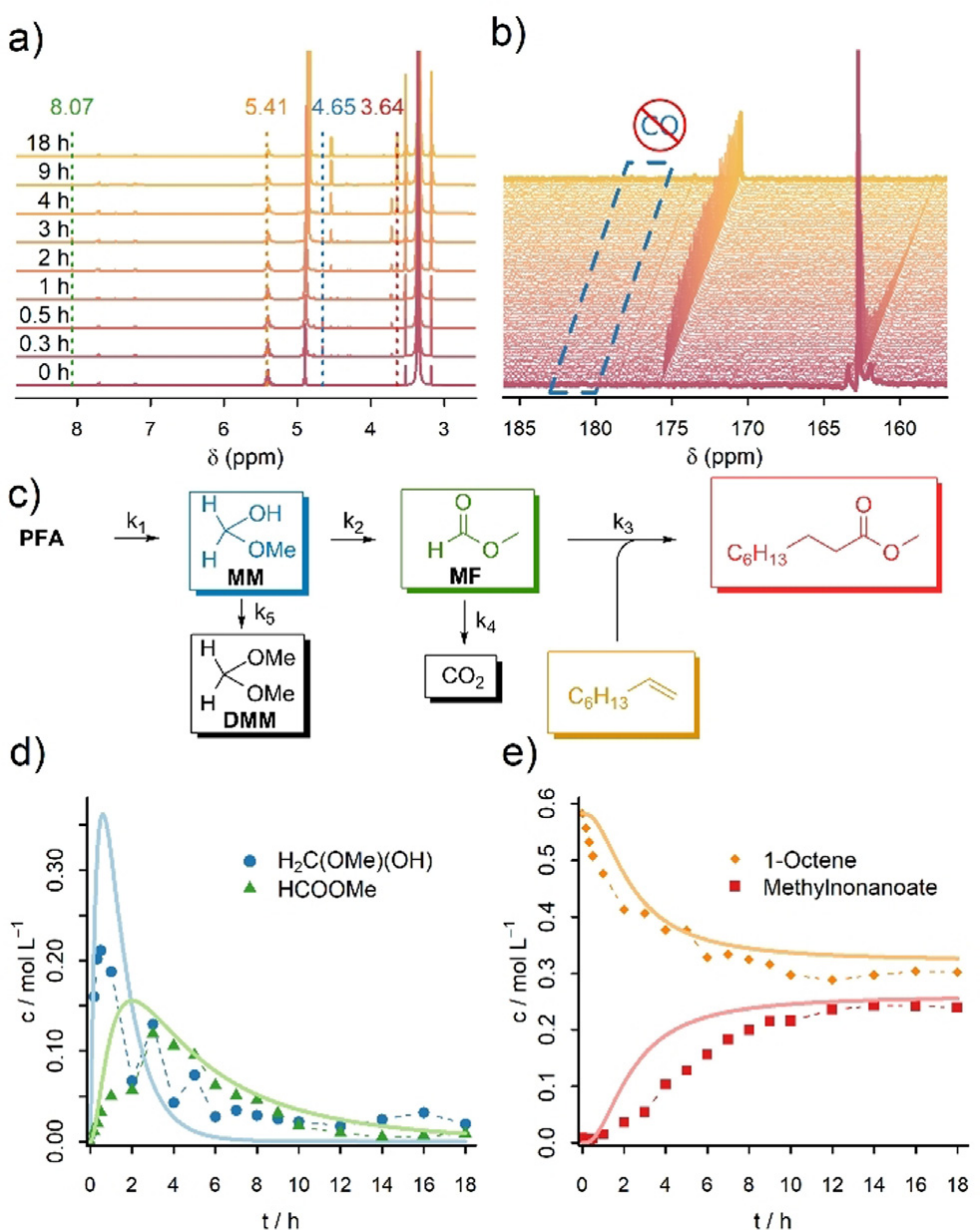
a) Selected ex situ ^1^H NMR spectra measured in d^4^‐MeOH. b) Selected in situ ^13^C NMR spectra in the carbonyl region. Importantly no CO signal (181–185 ppm^[16]^) is observed. c) Scheme illustrating the species considered for the microkinetic model, their possible reaction pathways and the respective kinetic rate constants. d,e) Data points and kinetic fits of **MM** (blue), **MF** (green), 1‐octene (orange) and methyl nonanoate (red) based on the time‐dependent concentrations extracted from ex situ ^1^H NMR spectra (signals annotated in a)).

As can be expected from the experimental design, the formaldehyde (hemi)formals **MM** and **DMM** show ^1^J(C−H) coupling. The same is partly true for **MF** where both a singlet and a doublet at 8.07 ppm (^1^ *J*(C−H)=226.6 Hz) are observed. The reason why ^13^C‐**MF** is not exclusively observed is due to a hydrogenation‐dehydrogenation equilibrium between formaldehyde and MeOH. Part of the solvent is dehydrogenated to form ^12^C‐formaldehyde and consequently ^12^C‐**MF**. The equilibrium was previously observed^[20]^ and is also visible in the ^1^ *J*(C‐D) coupling patterns in ex situ ^13^C spectra (see Figures S6).

The surprising result of the ex situ ^1^H NMR study is that **MF** is the key intermediate in the carbonylation reaction and that the reaction sequence for the relevant carbon atom is **PFA** → **MM** → **MF** + 1‐octene → methyl nonanoate. A similar reaction sequence can be observed when **MF** is used directly (see Figures S9–18). When formic acid (**FA**) or phenyl formate are used as cosubstrate, they undergo acidic transesterification with MeOH to form **MF** (see Figures S19–34). Furthermore, the ^13^C spectra show the formation of CO_2_ (126.1 ppm) as a side product. Surprisingly, and in contrast to previous hypotheses,[^5^, ^20^] no CO formation was detected. This is especially remarkable for phenyl formate which is known to easily decompose into CO and phenol.^[21]^

To exclude the possibility that CO gas is only formed at 100 °C and is thus not visible under ex situ conditions, in situ NMR experiments were performed (see Figures S35–42). When having a look at the carbonyl region of the in situ ^13^C spectra (Figure 1 b) it becomes clear that no CO signal is visible even under in situ conditions. Thus, it can be concluded that the “CO‐free” carbonylation of 1‐octene is indeed completely CO‐free, at least within the NMR time scale (limit of detection for CO in MeOH: 4 ppm, see Supporting Information for details). In a control experiment, the decomposition of ^13^C‐**MF** without 1‐octene was studied (see Figure S43). In this case CO is formed and bound in the form of [Pd(d^*t*^bpx)(CO)] (**Pd1**). Neither **Pd1** nor pure CO is observable when 1‐octene is present. This experiment proves that NMR spectroscopy is a suitable technique to study the appearance of CO containing species and that our experiments are in line with results obtained earlier via gas chromatography.^[5]^

In a second stage of the study, we were interested in a microkinetic model involving all intermediates to improve the speed of the reaction. As the concentration of **PFA** is not available from liquid state NMR we applied in situ Raman spectroscopy to extract its depolymerization kinetics at 100 °C.^[22]^ With the depolymerization rate constant at hand, we built a microkinetic model based on quantitative in situ Raman and ex situ NMR experiments (Figure 1 c). The experimental concentration profiles were fitted by varying the kinetic rate constants *k*_1_‐*k*_5_ and solving the underlying system of differential equations (see Supporting Information for details). The concentration profiles predicted by the microkinetic model fit the experimental data nicely (see Figure 1 d,e and Figure S44). Although a direct comparison of the extracted kinetic rate constants is difficult because the micro reactions are of different order, the model shows that under the present experimental conditions (i.e., excess of 1‐octene and low concentration of **MF**) the formation of methyl nonanoate from **MF** and 1‐octene seems to be the rate determining step in the reaction. In line with this result, the reaction is significantly faster (done in under 2 h, see Figures S11, S21 and S29) when **MF**, **FA** or phenyl formate are used directly as the concentration of **MF** is much higher in these cases.

To get further insight into the catalytically active species ex situ ^31^P NMR experiments were performed. Even at the beginning of the reaction a variety of phosphorous containing species can be found in the reaction solution. By synthesizing the reaction mixture step by step it became possible to identify [Pd(d^*t*^bpx)(MeOH)_2_]^2+^ (**Pd2**) and [Pd(η^2^‐CP‐d^*t*^bpx‐H)(η^2^‐OTs)]^+^ (**Pd3**) as the main Pd species at the beginning of the reaction which are formed from [Pd_2_(η^4^‐CP‐d^*t*^bpx)(μ‐OAc)_2_]_2_ (**Pd4**) and [Pd(η^2^‐CP‐d^*t*^bpx)(η^2^‐OAc)] (**Pd5**) when MeOH and TsOH are added (see Scheme 2 a and Figure S45–48). Furthermore, it comes as no surprise that the main phosphorous containing species are the mono and bis protonated ligands **LH^+^** and **LH_2_** ^**2+**^ as d^*t*^bpx and TsOH are used in excess. During the reaction [Pd(d^*t*^bpx)(μ‐H)]_2_ (**Pd6**), [Pd(d^*t*^bpx)(CHO)(H)]^+^ (**Pd7**) and [Pd(d^*t*^bpx)(C(O)OMe)(H)]^+^ (**Pd8**) can be identified based on their distinct NMR signals (see Scheme 2 and Table S1). **Pd6** features a hydride signal which is split into a quintet (43 Hz, see Figure S17) which can be explained by the presence of 4 magnetically equivalent P atoms.^[23]^ **Pd7** and **Pd8** are only observable under in situ conditions and are characterized by distinct ^13^C signals at 245 and 177 ppm, respectively. **Pd7** is—to the best of our knowledge—the first report of a Pd‐formyl complex.

**Scheme 2:**
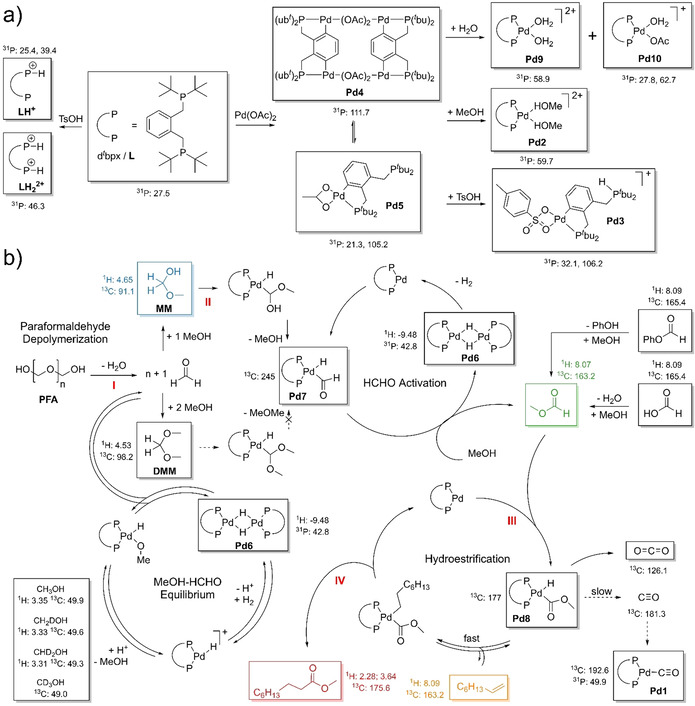
All species in boxes were directly observed in this study. Their characteristic NMR signals are given. a) Protonation and coordination chemistry of d^*t*^bpx. b) Proposed formyl mechanism for the hydroesterification of 1‐octene using **PFA**, **MF**, **FA** or phenyl formate as carbonyl source. The key steps are the depolymerization of **PFA** into formaldehyde (**I**), the C−H activation of formaldehyde to form methyl formate (**II**), the C−H activation of methyl formate (**III**) and the reductive elimination to form methyl nonanoate (**IV**).

Based on the quadruple reaction cycles proposed by Beller et al.^[20]^ the mechanistic insights of Clegg et al.[^24^, ^25^] as well as our results, we propose a new reaction mechanism for Pd‐catalyzed “CO‐free” carbonylations of alkenes (see Scheme 2 b). To differentiate it from the older hydride and methoxy carbonylation cycles^[24]^ we suggest to call it the formyl mechanism.

The reaction starts with the depolymerization of **PFA** to formaldehyde (**I**), which subsequently reacts with MeOH to form **MM** and **DMM. MM** and **DMM** can both undergo C−H activation but only **MM** can easily eliminate MeOH to form **Pd7** (**II**). **DMM** would need to eliminate MeOMe which is not possible. The formyl group then reacts with abundant MeOH to form the key intermediate **MF** and **Pd6** which eliminates H_2_ to regenerate [Pd(d^*t*^bpx)]. A similar reaction with Pd‐acyl groups was previously reported.^[26]^ **MF** is again C−H activated by [Pd(d^*t*^bpx)] to form **Pd8** (**III**). From there either a deactivation to CO_2_ is possible or the fast insertion of 1‐octene into the Pd−H bond to form [Pd(d^*t*^bpx)(COOMe)(C_8_H_17_)] or the slow decomposition into CO. Finally, a reductive elimination reaction takes place to form methyl nonanoate (**IV**). Based on this mechanism the reaction can be described as an hydroesterification rather than an alkoxycarbonylation. Our experiments prove that this reaction mechanism is very general as the most important C1 CO‐surrogate molecules (**PFA**, **FA** and **MF**) and phenyl formate follow this mechanism. As no major decomposition of the ligand system was observed, we assume that the formation of insoluble Pd black is the main deactivation pathway for the catalyst system. The formation of Pd black is visible after 18 h.

In the final stage of our work, we used quantum chemical simulations, that is, density functional theory (DFT, see Supporting Information for details), to validate the proposed mechanism. In particular, emphasis was set on modeling the key step of the C−H activation of **MF** and the possible subsequent reactions; details are shown in Scheme 3 as well as in Figure S49 and Table S2. The DFT calculations reveal that insertion of an alkene into the Pd−H bond (**III** to **IVa**) is energetically favored over the insertion into the Pd−C bond (**III** to **IVb**) (Δ*G* ^≠^=28.2 kJ mol^−1^ and Δ*G* ^≠^=156.0 kJ mol^−1^). Compared to the two insertion pathways, the decomposition of **MF** to either CO or CO_2_ is thermodynamically favored (Δ*G*=−38.9/Δ*H*=−78.9 kJ mol^−1^ (**III‐CO**) or Δ*G*=−121.9/Δ*H*=−147.7 kJ mol^−1^ **III‐CO_2_**), respectively), but the activation energies for the decompositions (Δ*G* ^≠^=118.0 and Δ*G* ^≠^=227.7 kJ mol^−1^) are much higher compared to the activation energies of the insertion. Comparison of the two decomposition pathways reveals that CO_2_ formation is thermodynamically favored over the formation of CO (Δ*G*=−130.8/Δ*H*=−102.8 kJ mol^−1^ for free CO_2_ (**V‐CO_2_**) and Δ*G*=48.5/Δ*H*=93.4 kJ mol^−1^ for free CO (**V‐CO**), respectively) but CO formation is kinetically favored (Δ*G* ^≠^=227.7 kJ mol^−1^ for **II** to **III‐CO_2_** and Δ*G* ^≠^=118.0 kJ mol^−1^ for **II** to **III‐CO**, respectively). At first, the lower activation energy for the CO formation, as predicted by DFT, are contradicted by our experimental observation where no CO formation was observed. However, if for some unknown reason CO is still formed and instantaneously consumed, the coordination of CO and subsequent insertion into the Pd−C bond are energetically possible (see Figure S49, Δ*G* ^≠^=59.8 kJ mol^−1^ and Δ*G* ^≠^=15.6 kJ mol^−1^, respectively) but the final elimination of the methyl ester has a high activation energy barrier (Δ*G* ^≠^=141.1 kJ mol^−1^). Concluding from the DFT calculations—and in line with our experimental observations—we favor the idea of the C−H activation of **MF** followed by direct insertion of 1‐octene into the Pd−H bond at **Pd8** and the subsequent elimination of the product.

**Scheme 3:**
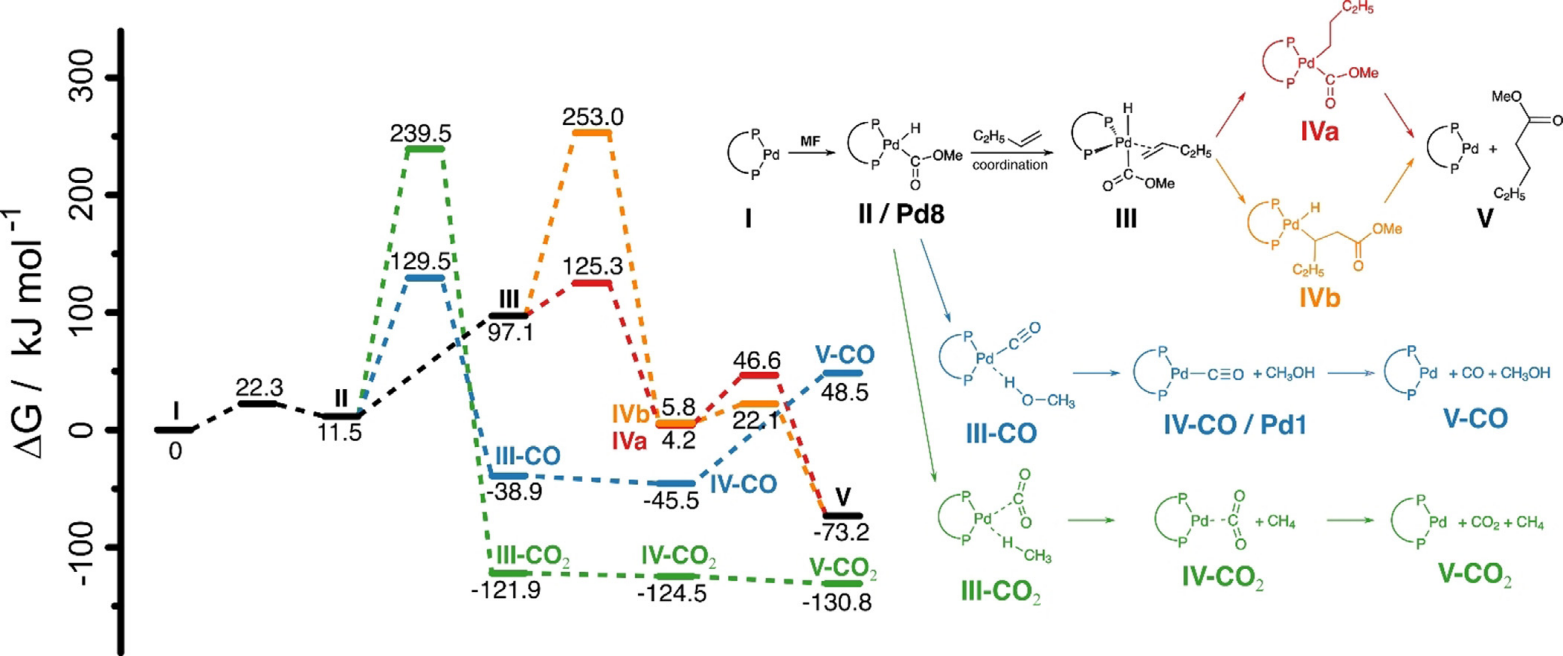
Reaction sequences and their respective Gibbs free energies and activation energies for the insertion (red and orange) as well as the CO_2_ and CO decomposition pathways (green and blue) as calculated by DFT (see Supporting Information for computational protocol).

In conclusion, we have presented a detailed mechanistic investigation of “CO‐free” carbonylation reactions using in situ spectroscopy and quantum chemical simulations. Based on the NMR experiments, methyl formate could be identified as the key intermediate independent of the starting material. Based on this information a microkinetic model, involving all intermediates, was developed and a new reaction mechanism was proposed based on the identification of many Pd‐d^*t*^bpx complexes. A highlight is the first direct observation of a Pd‐formyl complex. The experimental findings were supported by DFT calculations on the key steps of the proposed reaction mechanism. Importantly, this study showed that no CO is formed during the “CO‐free” hydroesterification of alkenes.

## Conflict of interest

The authors declare no conflict of interest.

## Supporting information

As a service to our authors and readers, this journal provides supporting information supplied by the authors. Such materials are peer reviewed and may be re‐organized for online delivery, but are not copy‐edited or typeset. Technical support issues arising from supporting information (other than missing files) should be addressed to the authors.

Supplementary
