# Supplementary material for: Reaction Mechanism of Pd‐Catalyzed “CO‐Free” Carbonylation Reaction Uncovered by In Situ Spectroscopy: The Formyl Mechanism
Source: Angew Chem Int Ed Engl. 2020 Dec 14;60(7):3422–7. doi: 10.1002/anie.202011152 (PMC7898928; doi:10.1002/anie.202011152)
Supplement: Supplementary file 1 — Supplementary [file ANIE-60-3422-s001.pdf]

Angewandte Chemie
Eine Zeitschrift der Gesellschaft Deutscher Chemiker

Supporting Information

Reaction Mechanism of Pd-Catalyzed “CO-Free” Carbonylation Reaction Uncovered by In Situ Spectroscopy: The Formyl Mechanism

*Robert Geitner, Andrei Gurinov, Tianbai Huang, Stephan Kupfer, Stefanie Gräfe, and Bert M. Weckhuysen**

anie_202011152_sm_miscellaneous_information.pdf

Table of Contents:

1. Experimental section 2
2. NMR assignments..7
3. Ex-situ NMR spectra 10
4. In-situ NMR spectra 44
5. Kinetic modeling 53
6. NMR spectra of simple mixtures..54
7. DFT calculated structures and energies...58
8. Pure component NMR spectra...59
9. Variable temperature NMR spectra..66
10. DFT calculated IR spectra 68
11. References 75

1. Experimental section

Chemicals

All manipulations were carried under argon atmosphere using standard Schlenk technique. 1,2-Bis(di-*tert*-butylphosphino)xylene (d^{t}bpx, **L**) was purchased from Sigma Aldrich and recrystallized from MeOH. d_4-MeOH and ^{13}C-paraformaldehyde (^{13}C-**PFA**) were purchased from Eurisotope. All other chemicals were purchased from Sigma Aldrich and used without further purification. 1-Octene was degassed via freeze-pump-thaw cycles, d_4-MeOH was dried over Mg/I_2, methyl formate was dried over K_2CO_3 then molsieves 3 Å, D_2CCl_2 was dried over $CaCl_2$ then molsieves 3 Å, and formic acid was dried over anhydrous $CuSO_4$.

NMR measurements

For reference purposes the ^{1}H and ^{13}C NMR spectra of HCOOMe, HCOOH, HCOOPh, 1-octene, methyl nonanoate, 1,3,5-trioxane and $H_2{}^{13}C(OMe)(OH)$ were recorded on an Agilent MRF400 or a Varian VNMR-S-400 400 MHz NMR spectrometer (see Figure S50 - Figure S62). Additionally the ^{1}H NMR spectra of MeOH at different temperatures (see Figure S63) and the dissolution of process of ^{13}C-PFA in d^4-MeOH (see Figure S64) were recorded on a Bruker 600 MHz AVANCE III NMR spectrometer equipped with a 5 mm H/C/N inverse probe. Ex-situ NMR spectra were recorded a Varian VNMR-S-400 400 MHz NMR spectrometer. For ex-situ measurements ^{1}H-NMR spectra were recorded with 32 scans, ^{13}C-NMR spectra with 256 scans and ^{31}P-NMR spectra with 128 scans. ^{13}C and ^{31}P spectra were ^{1}H decoupled with an inverse-gated-decoupling pulse scheme.

For a typical ex-situ experiment a Quick Pressure Valve 5 mm OD NMR Sample Tube (Wilmad-LabGlass, Vineland, New Jersey) was charged with $Pd(OAc)_2$ (1.33 mg, 5.92 µmol), d^{t}bpx (9.22 mg, 23.37 µmol), *p*-toluenesulfonic acid monohydrate ($TsOH{\cdot}H_2O$, 5.73 mg, 30.12 µmol) as well as ^{13}C-PFA (38.75 mg, 1.25 mmol). The tube was subsequently evacuated and flushed with argon three times before d_4-MeOH (0.45 mL) and 1-octene (0.1 mL, 0.64 mmol) were added. If methyl formate (0.08 mL, 1.31 mmol), formic acid (0.05 mL, 1.32 mmol) or phenyl formate (0.08 mL, 0.73 mmol) were used instead of ^{13}C-PFA the liquids were added after d^4-MeOH. The closed tube was heated to 100 °C (**Caution: The tube autopressurizes!**) and a set of NMR spectra was recorded at 25 °C after 0, 10, 20, 30, 60, 120, 180, 240, 300, 360, 420, 480, 540, 600, 720, 840, 960 and 1080 min (after 0, 120, 240, 360, 480, 600, 720, 840, 960 and 1080 min for HCOOMe, HCOOH and HCOOPh), respectively (see Figure S1 - Figure S34).

In-situ NMR experiments were performed on a Bruker 600 MHz AVANCE III NMR spectrometer. ^{1}H (~1.5 min) and ^{13}C spectra (~8.5 min) were recorded alternatingly during the catalytic reaction at 100 °C for 22 h. 2x 129 spectra were recorded (see Figure S35 - Figure S42). The ^{13}C NMR spectra were ^{1}H decoupled with an inverse-gated-decoupling scheme.

During initial in-situ experiments the ^{13}C-PFA dissolution process caused bubbling inside the tube at elevated temperatures. Thus, it was not possible to acquire in-situ NMR spectra with solid ^{13}C-PFA. To overcame this experimental challenge ^{13}C-PFA (200 mg) was dissolved in d_4-MeOH (2 mL). The NMR tube was charged with $Pd(OAc)_2$ (3.81 mg, 16.97 µmol), d^{t}bpx (26.46 mg, 67.06 µmol), $TsOH{\cdot}H_2O$ (16.45 mg, 86.48 µmol), evacuated and flushed with argon three times before adding ^{13}C-PFA in d_4-MeOH (0.20 mL, 0.65 mmol $H^{13}CHO$), d_4-MeOH (0.45 mL) and 1-octene (0.1 mL, 0.64 mmol). Similar experiments were performed with methyl formate (0.08 mL, 1.31 mmol) and formic acid (0.05 mL, 1.32 mmol) as carbonylation agents.

To assign unknown NMR signals simplified reaction solutions were synthesized. d^{t}bpx (20.05 mg, 89.31 µmol) was mixed with 1 and 4 equivalents of TsOH·H_2O (17.05 mg, 89.63 µmol or 69.11 mg, 363.32 µmol) in d^4-MeOH (0.5 mL) (see Figure S45 - Figure S47). This experiment enabled the identification of the protonated ligand. Furthermore, $Pd(OAc)_2$ (8.42 mg, 37.50 µmol), d^{t}bpx (14.80 mg, 37.51 µmol) were mixed in D_2CCl_2 (0.5 mL) (see Figure S48). In a subsequent experiment ($Pd(OAc)_2$ (13.75 mg, 61.25 µmol), d^{t}bpx (23.59 mg, 59.79 µmol), D_2CCl_2 (0.5 mL)) TsOH·H_2O (12.44 mg, 65.40 µmol) was added to check the coordination chemistry of Pd^{2+} ions, d^{t}bpx and TsOH in a non-coordinating solvent. A surprising variety of Pd-d^{t}bpx complexes was identified by comparison with literature reports (see Table S1). Finally, the decomposition of $H^{13}COOMe$ (0.08 mL, 1.30 mmol) at 100 °C overnight in d^4-MeOH (0.45 mL) in the presence of $Pd(OAc)_2$ (2.21 mg, 9.84 µmol), d^{t}bpx (15.50 mg, 39.28 µmol) and TsOH·H_2O (9.34 mg, 49.10 µmol) was tested. In agreement with the control experiments by Beller et al.[1] CO_2 and CO are formed which is bound in [Pd(d^{t}bpx)(CO)] (see Figure S43). This is in contrast the ex-situ and in-situ experiments when 1-octene is present as no signal from coordinated nor uncoordinated CO can be found. This experiment further proves that NMR spectroscopy together with isotope labelled substrates is a suitable technique to study the presence of CO.
All NMR spectra show small contributions from the oxidized phosphine ligand. These contributions are unfortunately unavoidable and stem from the sample preparation process.
To determine the limit of detection for CO gas via NMR different CO solutions in MeOH were prepared. The solutions were made by pressurizing degassed d_4-MeOH with CO gas (611, 708, 804, 899 and 1013 mbar). According to Henry's law

$$c = \frac{p}{K}$$

where c is the concentration, p the pressure and K the Henry coefficient, the concentration of a gas dissolved in a solvent scales linearly with its pressure above the solution. For the system CO/MeOH the Henry coefficient K at 25 °C is 12100 Pa L mol^{-1}[2] translating the aforementioned CO pressures to concentrations of 5.0, 5.9, 6.6, 7.4 and 8.4 mmol L^{-1}, respectively. As normal CO gas was used only 1 % of the gas molecules were NMR active ^{13}CO molecules. To nevertheless record a reasonable ^{13}C-NMR signal 1024 scans were used. Subsequently the NMR spectra were normalized to the d_4-MeOH signal and the CO signals at 185.4 ppm were integrated from 185.3 to 185.5 ppm. The resulting calibration curve is defined by

$$P_{CO} = m \cdot c_{CO} + b$$

Where P_{CO} is the integrated peak area, c_{CO} the CO concentration, m the slope of the calibration curve and b the intercept. The calibration curve can be seen in Figure SX. To determine the limit of detection for CO in MeOH a blank d_4-MeOH sample was measured. Subsequently the standard deviation σ_B of the noise integrated inside the same spectral window as the CO signal (0.2 ppm) of the blank sample was calculated. From σ_B the limit of detection LOD can be calculated by using the calibration curve parameters m and b[3]

$$LOD = \frac{3\sigma_B - b}{m}$$

Under the chosen experimental conditions (1024 scans, ordinary CO with 1 % ^{13}CO) this results in a LOD of 5.5 mM, 196 weight ppm or 670 mbar.

This result can be translated to the chosen ex-situ measurement conditions (256 scans, 100 % ^{13}C-PFA as potential ^{13}CO source) via the following equation[4]

$$SNR \propto n\gamma_e\sqrt{\gamma_d^3 B_0^3 t}$$

where n is the number of nuclear spins being observed, γ_e is the gyromagnetic ratio of the spin being excited, γ_d is the gyromagnetic ratio of the spin being detected, B_0 is the magnetic field strength, and t is the experiment acquisition time. As can be seen from the equation the SNR scales linearly with the number of NMR-active nuclei and with a square-root dependency for the measurement time. Thus an enhancement factor can be derived

$$\frac{SNR_1}{SNR_2} = \frac{n_1\gamma_e\sqrt{\gamma_d^3 B_0^3 t_1}}{n_2\gamma_e\sqrt{\gamma_d^3 B_0^3 t_2}} = \frac{n_1\sqrt{t_1}}{n_2\sqrt{t_2}}$$

This enhancement factor becomes 50 if the above described experimental conditions are used in the equation and the spectra were recorded on the same NMR spectrometer (the number of scans is proportional to the measurement time):

$$\frac{SNR_1}{SNR_2} = \frac{100\sqrt{256}}{1\sqrt{1024}} = 50$$

Thus with the chosen parameters NMR spectroscopy is 50 times more sensitive for the detection of CO. With this the LOD for the conducted ex-situ experiments can be directly calculated. The LOD becomes 0.1 mM, 4 weight ppm or 13 mbar when 100 % ^{13}CO and 256 scans at 25 °C are utilized for the ^{13}C-NMR experiments.

Data processing

NMR spectra were referenced, background and phase corrected with MestReNova (10.0.2-15465).[5] ^{1}H and ^{13}C-NMR spectra were referenced to the residual solvent signal (MeOH in d^{4}-MeOH: 3.35 or 49.85 ppm respectively).[6] All NMR spectra were background corrected with a third order Bernstein polynomial.[7]
The subsequent kinetic analysis and data visualization was done using R (4.0.2)[8] and the R packages deSolve[9], FME[10], RColorBrewer[11] and colorspace[12].
For the kinetic analysis the ^{1}H and ^{13}C spectra were normalized to one of the aromatic OTs^- signals between 7.680 and 7.738 or 129.58 and 129.92 ppm, respectively. The tosylate anion OTs^- does not participate in the catalytic hydroesterification reaction and thus can be used as an internal standard. The following integration borders were used to extract the relative amounts of substance and therefore the concentration profiles: 2.210 and 2.324 (C_7H_{15}-C$\mathbf{H_2}$-C(O)OCH_3); 4.496 and 4.570 ($\mathbf{H_2}$C$(OCH_3)_2$); 4.609 and 4.633 (**H**-DC(OCH_3)(OH)); 4.633 and 4.668 ($\mathbf{H_2}$C(OCH_3)(OH)); 5.250 and 5.498 (C_6H_{13}-C**H**=C$\mathbf{H_2}$); 7.770 and 7.830 (**H**-^{13}C(O)OCH_3); 8.033 and 8.099 ppm (**H**-^{12}C(O)OCH_3). The CO_2 signal was integrated in the ^{13}C spectra between 126.02 and 126.21 ppm.

Kinetic model

The concentration data extracted from the ^{1}H NMR measurements was fitted with the following system of differential equations:

$$\frac{d[PFA]}{dt} = -k_1 \cdot [PFA]$$

$$\frac{d[MM]}{dt} = +k_1 \cdot [PFA] - k_2 \cdot [MM] - k_5 \cdot [MM]$$

$$\frac{d[MF]}{dt} = +k_2 \cdot [MM] - k_3 \cdot [MF] \cdot [O] - k_4 \cdot [MF]$$

$$\frac{d[P]}{dt} = +k_3 \cdot [MF] \cdot [O]$$

$$\frac{d[O]}{dt} = -k_3 \cdot [MF] \cdot [O]$$

$$\frac{d[CO_2]}{dt} = +k_4 \cdot [MF]$$

$$\frac{d[DMM]}{dt} = +k_5 \cdot [MM]$$

where **PFA** stands for paraformaldehyde, **MM** for methoxy methanol ($H_2C(OMe)(OH)$), **MF** for methyl formate, P for methyl nonanoate, O for 1-octene, CO_2 for carbon dioxide and **DMM** for dimethoxy methane ($H_2C(OMe)_2$). k_1-k_5 were extracted by fitting the experimental concentration profiles for each of the seven species with the system of differential equations. As the concentration of **PFA** was not directly available from the NMR spectra the concentration profile of **PFA** was simulated by using the previously extracted kinetic rate constant for the **PFA** depolymerization (0.001997 s^{-1}).[13] The concentration of CO_2 was also not available from ^{1}H NMR spectra. Thus, the relative concentration from its ^{13}C peak was used, adjusted by a semi-quantitative factor of 0.52 to account for its increased ^{13}C content compared to OTs^- (which is used to normalize the ^{13}C spectra.). To account for the excess of 1-octene used the model was fitted with a modified 1-octene concentration: The final 1-octene concentration at 1080 min was subtracted from the general 1-octene concentrations for the fitting process. For visualization the final 1-octene concentration was added to the experimental and fitted concentration profiles again. All experimental concentration profiles and the resulting fits and the extracted kinetic rate constants are shown in Figure S44.

Quantum chemical calculations

To further investigate the reaction mechanism following the C-H activation of methyl formate, quantum chemical calculations were performed. To model the chemical structure of 1-octene at reasonable computational demand the alkene was approximated by 1-butene.
All quantum chemical simulations were performed using the Gaussian16 software.[14] The ground state equilibrium structures and electronic properties were obtained at the density functional (DFT) level of theory utilizing the B3LYP XC functional;[15] i.e. of the substrate

molecules HCOOMe, C_4H_8, C_4H_9COOMe, MeOH, CH_4, CO and CO_2, the initial palladium complex [Pd(d^{t}bpx)] (**I**) and its methyl formate adduct [Pd(d^{t}bpx)H(COOMe)] (**II**) as well of the intermediates and product states along the alkene insertion route [Pd(d^{t}bpx)H(COOMe)(C_4H_8)], [Pd(d^{t}bpx)(C_4H_9)(COOMe)], [Pd(d^{t}bpx)H(C_4H_9COOMe)] (**III**, **IVa** and **IVb**, respectively). Furthermore, the methyl formate decomposition pathways via carbon monoxide ([Pd(d^{t}bpx)(CO)(MeOH)] and [Pd(d^{t}bpx)(CO)] (**III-CO** – **IV-CO**)) and carbon dioxide ([Pd(d^{t}bpx)(CO_2)(CH_4)] and [Pd(d^{t}bpx)(CO_2)] (**III-CO$_2$** – **IV-CO$_2$**)) were studied (see Scheme 3). Finally, the alternative alkoxycarbonylation route and its complexes [Pd(d^{t}bpx)H]$^+$, [Pd(d^{t}bpx)(C_4H_9)]$^+$, [Pd(d^{t}bpx)(C_4H_9)(CO)]$^+$, [Pd(d^{t}bpx)(C(O)C_4H_9)]$^+$ (**I-AC** – **IV-AC**) were considered (see Figure S49). The def2-SVP basis set as well as the respective core potentials were applied for all atoms.[16] A subsequent vibrational analysis was carried out for each optimized ground state structure to verify that a minimum on the potential energy (hyper-)surface (PES) was obtained. All calculations were performed including D3 dispersion correction with Becke-Johnson damping.[17]

An analogous computational setup was applied for the optimization of transition states (TSs), while an initial guess in the vicinity of the saddle point was at first obtained via the Nudged Elastic Band (NEB)[18] method as implemented in pysisyphus[19] with xtb.[20] Thereafter, the TSs were obtained in Gaussian16 via the Berny algorithm,[21] followed by a vibrational analysis to verify that a first-order saddle point on the PES was obtained.

All optimized structures (xyz files) and the corresponding Gaussian output (log files) can be found online at the open-source repository of the European Commission *Zenodo* (DOI: 10.5281/zenodo.4153003).

To achieve the realistic visualization of the calculated vibrational spectra from the calculated IR intensities shown in Figure S65 - Figure S78, the intensities were fitted with Voigt functions (full width at half maximum (FWHM): 18 cm^{-1}). It was assumed that the Gaussian and Lorentzian part contribute equally to the Voigt function. Thus, both convoluted functions featured a FWHM of 11 cm^{-1}, which leads to the described FWHM of 18 cm^{-1} for the resulting Voigt function.[22]

2. NMR assignments

*$H_2C(OCH_3)(OH)$, **MM***[23]
^{1}H-NMR 400 MHz (d^4-MeOH, 25 °C): 3.33 (s, 3H, CH_3); 4.65 (s, ^{13}C satellites: d, $^1J_{CH}$ = 161.7 Hz, 2H, CH_2) ppm.
^{13}C{^{1}H}-NMR 100 MHz (d^4-MeOH, 25 °C): 55.0; 91.1 ppm.

*$H_2C(OCH_3)_2$, **DMM***[24]
^{1}H-NMR 400 MHz (d^4-MeOH, 25 °C): 3.33 (s, 6H, CH_3); 4.53 (s, ^{13}C satellites: d, $^1J_{CH}$ = 162.4 Hz, 2H, CH_2) ppm.
^{13}C{^{1}H}-NMR 100 MHz (d^4-MeOH, 25 °C): 55.2; 98.2 ppm.

*HCOOMe, **MF***
^{1}H-NMR 400 MHz (d^4-MeOH, 25 °C): 3.72 (s, 3H, CH_3); 8.09 (s, 1H, ^{13}C satellites: d, $^1J_{CH}$ = 226.3 Hz, HCOO) ppm.
^{13}C{^{1}H}-NMR 100 MHz (d^4-MeOH, 25 °C): 51.1; 163.2 ppm.

*HCOOH, **FA***
^{1}H-NMR 400 MHz (d^4-MeOH, 25 °C): 8.09 (s, 1H, ^{13}C satellites: d, $^1J_{CH}$ = 218.4 Hz) ppm.
^{13}C{^{1}H}-NMR 100 MHz (d^4-MeOH, 25 °C): 165.4 ppm.

HCOOPh
^{1}H-NMR 400 MHz (d^4-MeOH, 25 °C): 7.11 (m, 2H, *o/m*-CH-Ph); 7.23 (m, 1H, *p*-CH-Ph); 7.37 (m, 2H, *o/m*-CH-Ph); 8.32 (s, 1H, HCOO) ppm.
^{13}C{^{1}H}-NMR 100 MHz (d^4-MeOH, 25 °C): 120.9; 126.0; 129.4; 150.2; 160.0 ppm.

1-Octene
^{1}H-NMR 400 MHz (d^4-MeOH, 25 °C): 0.90 (t, $^3J_{HH}$ = 6.9 Hz, 3H, CH_3); 1.34 (m, 8H, CH_2); 2.04 (m, 2H); 4.92 (m, 2H, $CH{=}CH_2$); 5.80 (m, 1H, $CH{=}CH_2$) ppm.
^{13}C{^{1}H}-NMR 100 MHz (d^4-MeOH): 14.5; 23.7; 30.0; 30.1; 32.9; 34.9; 49.0; 114.7; 140.0 ppm.

Methyl nonanoate
^{1}H-NMR 400 MHz (d^4-MeOH, 25 °C): 0.90 (t, $^3J_{HH}$ = 7.0 Hz, 3H, CH_3); 1.31 (m, 10H, CH_2); 1.61 (m, 2H); 2.31 (t, $^3J_{HH}$ = 7.5 Hz, 2H); 3.65 (s, 3H) ppm.
^{13}C{^{1}H}-NMR 100 MHz (d^4-MeOH, 25 °C): 14.5; 23.7; 26.0; 30.2; 30.3; 30.4; 33.0; 34.8; 49.0; 51.9; 175.6 ppm.

1,3,5-Trioxane
^{1}H-NMR 400 MHz (d^4-MeOH, 25 °C): 5.13 (s, 2H, CH_2) ppm.
^{13}C{^{1}H}-NMR 100 MHz (d^4-MeOH, 25 °C): 94.6 ppm.

MeOH (temperature dependency)
^{1}H-NMR 400 MHz (d^4-MeOH, 25 °C): 3.35 (s, 3H, CH_3); 4.90 (s, 1H, OH) ppm.
^{1}H-NMR 400 MHz (d^4-MeOH, 35 °C): 3.35 (s, 3H, CH_3); 4.79 (s, 1H, OH) ppm.
^{1}H-NMR 400 MHz (d^4-MeOH, 45 °C): 3.35 (s, 3H, CH_3); 4.67 (s, 1H, OH) ppm.
^{1}H-NMR 400 MHz (d^4-MeOH, 55 °C): 3.35 (s, 3H, CH_3); 4.55 (s, 1H, OH) ppm.
^{1}H-NMR 400 MHz (d^4-MeOH, 65 °C): 3.35 (s, 3H, CH_3); 4.43 (s, 1H, OH) ppm.
^{1}H-NMR 400 MHz (d^4-MeOH, 75 °C): 3.35 (s, 3H, CH_3); 4.30 (s, 1H, OH) ppm.
^{1}H-NMR 400 MHz (d^4-MeOH, 85 °C): 3.35 (s, 3H, CH_3); 4.19 (s, 1H, OH) ppm.

^{1}H-NMR 400 MHz (d^4-MeOH, 90 °C): 3.35 (s, 3H, CH_3); 4.13 (s, 1H, OH) ppm.
^{1}H-NMR 400 MHz (d^4-MeOH, 100 °C): 3.35 (s, 3H, CH_3); 4.02 (s, 1H, OH) ppm.

CH_3OH
^{1}H-NMR 400 MHz (d^4-MeOH, 25 °C): 3.35 (s, 3H, CH_3); 4.90 (s, 1H, OH) ppm.
^{13}C{^{1}H}-NMR 100 MHz (d^4-MeOH): 49.85 (s) ppm.

CH_2DOH
^{1}H-NMR 400 MHz (d^4-MeOH, 25 °C): 3.33 (t, *J*(H-D) = 1.6 Hz, 2H, CH_3); 4.90 (s, 1H, OH) ppm.
^{13}C{^{1}H}-NMR 100 MHz (d^4-MeOH, 25 °C): 49.57 (t, *J*(C-D) = 21.5 Hz) ppm.

CHD_2OH
^{1}H-NMR 400 MHz (d^4-MeOH, 25 °C): 3.31 (quin, *J*(H-D) = 1.6 Hz, 1H, CH_3); 4.90 (s, 1H, OH) ppm.
^{13}C{^{1}H}-NMR 100 MHz (d^4-MeOH, 25 °C): 49.28 (quin, *J*(C-D) = 21.5 Hz) ppm.

CD_3OH
^{1}H-NMR 400 MHz (d^4-MeOH, 25 °C): 4.90 (s, 1H, OH) ppm.
^{13}C{^{1}H}-NMR 100 MHz (d^4-MeOH, 25 °C): 49.00 (hep, *J*(C-D) = 21.5 Hz) ppm.

[H-d^{t}bpx]$^+$, ***LH$^+$***
^{31}P{^{1}H}-NMR 161 MHz (d^4-MeOH): 25.4 (br, 1P, P); 39.4 (br, 1P, P-H^+) ppm.

[2H-d^{t}bpx]$^{2+}$, ***LH_2^{2+}***
^{1}H-NMR 400 MHz (d^4-MeOH): 1.41 (d, $^3J_{HP}$ = 16.7 Hz, 36H, CH_3); 4.06 (d, $^2J_{HP}$ = 14.4 Hz, 4H, CH_2); 7.39 (m, 2H, CH); 7.57 (m, 2H, CH) ppm.
^{13}C{^{1}H}-NMR 100 MHz (d^4-MeOH): 20.1 (d, $^1J_{CP}$ = 39.3 Hz, 2C, CH_2); 27.8 (s, 12C, C(**C**H_3)$_3$); 34.7 (d, $^1J_{CP}$ = 33.3 Hz, 4C, **C**(CH_3)$_3$); 130.5 (m, 2C, C-Ph); 131.3 (dd, J_{CP} = 7.9 and 6.1 Hz, 2C, CH-Ph); 133.2 (dd, J_{CP} = 6.9 and 1.3 Hz, 2C, CH-Ph) ppm.
^{31}P{^{1}H}-NMR 161 MHz (d^4-MeOH): 46.3 (br) ppm.

[Pd(d^{t}bpx)(CO)], ***Pd1*** *(tentative, based on[25,26])*
^{13}C{^{1}H}-NMR 100 MHz (d^4-MeOH, 25 °C): 192.6 (s, CO) ppm.
^{31}P{^{1}H}-NMR 161 MHz (d^4-MeOH, 25 °C): 49.9 (s) ppm.

[Pd(η^2-CP-d^{t}bpx-H)(η^2-OTs)][OTs], ***Pd3*** *(tentative, based on[27])*
^{31}P{^{1}H}-NMR 161 MHz (D_2CCl_2, 25 °C): 32.2 (br), 106.2 (br) ppm.

[Pd(η^2-CP-d^{t}bpx)(η^2-OAc)], ***Pd5*** *(tentative, based on[27])*
^{31}P{^{1}H}-NMR 161 MHz (d^4-MeOH, 25 °C): 21.3 (br), 105.2 (br) ppm.

[Pd(d^{t}bpx)(μ-H)]$_2$, ***Pd6*** *(tentative, based on[25,28])*
^{1}H-NMR 400 MHz (d^4-MeOH, 25 °C): -9.48 (quin, J_{HP} = 43 Hz, 1H, Pd-H-Pd) ppm.
^{31}P{^{1}H}-NMR 161 MHz (d^4-MeOH, 25 °C): 42.8 (br) ppm.

[Pd(d^{t}bpx)(CHO)(H)]$^+$, ***Pd7*** *(tentative, based on[29])*
^{13}C{^{1}H}-NMR 100 MHz (d^4-MeOH, 100 °C): 245 (s) ppm.

[Pd(d^{t}bpx)(C(O)OMe)(H)]$^+$, ***Pd8*** *(tentative, based on[30])*

$^{13}C\{^{1}H\}$-NMR 100 MHz (d^4-MeOH, 100 °C): 177 (s) ppm.

Table S1. Aggregation of NMR data from this work and literature references about d^{t}bpx, similar phosphines, phosphonium salts, oxidation products as well as Pd-d^{t}bpx complexes. Information originates from this work if no reference is given.

Compound	Abb	Solvent	δP_A	δP_B	δH	δC
d^{t}bpx	**L**	CD_3OD	27.5	-	-	-
$PH(^tBu)_2$[31]		THF-d_8	19.7	-	-	-
$P(CH_3)(^tBu)_2$[32]		$DCCl_3$	12.2	-	-	-
$P(CH_2Ph)(^tBu)_2$[33]		$DCCl_3$	35.9	-	-	-
$[HPH(^tBu)_2][AlBr_4]$[34]		C_6D_6	24.2	-	-	-
$[HP(CH_3)(^tBu)_2]^+$[35]		C_6D_6	25.0	-	-	-
[H-d^{t}bpx][OTs]	**LH^+**	CD_3OD	25.4	39.4	-	-
[2H-d^{t}bpx]$[OTs]_2$	**LH_2^{2+}**	CD_3OD	46.3	-	-	-
d^{t}bpx-ox_2[36]	**Lox_2**	$DCCl_3$	63.5	-	-	-
[Pd(d^{t}bpx)$(H_2O)_2]^{2+}$[37]		THF-d_8	53.8	-	-	-
[Pd(d^{t}bpx)$(H_2O)_2]^{2+}$	**Pd9**	D_2CCl_2	58.9	-	-	-
[Pd(d^{t}bpx)$(MeOH)_2]^{2+}$	**Pd2**	CD_3OD	59.7	-	-	-
[Pd(d^{t}bpx)$(H_2O)(OAc)]^+$	**Pd10**	D_2CCl_2	27.8	62.7	-	-
[Pd(d^{t}bpx)(CO)][25,26]		C_7D_8	50.0	-	-	n.d.
[Pd(d^{t}bpx)(CO)]	**Pd1**	CD_3OD	49.9	-	-	192.6
[Pd(d^{t}bpx)$(H)(MeOH)]^+$[37]		CD_3OD	23.9	75.7	-10.00	
[Pd(d^{t}bpx)$(H)(CO)]^+$[37]		CD_3OD	30.7	60.3	-5.3	183.3
[Pd(d^{t}bpx)(η^2-OTs)][OTs][37]		CD_3OD	70	-	-	-
$[Pd_2$(d^{t}bpx)$_2$(μ-H)(μ-CO)][25]		C_6H_5Cl	42.0	-	-9.96	n.d.
[Pd(d^{t}bpx)(μ-H)$]_2$	**Pd6**	CD_3OD	42.8	-	-9.48	-
[Pd(d^{t}bpx)$(CHO)(H)]^+$	**Pd7**	CD_3OD	n.d.	n.d.	-	245
[Pd(d^{t}bpx)$(C(O)OMe)(H)]^+$	**Pd8**	CD_3OD	n.d.	n.d.	-	177
[Pd(η^2-CP-d^{t}bpx-H)(η^2-OTf)][OTf][37]		CD_3OD	42.4	105.6	-	-
$[Pd_2(\eta^4$-CP-d^{t}bpx)(μ-OAc$)_2]_2$[38]	**Pd4**	$DCCl_3$	111.7	-	-	-
[Pd(η^2-CP-d^{t}bpx)(η^2-OAc)]	**Pd5**	CD_3OD	21.3	105.2	-	-
[Pd(η^2-CP-d^{t}bpx)(η^2-OAc)]		$DCCl_3$	21.6	108.5	-	-
[Pd(η^2-CP-d^{t}bpx-H)(η^2-OTs)][OTs]	**Pd3**	D_2CCl_2	32.1	106.2	-	-

3. Ex-situ NMR spectra

^{13}C-paraformaldehyde

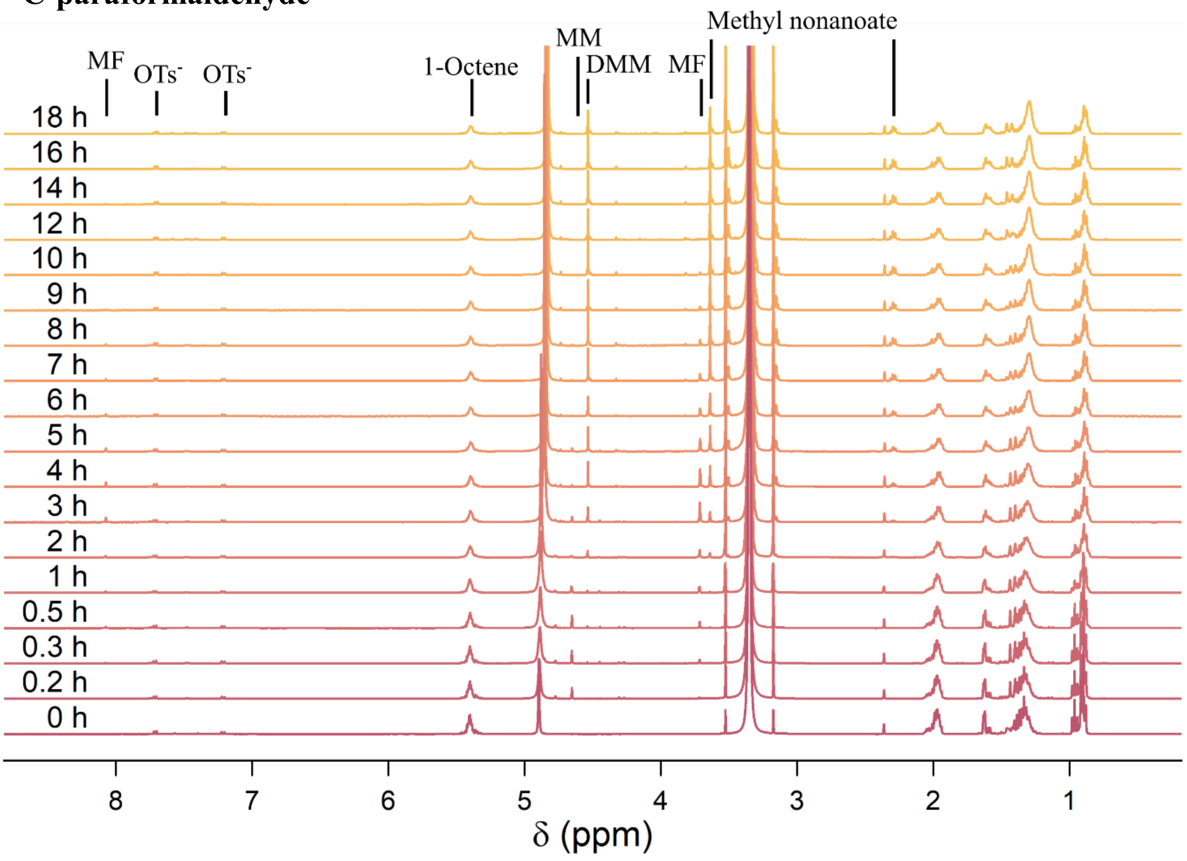

Figure S1. Ex-situ, background corrected ^{1}H-NMR spectra recorded at 25 °C of the hydroesterification of 1-octene with ^{13}C-PFA and [Pd(d^{t}bpx)] in d^4-MeOH. Field of view from 0.5 to 8.5 ppm. Legend: **MM** = $H_2C(OMe)(OH)$, **DMM** = $H_2C(OMe)_2$, **MF** = HCOOMe.

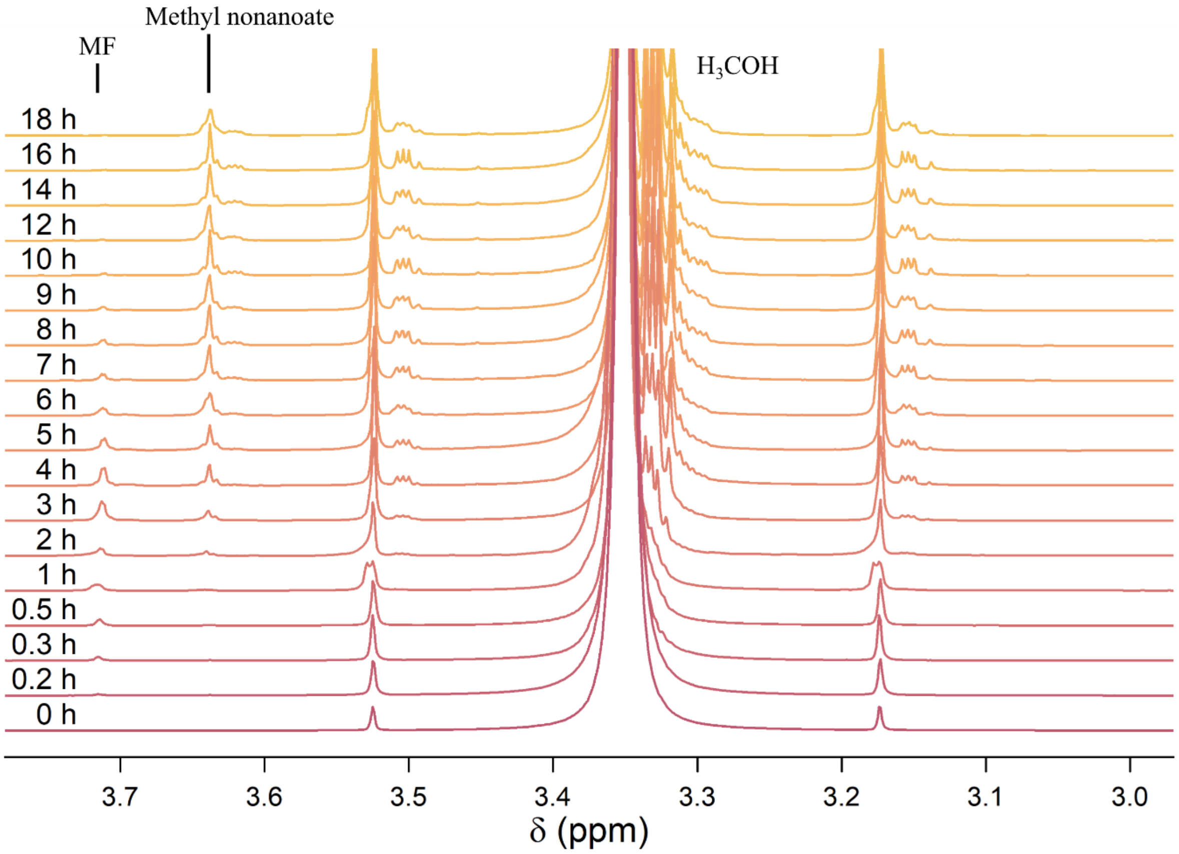

Figure S2. Ex-situ, background corrected ^{1}H-NMR spectra recorded at 25 °C of the hydroesterification of 1-octene with ^{13}C-PFA and [Pd(d^{t}bpx)] in d^{4}-MeOH. Field of view from 3.0 to 3.75 ppm. The methanol signal at 3.35 ppm and its ^{13}C satellites show changing C-D couplings, indicating a constant hydrogenation/dehydrogenation reaction. Legend: **MF** = HCOOMe.

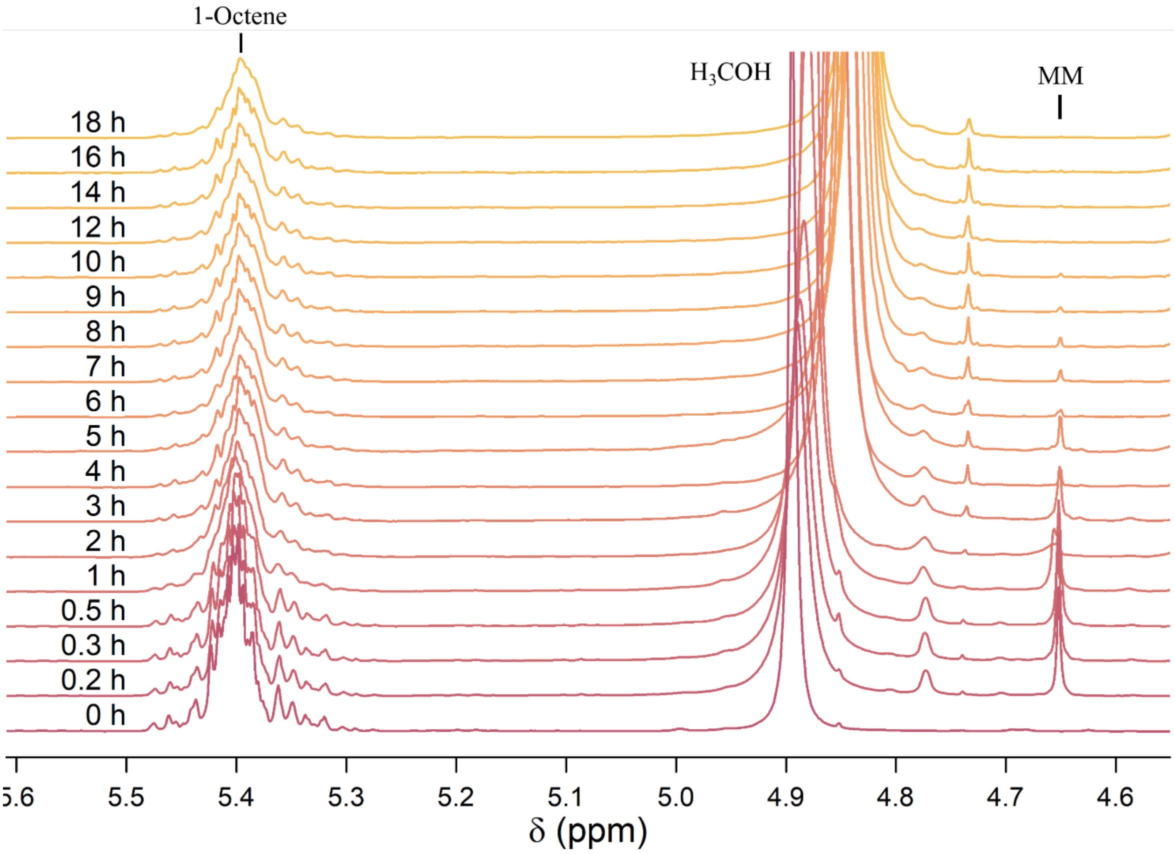

Figure S3. Ex-situ, background corrected ^{1}H-NMR spectra recorded at 25 °C of the hydroesterification of 1-octene with ^{13}C-PFA and [Pd(d^{t}bpx)] in d^{4}-MeOH. Field of view from 4.5 to 5.6 ppm. The signal of the alkenyl protons in 1-octene is decreasing over the course of the reaction. Legend: **MM** = $H_2C(OMe)(OH)$.

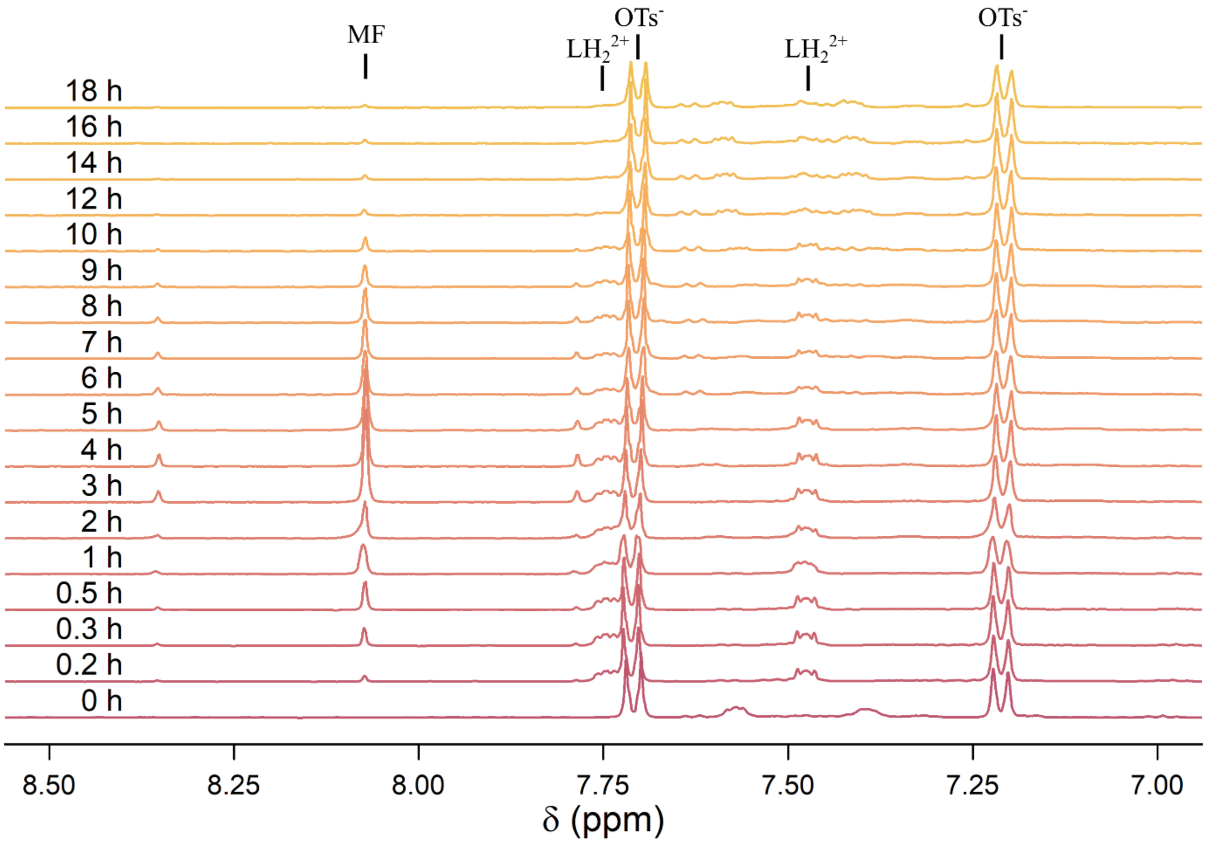

Figure S4. Ex-situ, background corrected ^{1}H-NMR spectra recorded at 25 °C of the hydroesterification of 1-octene with ^{13}C-PFA and [Pd(d^{t}bpx)] in d^{4}-MeOH. Field of view from 7.0 to 8.5 ppm. The methyl formate signal at 8.11 ppm shows strong ^{13}C satellites proofing that both methanol as well as ^{13}C-PFA are transformed into methyl formate. Legend: **MF** = HCOOMe, **LH_2^{2+}** = [2H-d^{t}bpx]$^{2+}$.

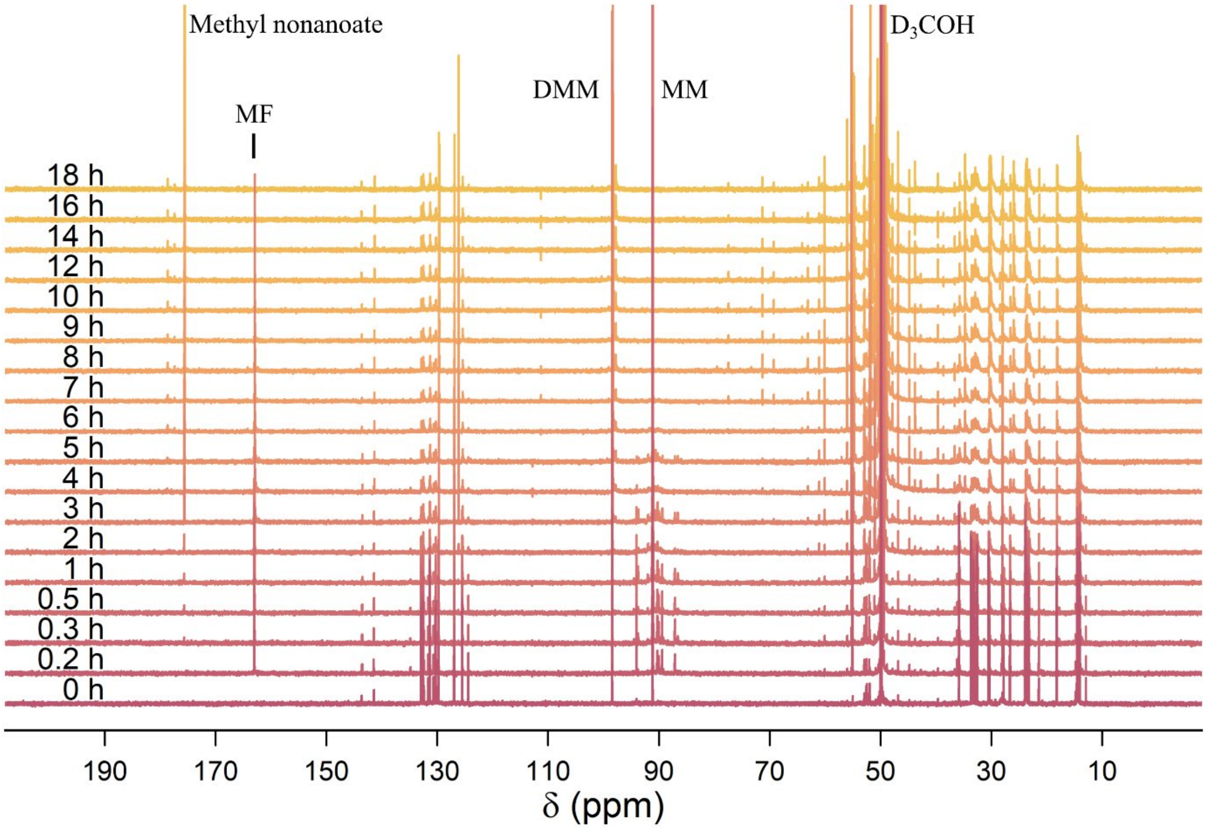

Figure S5. Ex-situ, background corrected ^{13}C-NMR spectra recorded at 25 °C of the hydroesterification of 1-octene with ^{13}C-PFA and [Pd(d^{t}bpx)] in d^4-MeOH. Field of view from 0 to 200 ppm. Legend: **MM** = $H_2C(OMe)(OH)$, **DMM** = $H_2C(OMe)_2$, **MF** = HCOOMe.

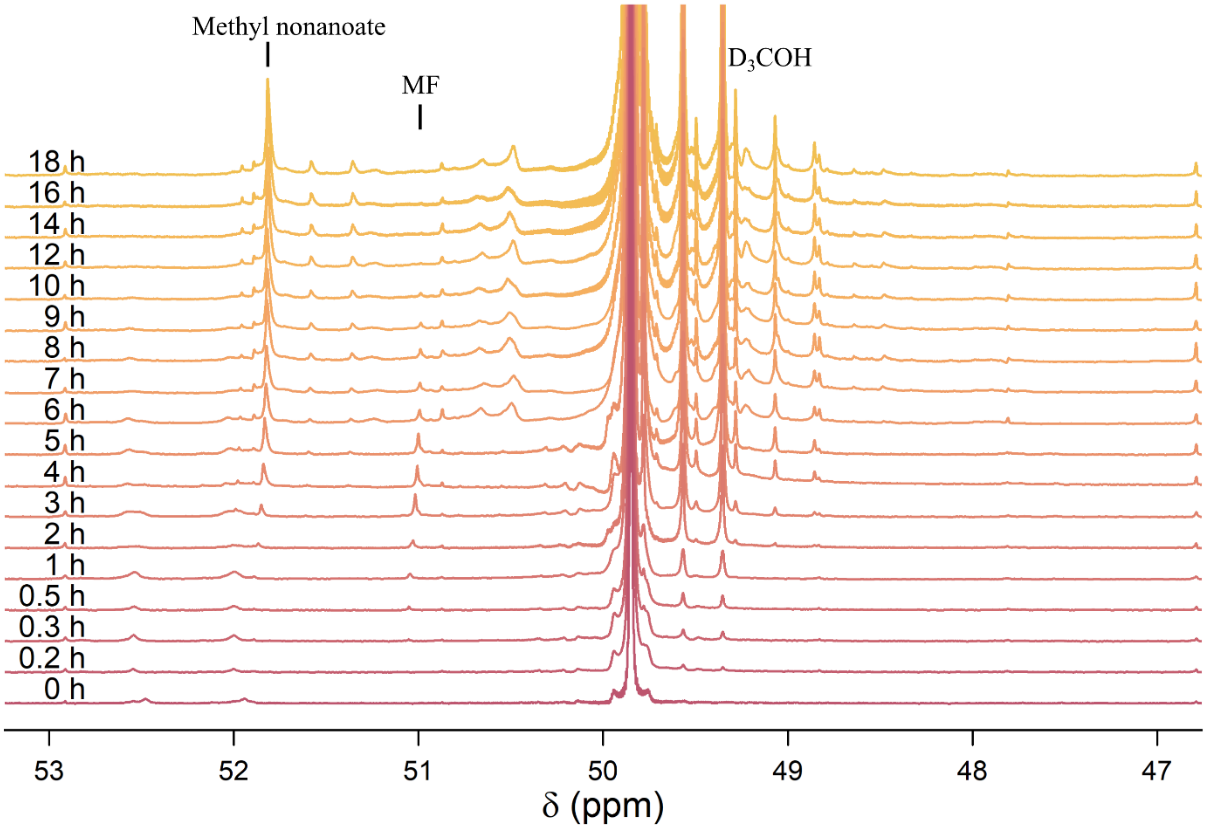

Figure S6. Ex-situ, background corrected ^{13}C-NMR spectra recorded at 25 °C of the hydroesterification of 1-octene with ^{13}C-PFA and [Pd(d^{t}bpx)] in d^4-MeOH. Field of view from 47 to 53 ppm. The ^1J(C-D) coupling patterns of CH_2DOH, CHD_2OH and CD_3OH are visible. Legend: **MF** = HCOOMe.

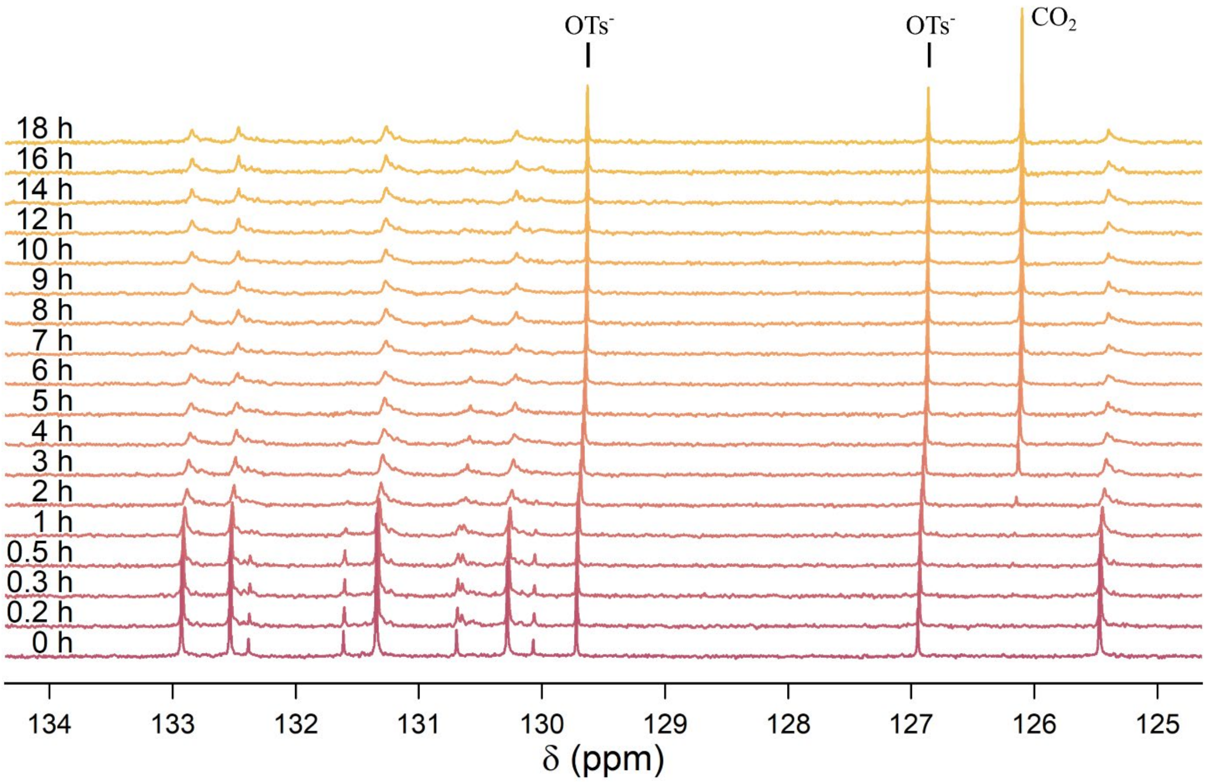

Figure S7. Ex-situ, background corrected ^{13}C-NMR spectra recorded at 25 °C of the hydroesterification of 1-octene with ^{13}C-PFA and [Pd(d^{t}bpx)] in d^4-MeOH. Field of view from 125 to 134 ppm.

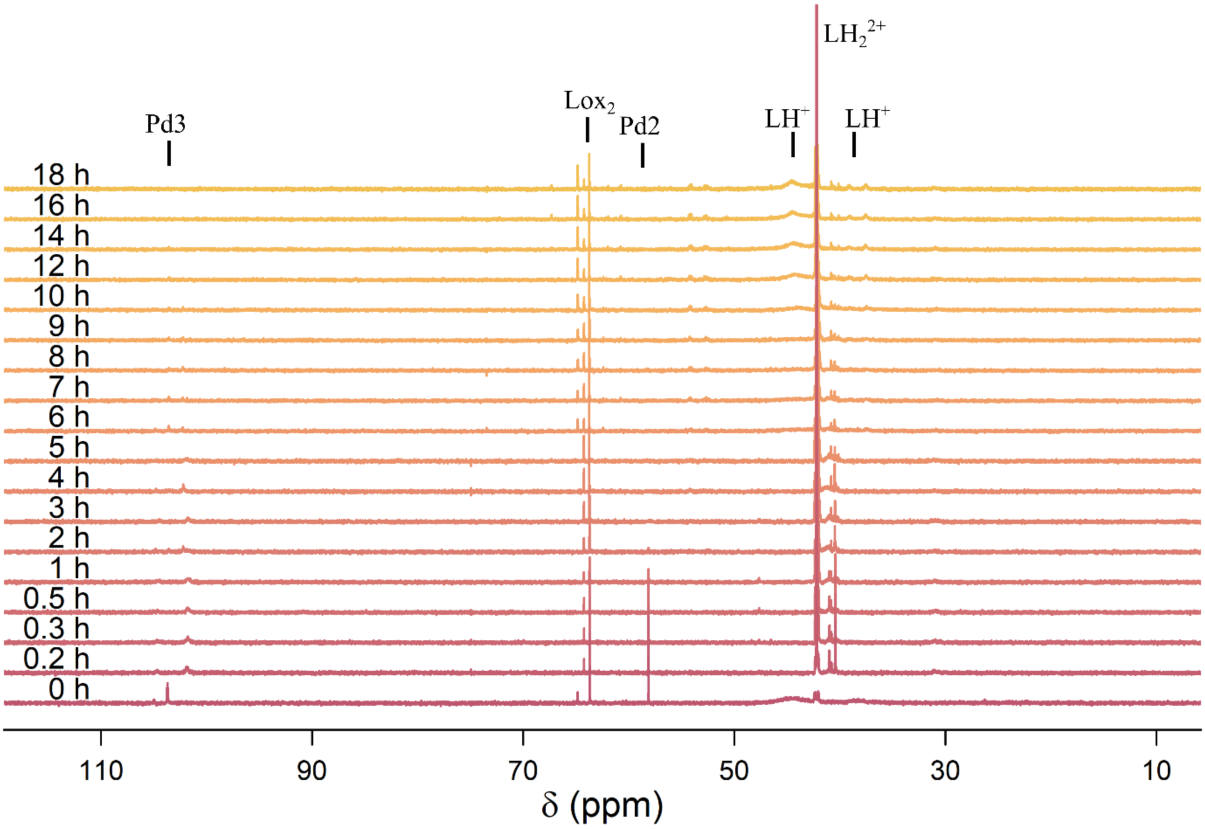

Figure S8. Ex-situ, background corrected ^{31}P-NMR spectra recorded at 25 °C of the hydroesterification of 1-octene with ^{13}C-PFA and [Pd(d^{t}bpx)] in d^4-MeOH. Field of view from 25 to 120 ppm. Legend: **LH^+** = [H-d^{t}bpx]$^+$, **LH_2^{2+}** = [2H-d^{t}bpx]$^{2+}$, **Lox_2** = *o*-$C_6H_4(CH_2P(O)(^tBu)_3)_2$, **Pd2** = [Pd(d^{t}bpx)(MeOH)$_2$]$^{2+}$, **Pd3** = [Pd(η^2-CP-d^{t}bpx-H)(η^2-OTs)][OTs].

HCOOMe

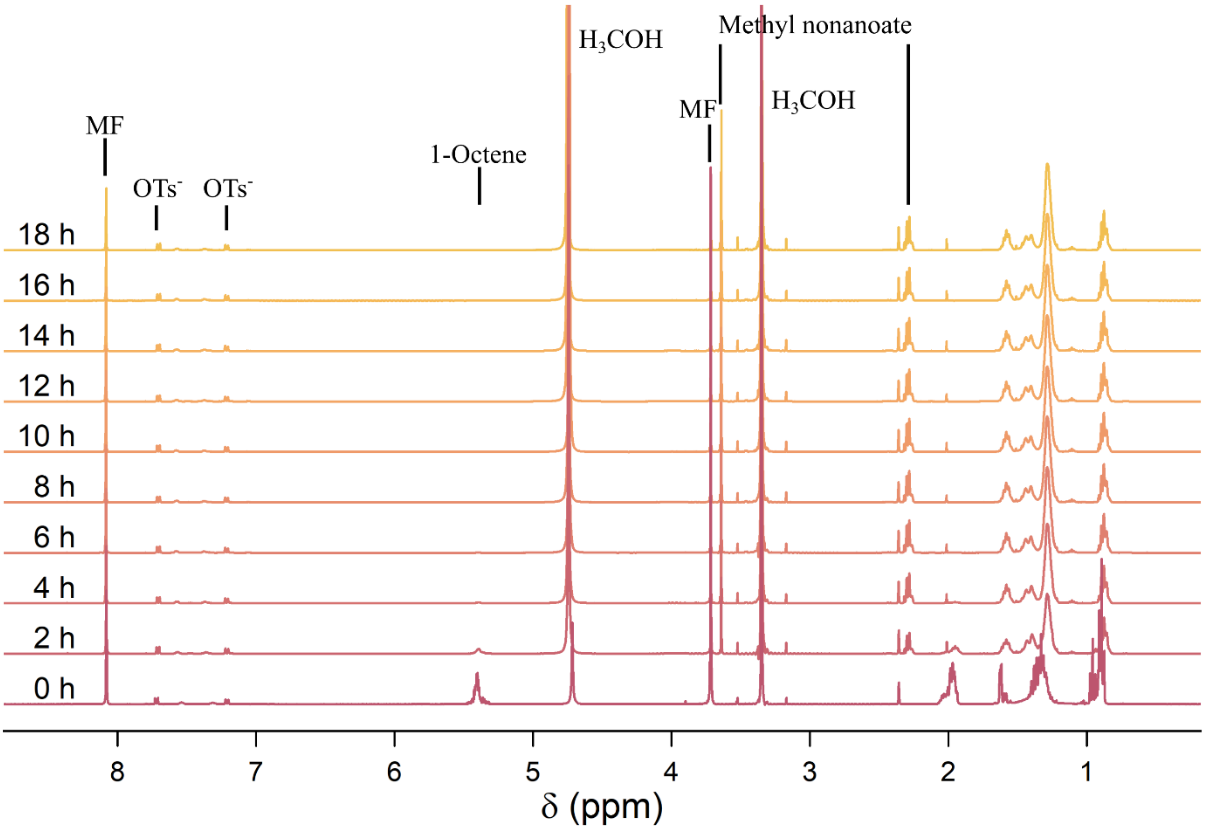

Figure S9. Ex-situ, background corrected ^{1}H-NMR spectra recorded at 25 °C of the hydroesterification of 1-octene with HCOOMe and [Pd(d^{t}bpx)] in d^{4}-MeOH. Field of view from 0.5 to 8.5 ppm. Legend: **MF** = HCOOMe.

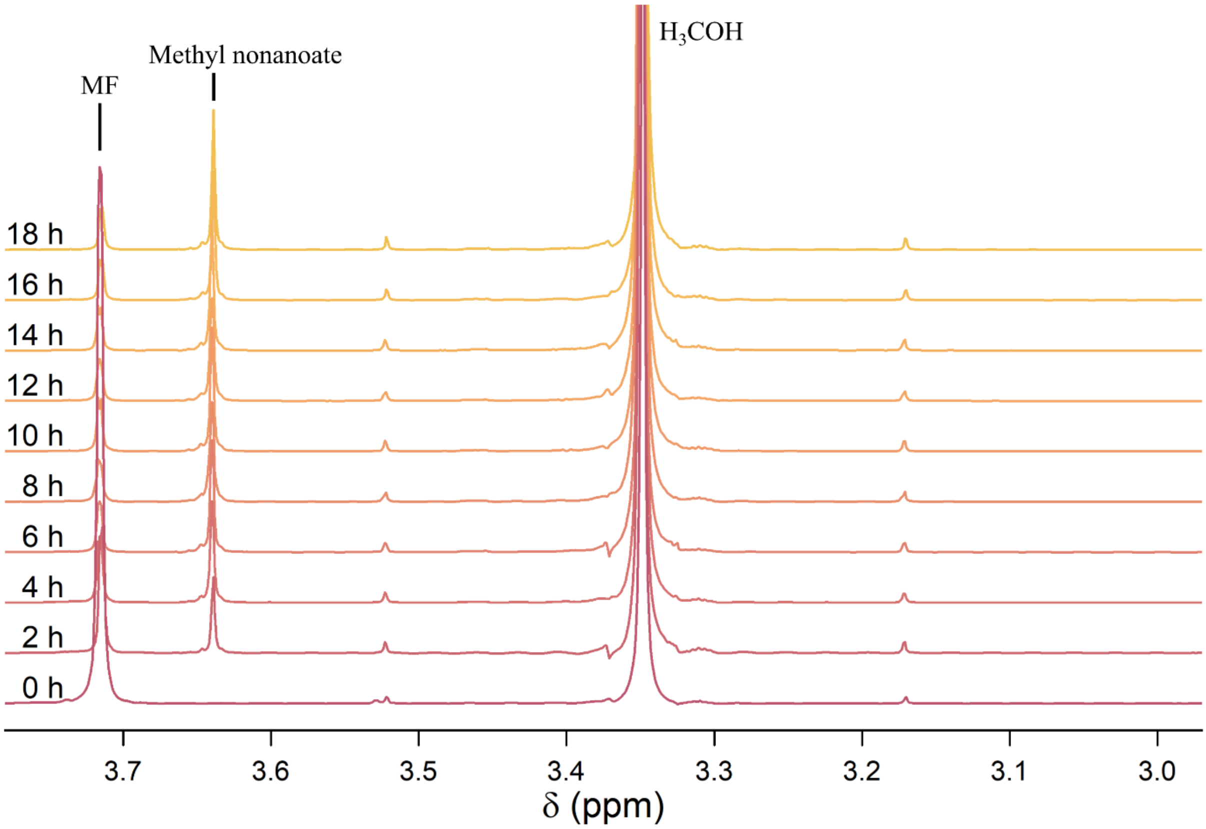

Figure S10. Ex-situ, background corrected ^{1}H-NMR spectra recorded at 25 °C of the hydroesterification of 1-octene with HCOOMe and [Pd(d^{t}bpx)] in d^4-MeOH. Field of view from 3.0 to 3.75 ppm. Legend: **MF** = HCOOMe.

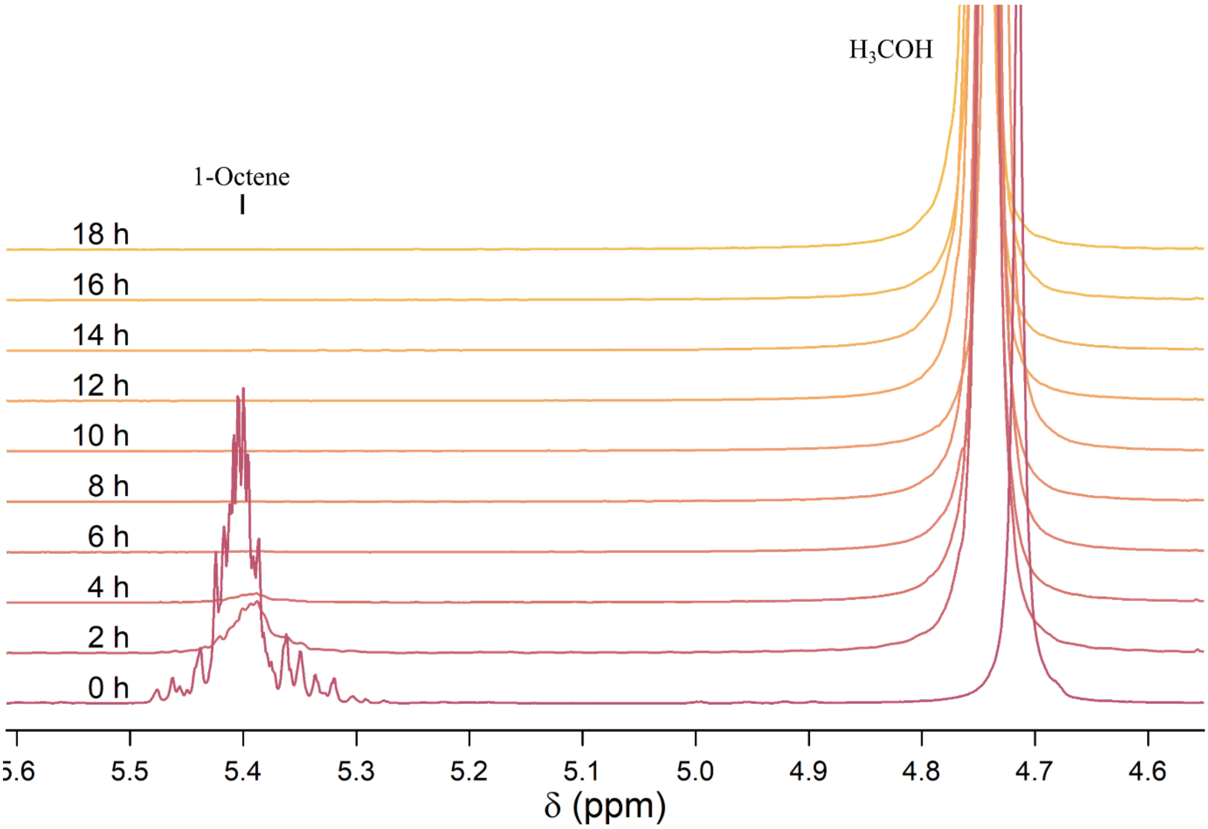

Figure S11. Ex-situ, background corrected ^{1}H-NMR spectra recorded at 25 °C of the hydroesterification of 1-octene with HCOOMe and [Pd(d'bpx)] in d^4-MeOH. Field of view from 4.5 to 5.6 ppm.

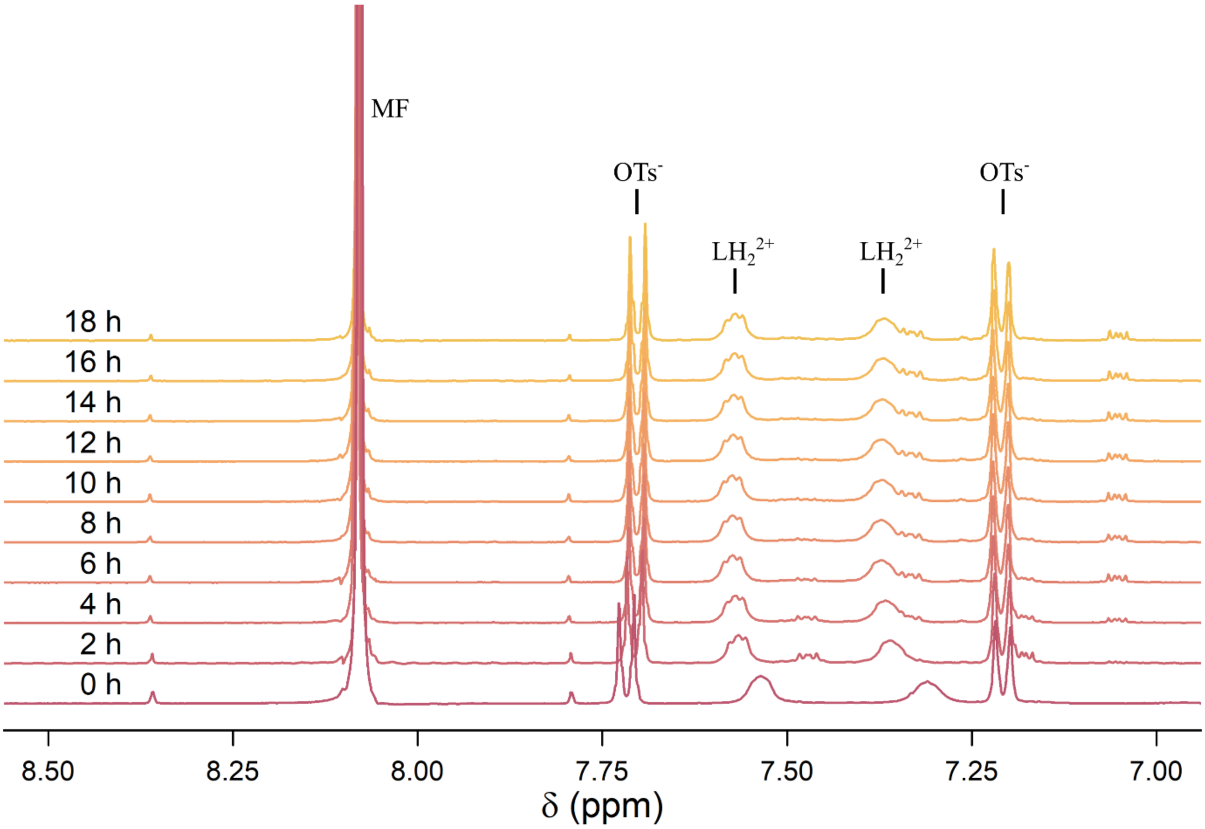

Figure S12. Ex-situ, background corrected ^{1}H-NMR spectra recorded at 25 °C of the hydroesterification of 1-octene with HCOOMe and [Pd(d^{t}bpx)] in d^{4}-MeOH. Field of view from 7.0 to 8.5 ppm. Legend: **MF** = HCOOMe, **LH_2^{2+}** = [2H-d^{t}bpx]$^{2+}$.

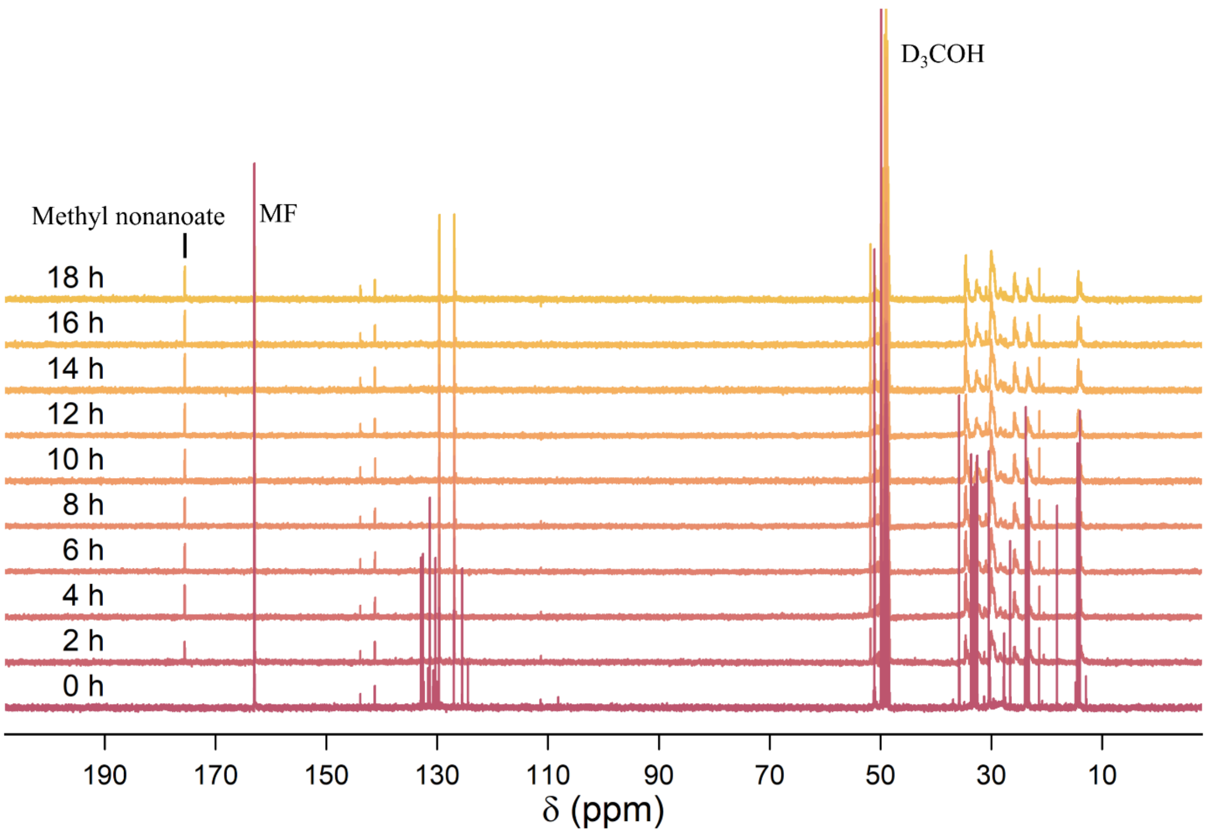

Figure S13. Ex-situ, background corrected ^{13}C-NMR spectra recorded at 25 °C of the hydroesterification of 1-octene with HCOOMe and [Pd(d^{t}bpx)] in d^4-MeOH. Field of view from 0 to 200 ppm. Legend: **MF** = HCOOMe.

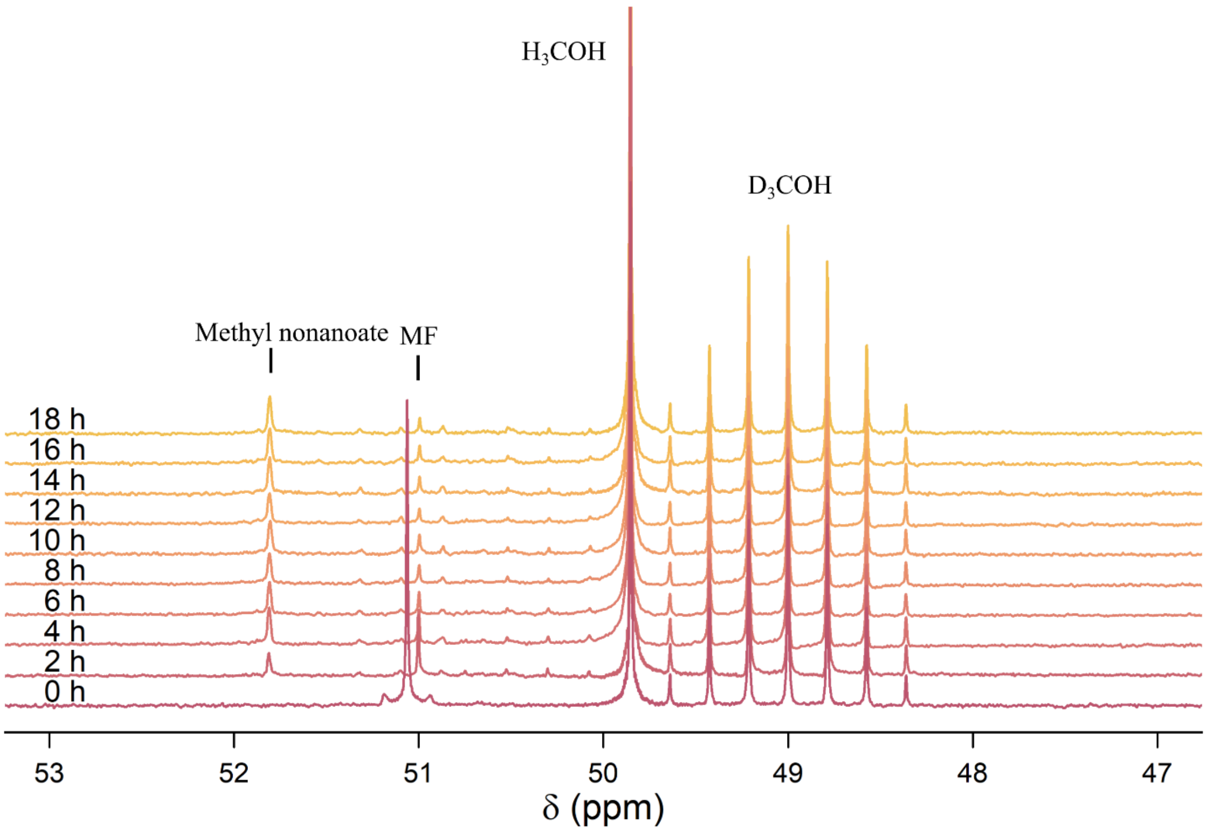

Figure S14. Ex-situ, background corrected ^{13}C-NMR spectra recorded at 25 °C of the hydroesterification of 1-octene with HCOOMe and [Pd(d^{t}bpx)] in d^4-MeOH. Field of view from 47 to 53 ppm. The CH_3 group of methyl nonanoate at 51.9 ppm does not show any ^1J(C-D) coupling patterns indicating that the CH_3 group stems exclusively from non-deuterated methyl formate. This further excludes CO as the carbonylation agent. Legend: **MF** = HCOOMe.

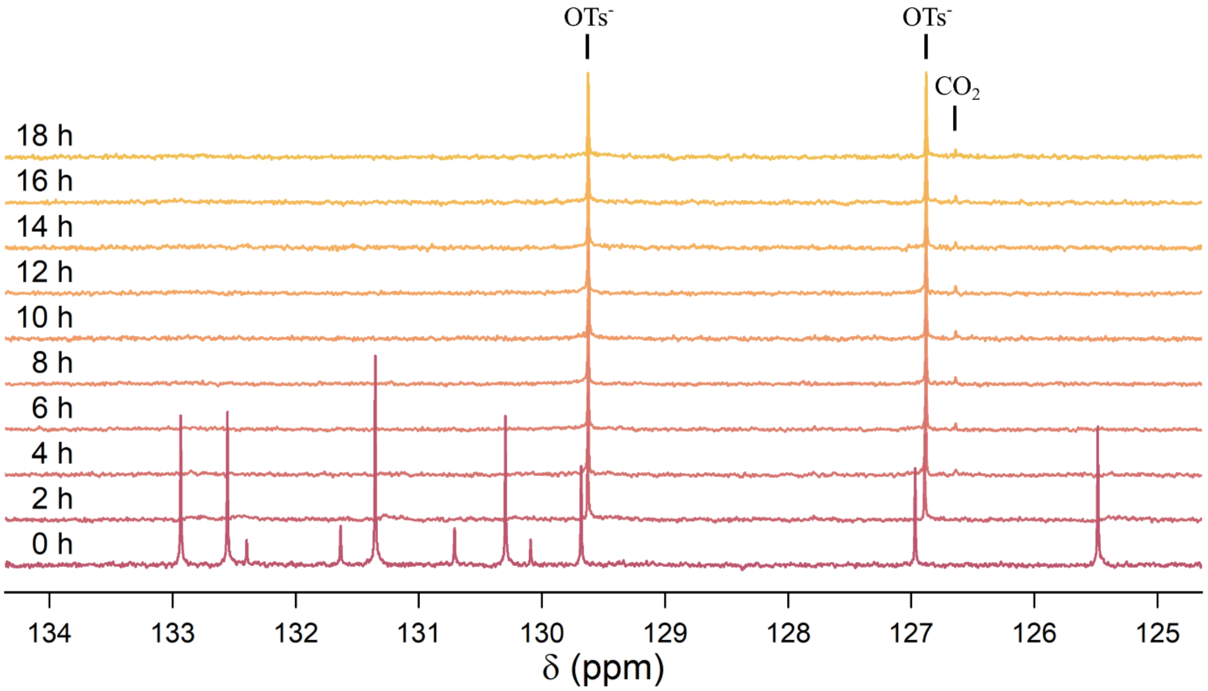

Figure S15. Ex-situ, background corrected ^{13}C-NMR spectra recorded at 25 °C of the hydroesterification of 1-octene with HCOOMe and [Pd(d^{t}bpx)] in d^{4}-MeOH. Field of view from 125 to 134 ppm.

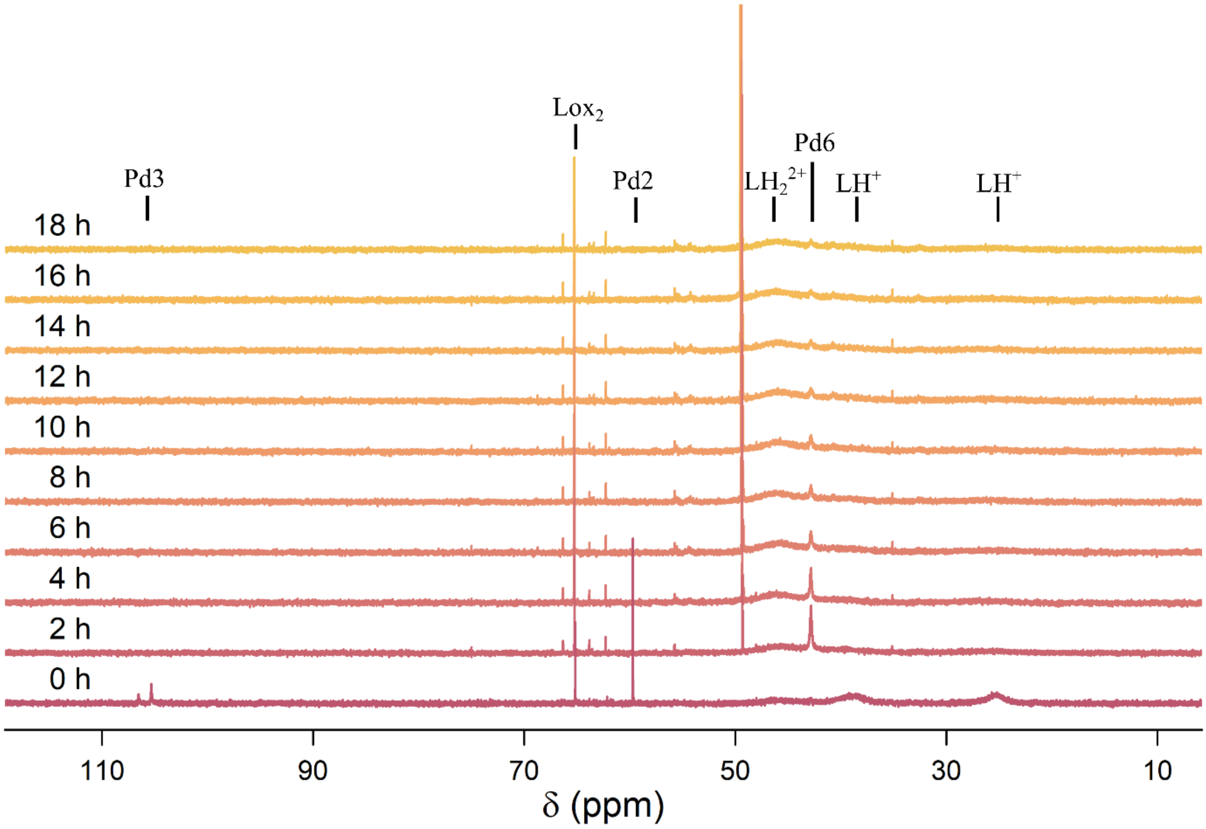

Figure S16. Ex-situ, background corrected ^{31}P-NMR spectra recorded at 25 °C of the hydroesterification of 1-octene with HCOOMe and [Pd(d^{t}bpx)] in d^4-MeOH. Field of view from 10 to 120 ppm. Legend: **LH$^+$** = [H-d^{t}bpx]$^+$, **LH$_2^{2+}$** = [2H-d^{t}bpx]$^{2+}$, **Lox$_2$** = o-$C_6H_4(CH_2P(O)(^tBu)_3)_2$, **Pd2** = [Pd(d^{t}bpx)(MeOH)$_2$]$^{2+}$, **Pd3** = [Pd(η^2-CP-d^{t}bpx-H)(η^2-OTs)][OTs], **Pd6** = [Pd(d^{t}bpx)(μ-H)]$_2$.

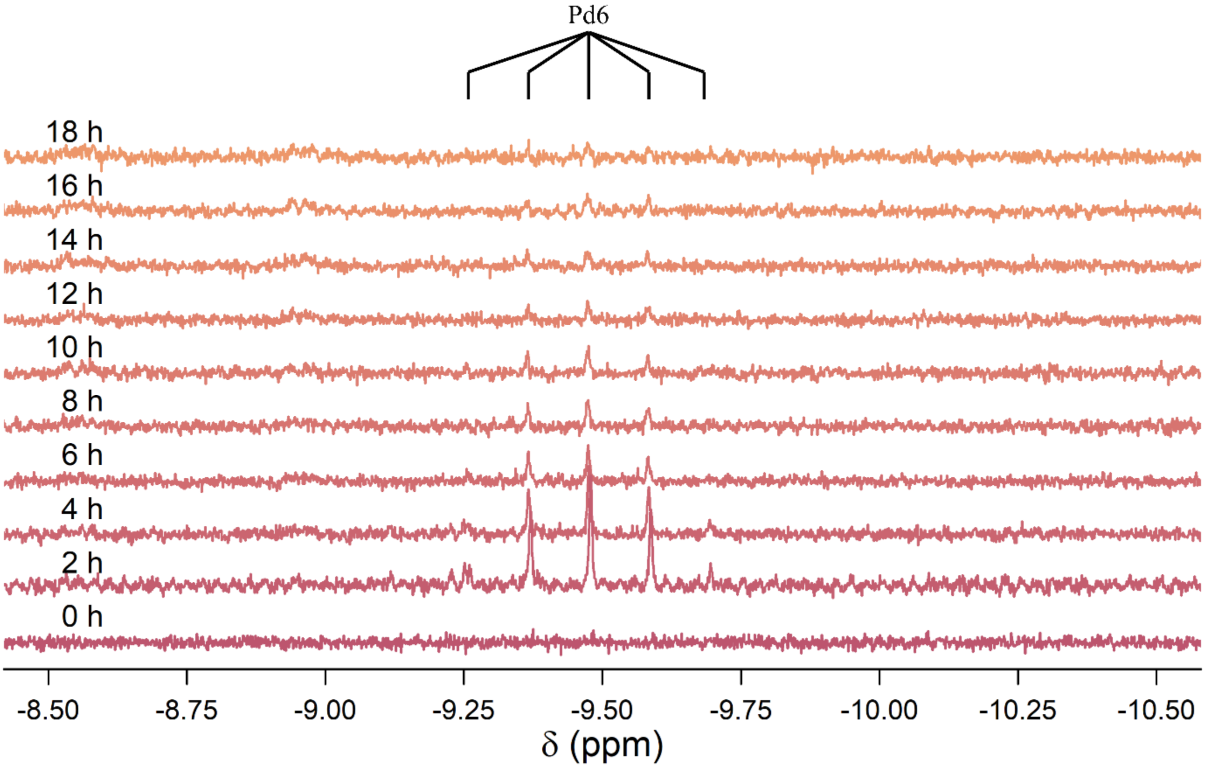

Figure S17. Ex-situ, background corrected ^{1}H-NMR spectra recorded at 25 °C of the hydroesterification of 1-octene with HCOOMe and [Pd(d^{t}bpx)] in d^{4}-MeOH. Field of view from -10.5 to -8.5 ppm. The quintet hydride signal of [Pd(d^{t}bpx)(μ-H)]$_2$ is visible. Legend: **Pd6** = [Pd(d^{t}bpx)(μ-H)]$_2$.

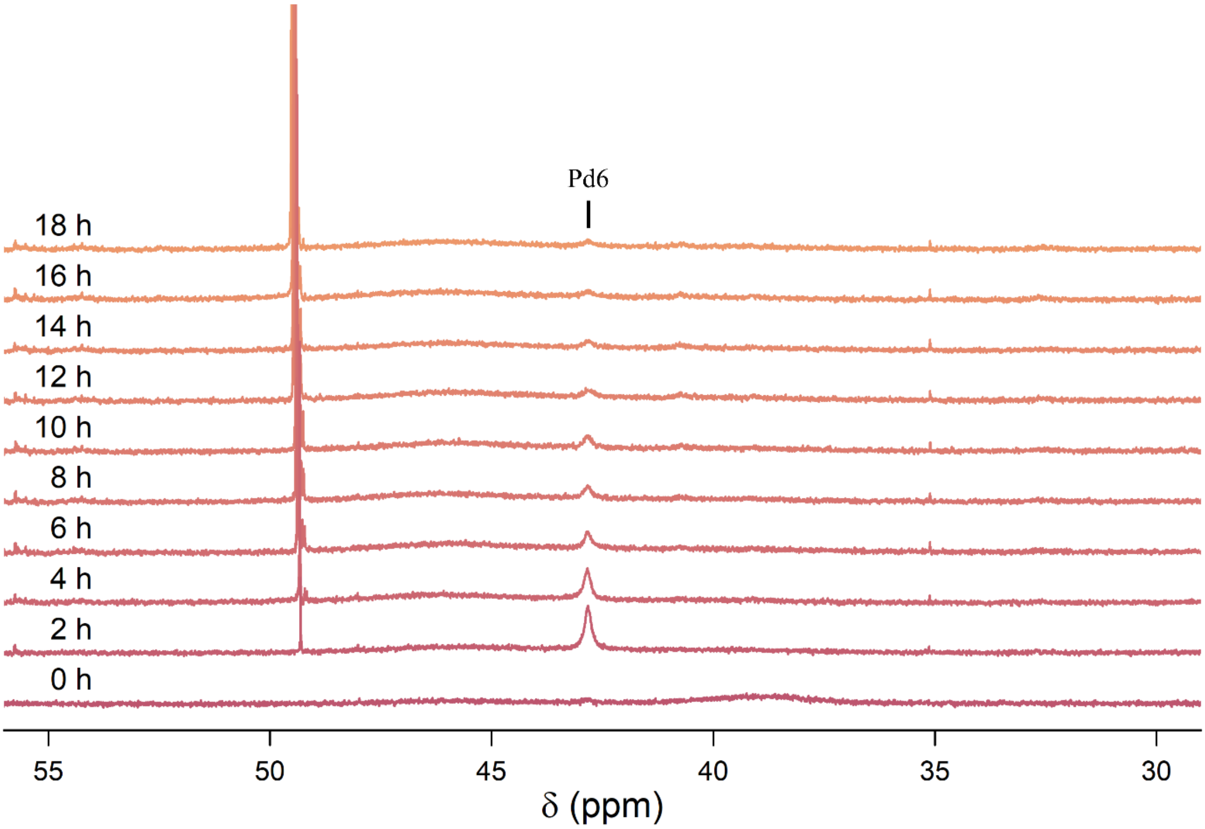

Figure S18. Ex-situ, background corrected ^{31}P-NMR spectra recorded at 25 °C of the hydroesterification of 1-octene with HCOOMe and [Pd(d^{t}bpx)] in d^4-MeOH. Field of view from 30 to 55 ppm. The signal of [Pd(d^{t}bpx)(μ-H)]$_2$ can be seen at 42.8 ppm which correlates with the hydride signal shown in Figure S17. Legend: **Pd6** = [Pd(d^{t}bpx)(μ-H)]$_2$.

HCOOH

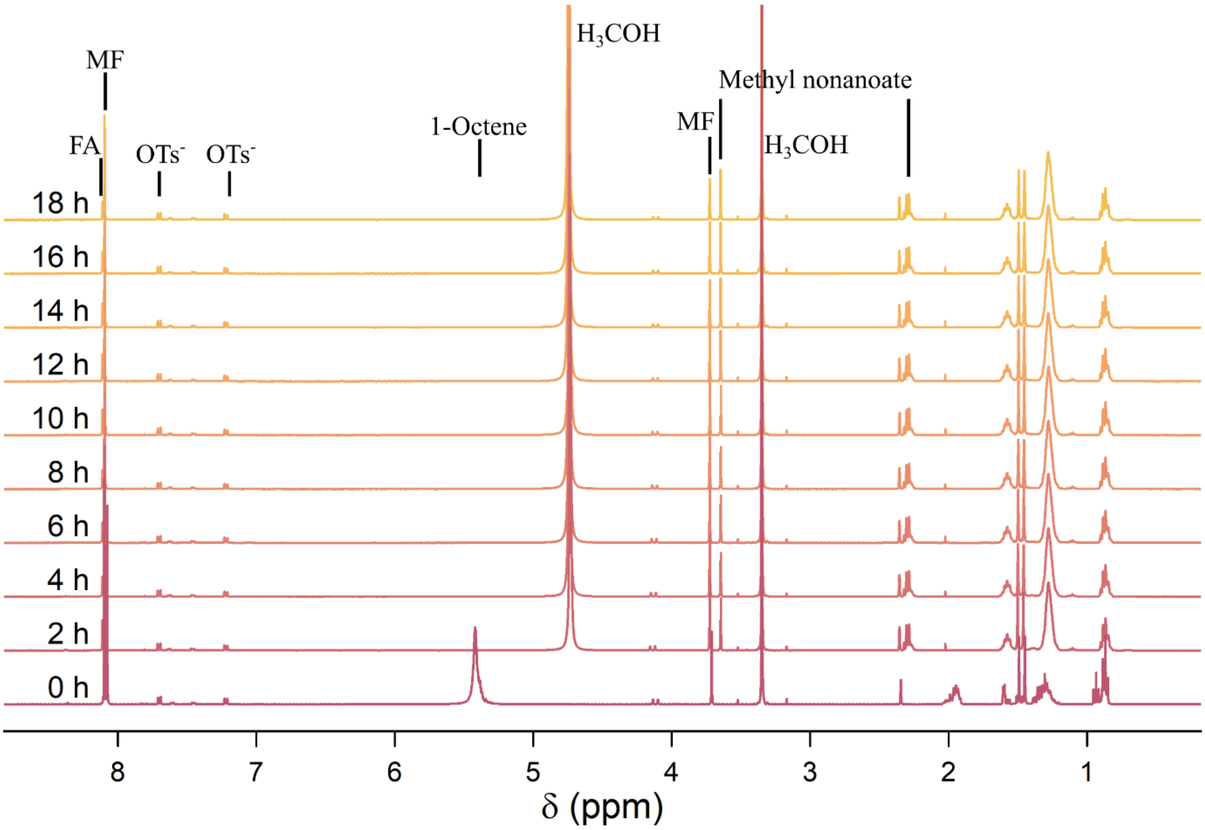

Figure S19. Ex-situ, background corrected ^{1}H-NMR spectra recorded at 25 °C of the hydroesterification of 1-octene with HCOOH and [Pd(d^{t}bpx)] in d[4]-MeOH. Field of view from 0.5 to 8.5 ppm. Legend: **MF** = HCOOMe, **FA** = HCOOH.

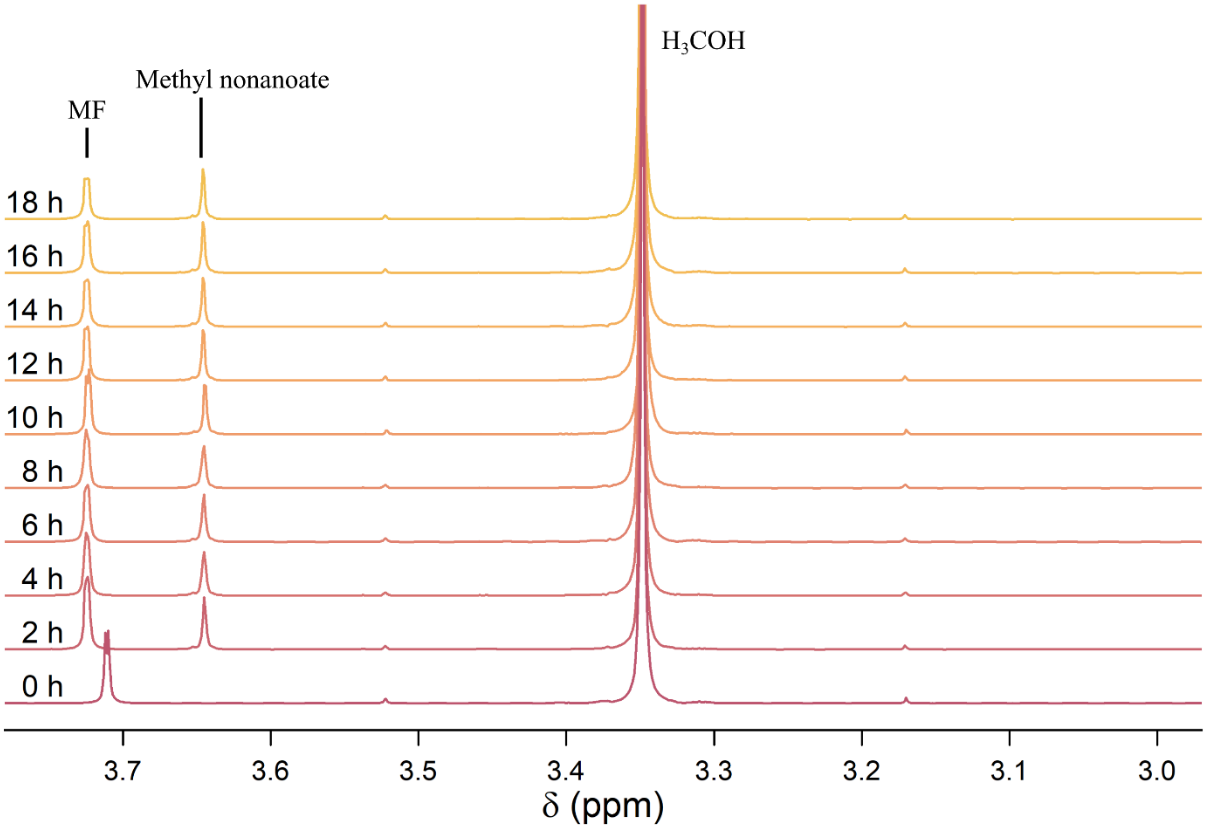

Figure S20. Ex-situ, background corrected ^{1}H-NMR spectra recorded at 25 °C of the hydroesterification of 1-octene with HCOOH and [Pd(d^{t}bpx)] in d[4]-MeOH. Field of view from 3.0 to 3.75 ppm. Legend: **MF** = HCOOMe.

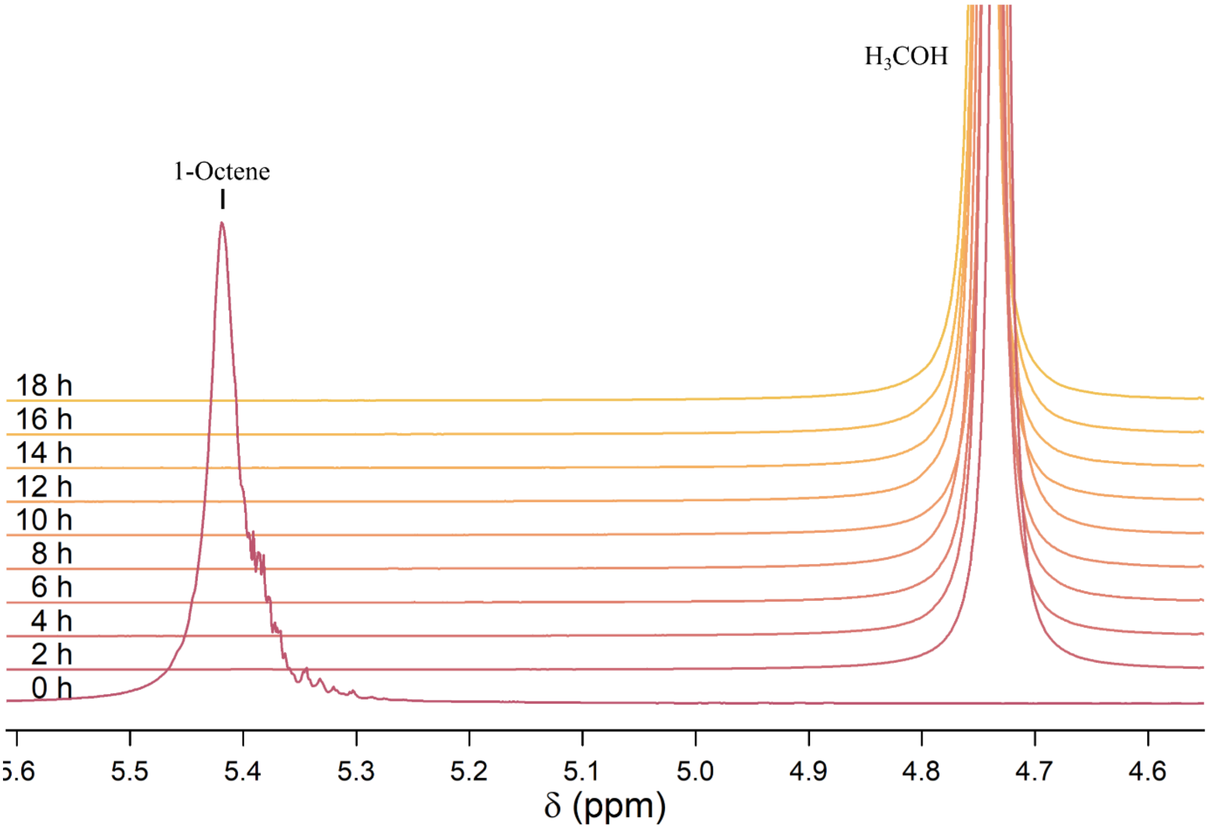

Figure S21. Ex-situ, background corrected ^{1}H-NMR spectra recorded at 25 °C of the hydroesterification of 1-octene with HCOOH and [Pd(d^{t}bpx)] in d^4-MeOH. Field of view from 4.5 to 5.6 ppm.

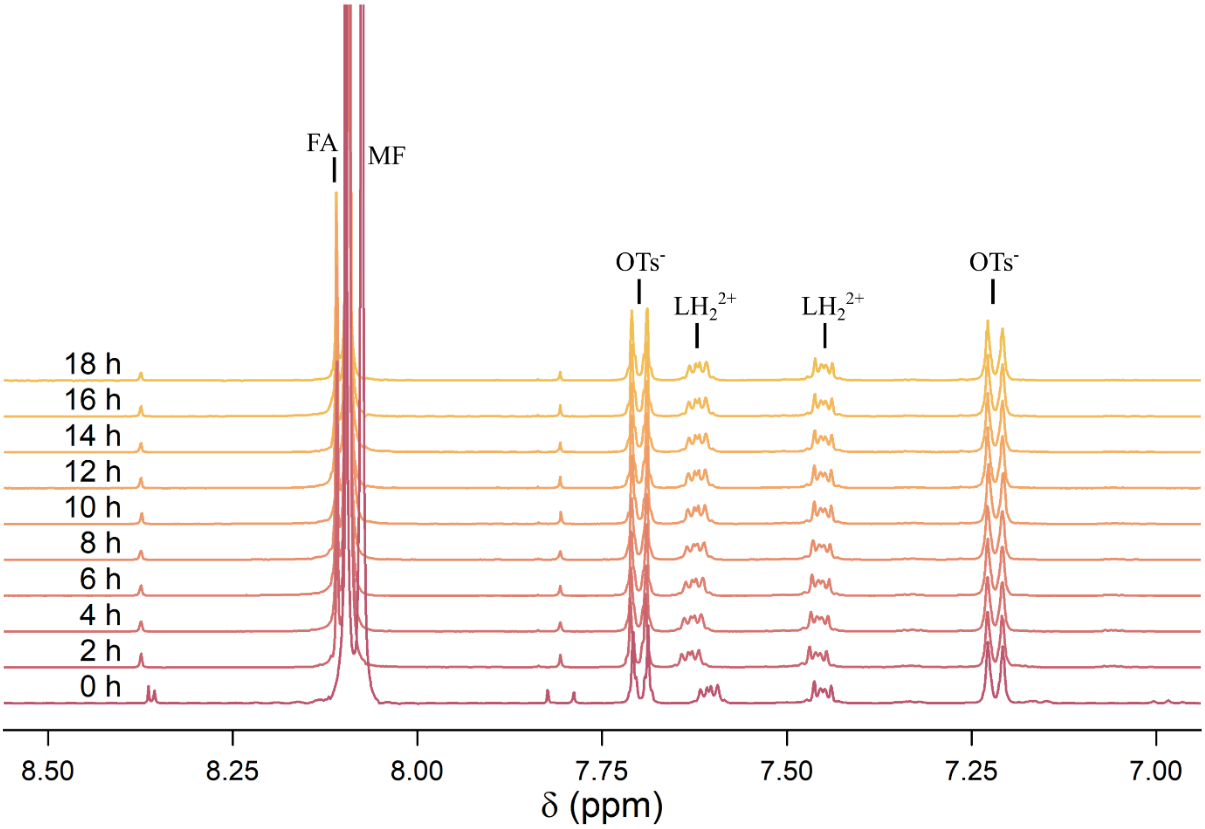

Figure S22. Ex-situ, background corrected ^{1}H-NMR spectra recorded at 25 °C of the hydroesterification of 1-octene with HCOOH and [Pd(d^{t}bpx)] in d^{4}-MeOH. Field of view from 7.0 to 8.5 ppm. Legend: **MF** = HCOOMe, **FA** = HCOOH, **LH_2^{2+}** = [2H-d^{t}bpx]$^{2+}$.

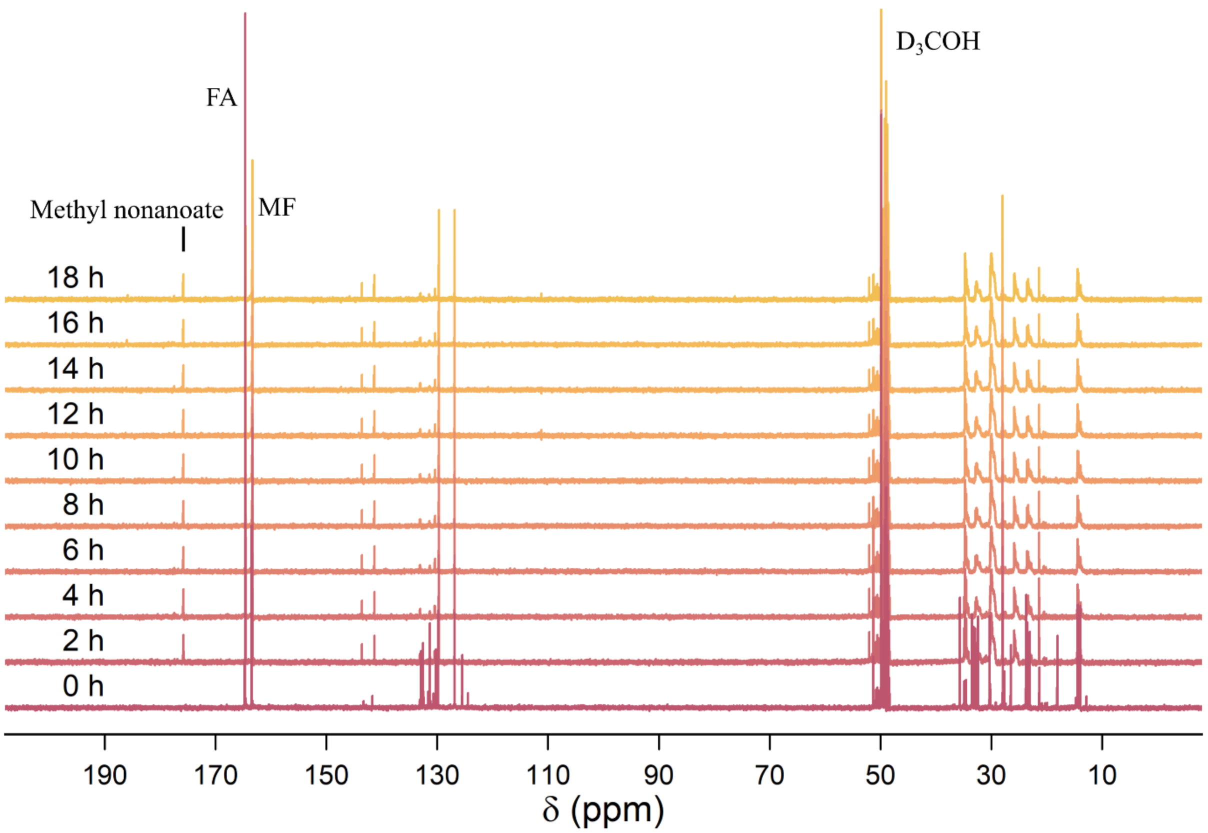

Figure S23. Ex-situ, background corrected ^{13}C-NMR spectra recorded at 25 °C of the hydroesterification of 1-octene with HCOOH and [Pd(d^{t}bpx)] in d^4-MeOH. Field of view from 0 to 200 ppm. Legend: **MF** = HCOOMe, **FA** = HCOOH.

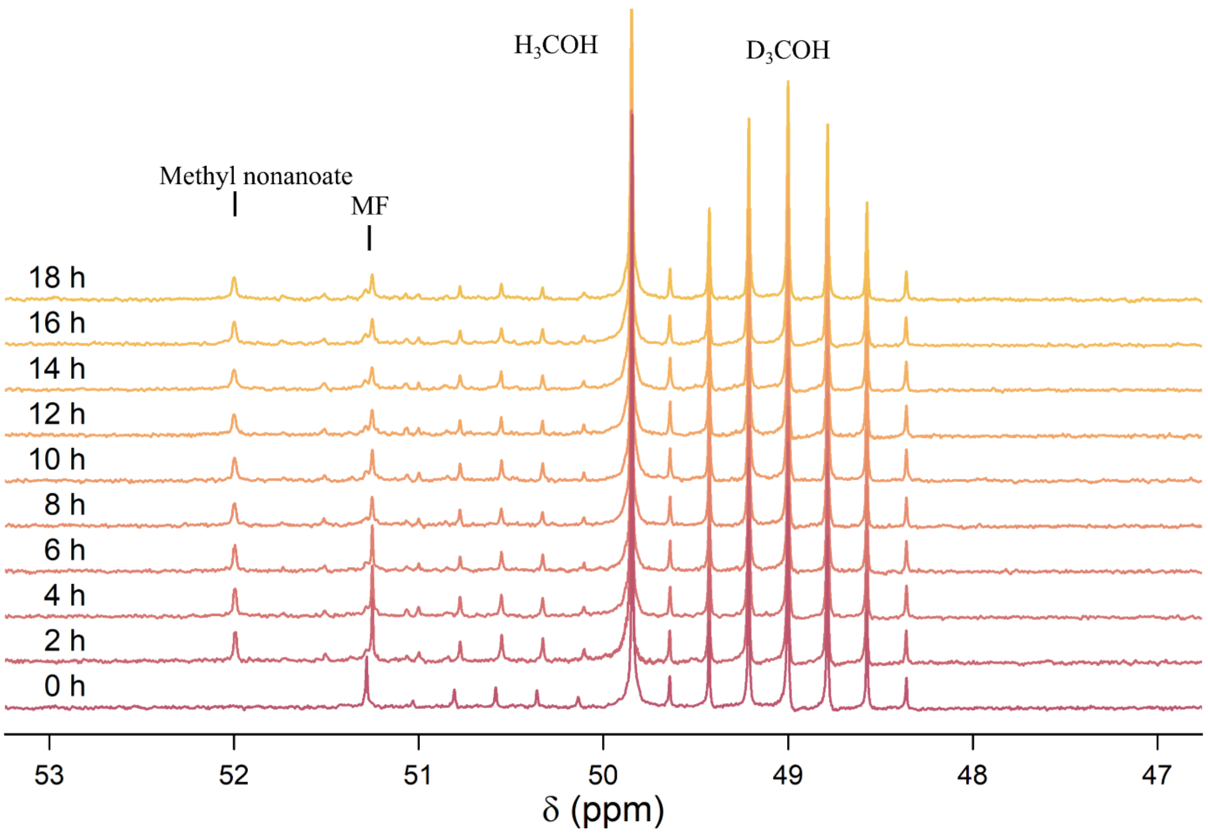

Figure S24. Ex-situ, background corrected ^{13}C-NMR spectra recorded at 25 °C of the hydroesterification of 1-octene with HCOOH and [Pd(d^{t}bpx)] in d^4-MeOH. Field of view from 47 to 53 ppm. Legend: **MF** = HCOOMe.

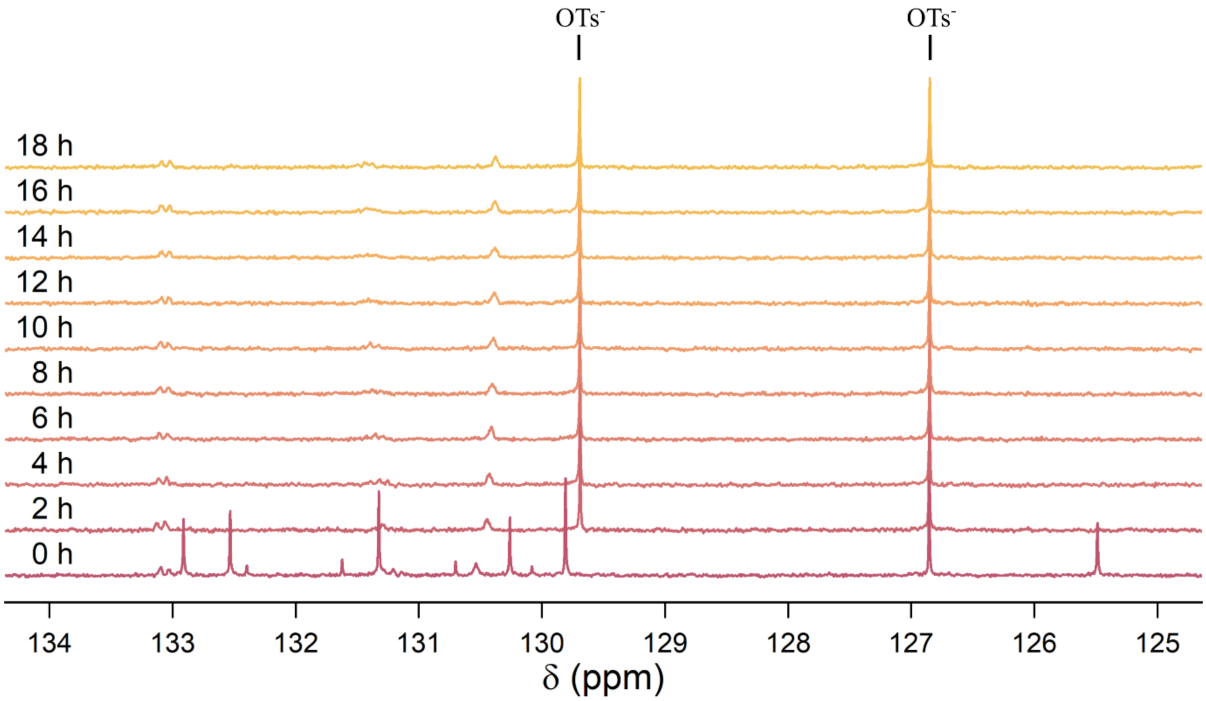

Figure S25. Ex-situ, background corrected ^{13}C-NMR spectra recorded at 25 °C of the hydroesterification of 1-octene with HCOOH and [Pd(d^{t}bpx)] in d^4-MeOH. Field of view from 125 to 134 ppm.

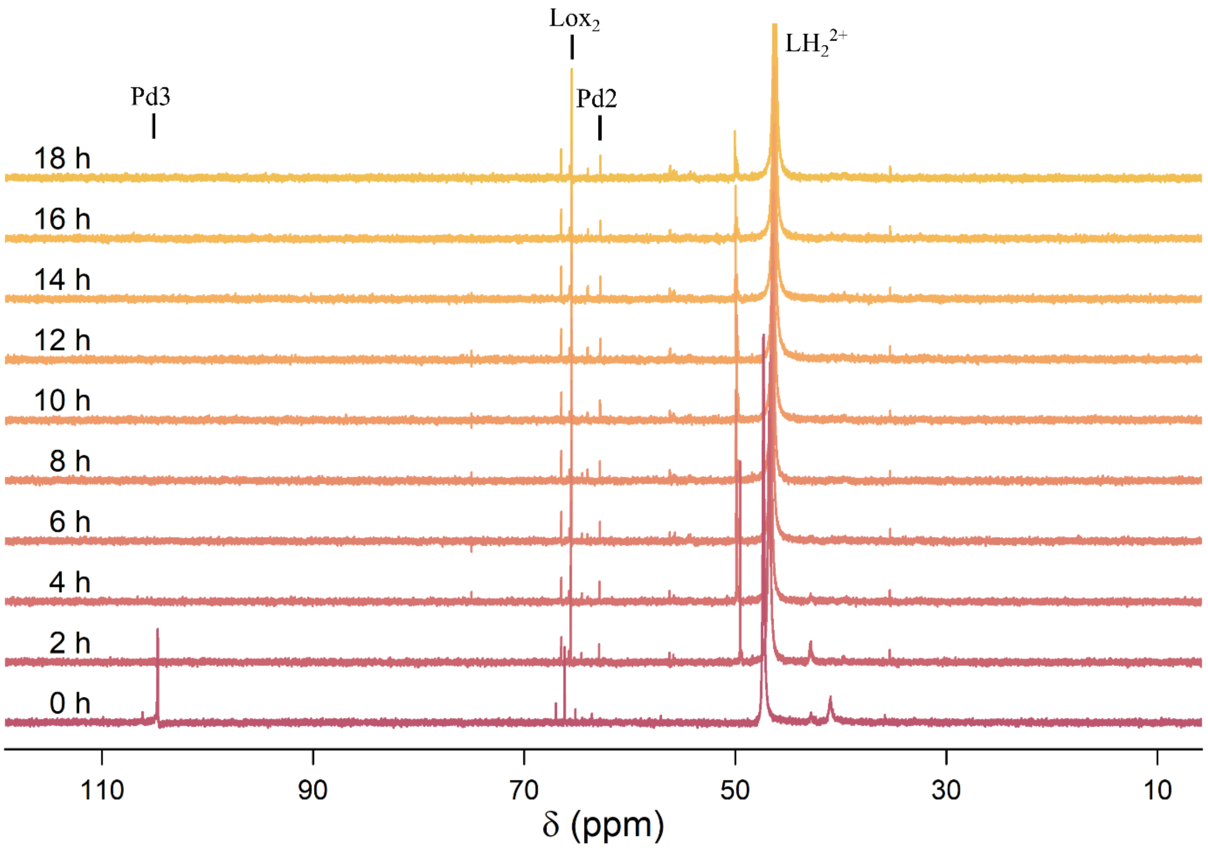

Figure S26. Ex-situ, background corrected ^{31}P-NMR spectra recorded at 25 °C of the hydroesterification of 1-octene with HCOOH and [Pd(d^{t}bpx)] in d^4-MeOH. Field of view from 10 to 120 ppm. Legend: **LH_2^{2+}** = [2H-d^{t}bpx]$^{2+}$, **Lox_2** = o-$C_6H_4(CH_2P(O)(^tBu)_3)_2$, **Pd2** = [Pd(d^{t}bpx)(MeOH)$_2$]$^{2+}$, **Pd3** = [Pd(η^2-CP-d^{t}bpx-H)(η^2-OTs)][OTs].

HCOOPh

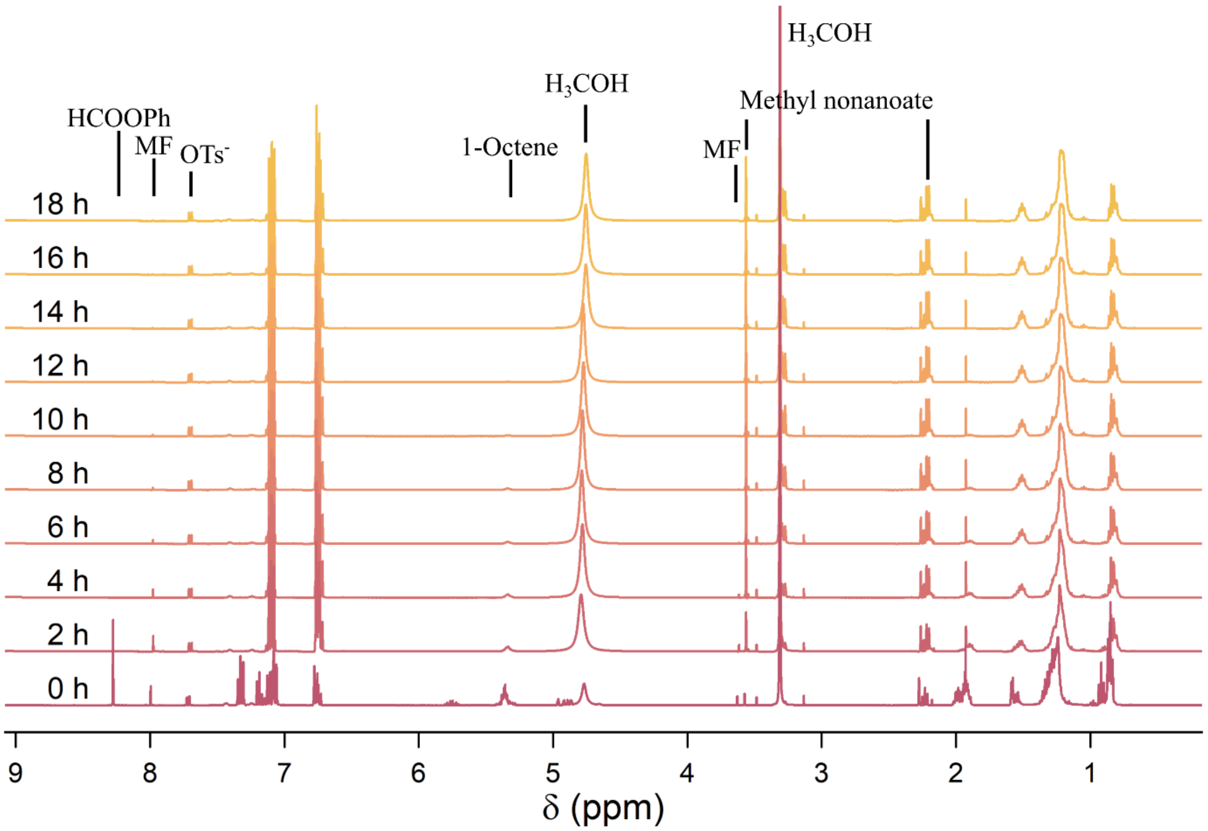

Figure S27. Ex-situ, background corrected ^{1}H-NMR spectra recorded at 25 °C of the hydroesterification of 1-octene with HCOOPh and [Pd(d^{t}bpx)] in d^{4}-MeOH. Field of view from 0.5 to 8.5 ppm. HCOOPh transforms rapidly into HCOOMe under acidic conditions and an excess of d^{4}-MeOH. Legend: **MF** = HCOOMe.

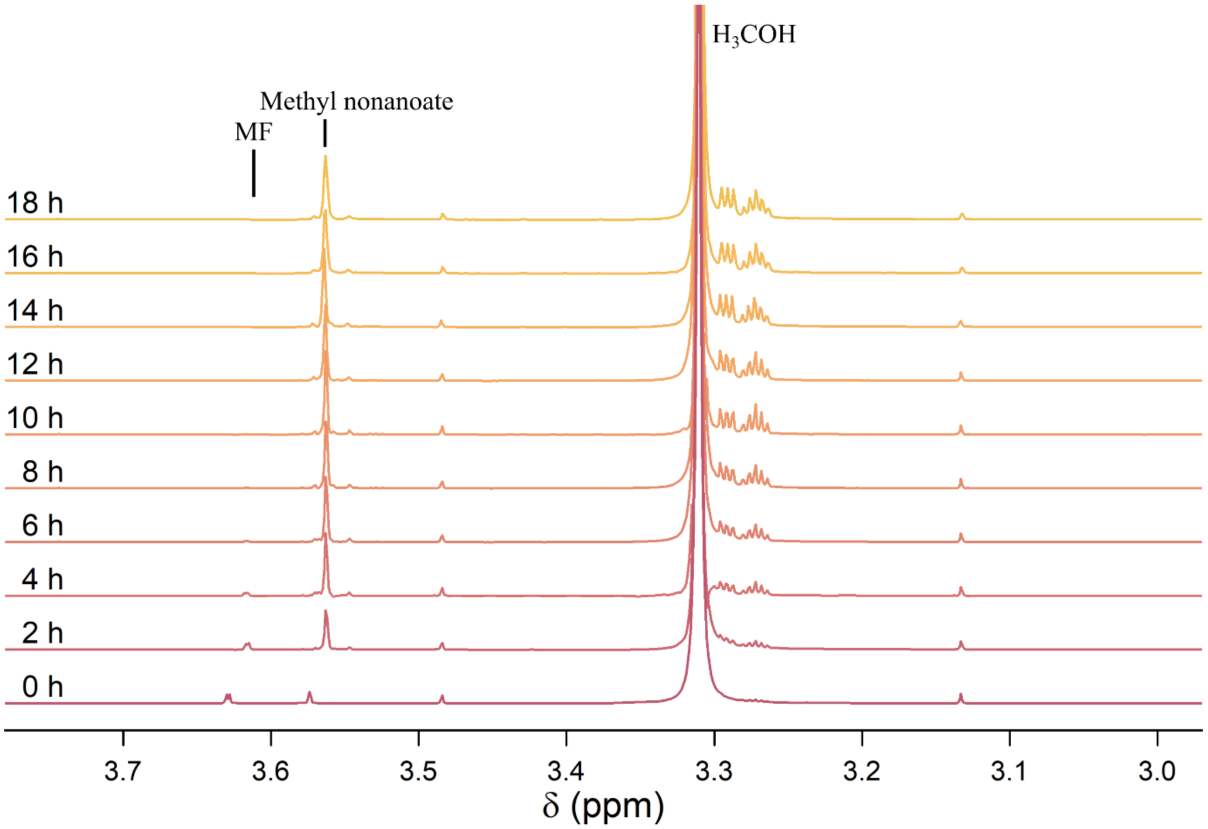

Figure S28. Ex-situ, background corrected ^{1}H-NMR spectra recorded at 25 °C of the hydroesterification of 1-octene with HCOOPh and [Pd(d^{t}bpx)] in d^{4}-MeOH. Field of view from 3.0 to 3.75 ppm. HCOOPh transforms rapidly into HCOOMe under acidic conditions and an excess of d^{4}-MeOH. Legend: **MF** = HCOOMe.

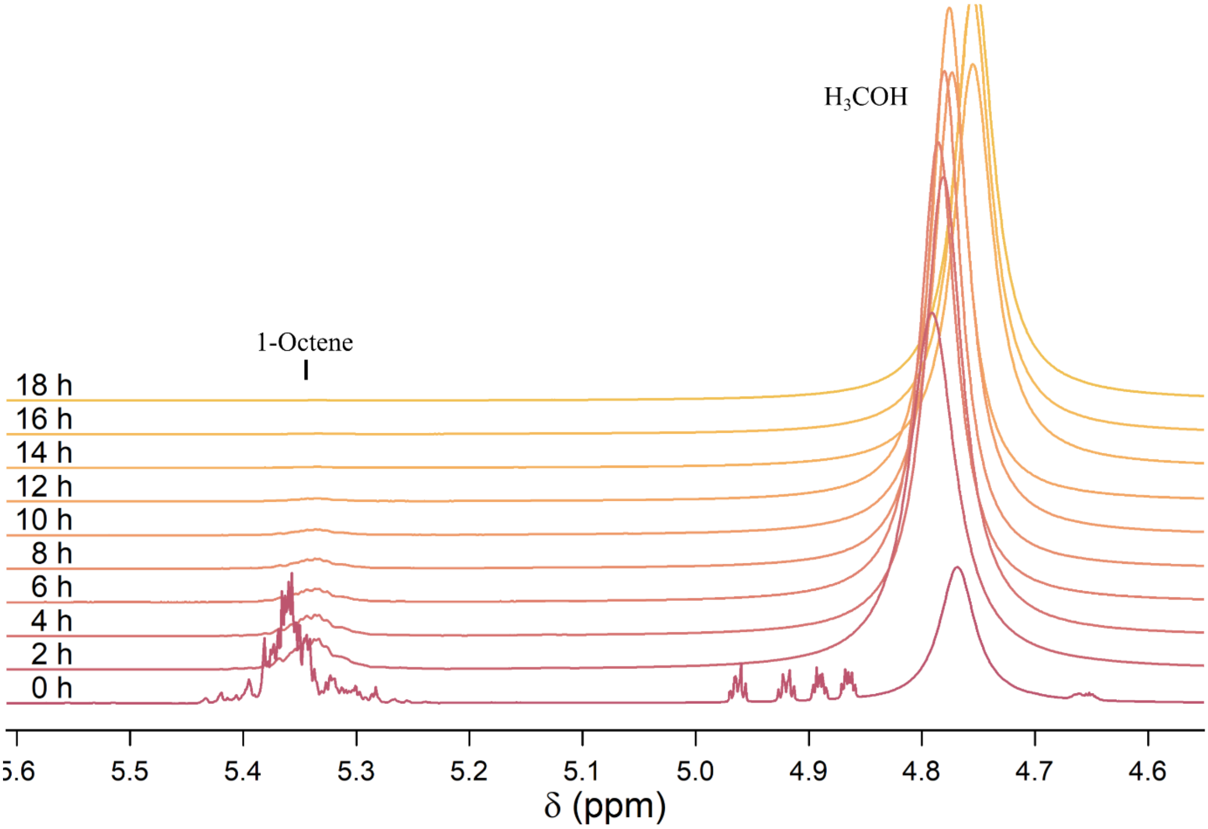

Figure S29. Ex-situ, background corrected ^{1}H-NMR spectra recorded at 25 °C of the hydroesterification of 1-octene with HCOOPh and [Pd(d^{t}bpx)] in d^{4}-MeOH. Field of view from 4.5 to 5.6 ppm. HCOOPh transforms rapidly into HCOOMe under acidic conditions and an excess of d^{4}-MeOH.

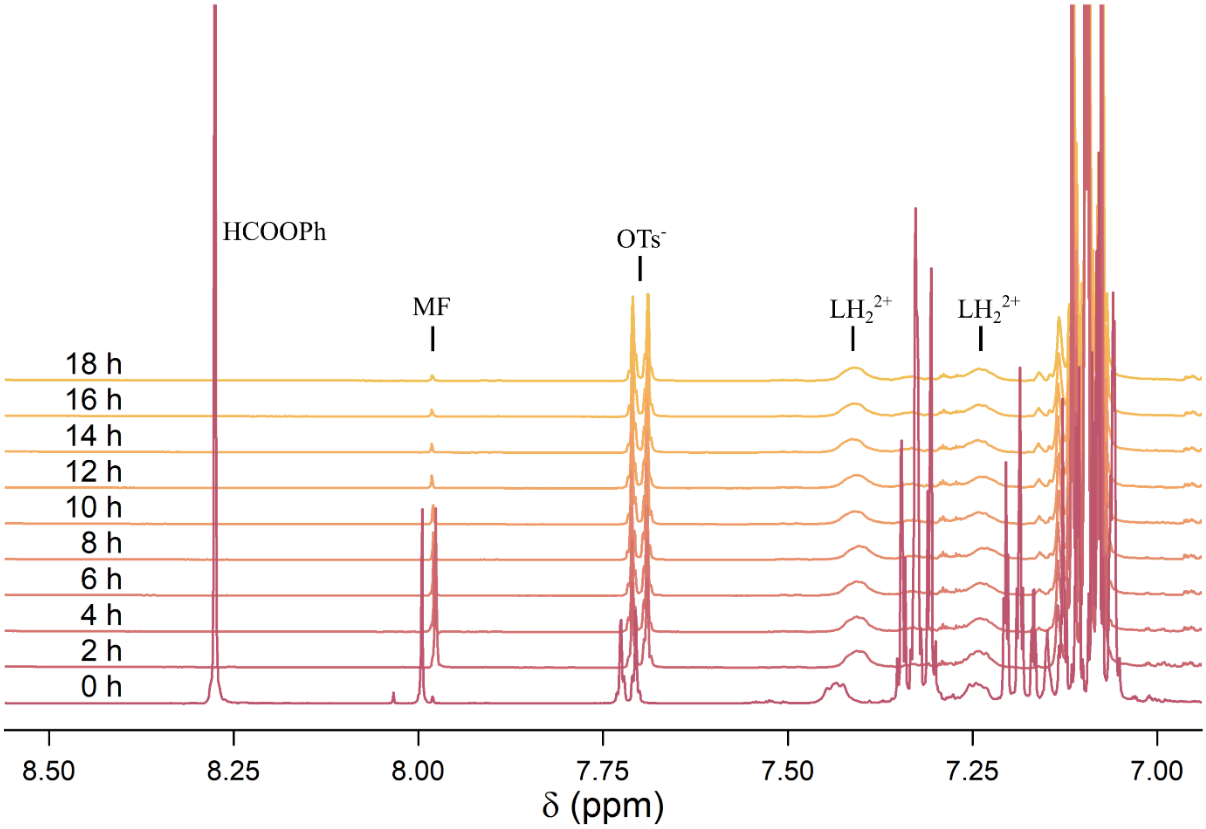

Figure S30. Ex-situ, background corrected ^{1}H-NMR spectra recorded at 25 °C of the hydroesterification of 1-octene with HCOOPh and [Pd(d^{t}bpx)] in d^{4}-MeOH. Field of view from 7.0 to 8.5 ppm. HCOOPh transforms rapidly into HCOOMe under acidic conditions and an excess of d^{4}-MeOH. Legend: **MF** = HCOOMe, **LH_2^{2+}** = [2H-d^{t}bpx]$^{2+}$.

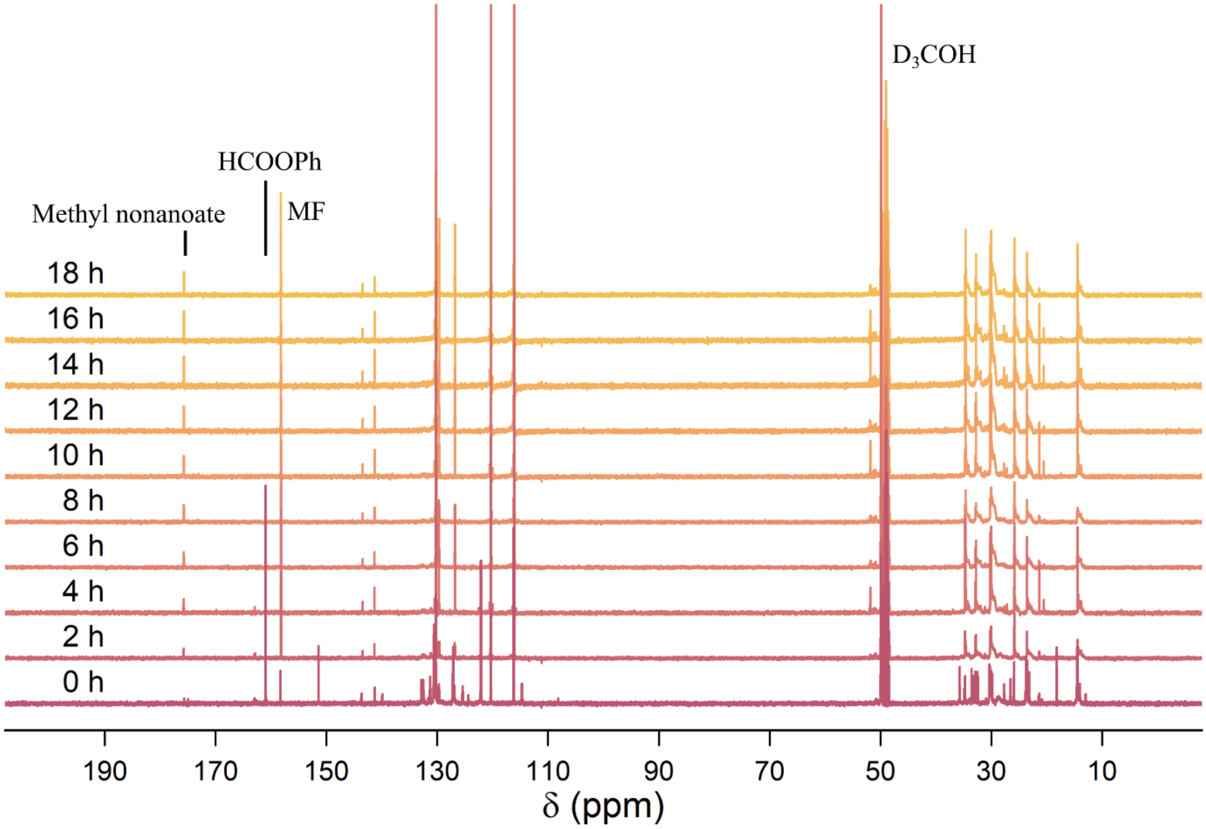

Figure S31. Ex-situ, background corrected ^{13}C-NMR spectra recorded at 25 °C of the hydroesterification of 1-octene with HCOOPh and [Pd(d^{t}bpx)] in d^4-MeOH. Field of view from 0 to 200 ppm. HCOOPh transforms rapidly into HCOOMe under acidic conditions and an excess of d^4-MeOH. Legend: **MF** = HCOOMe.

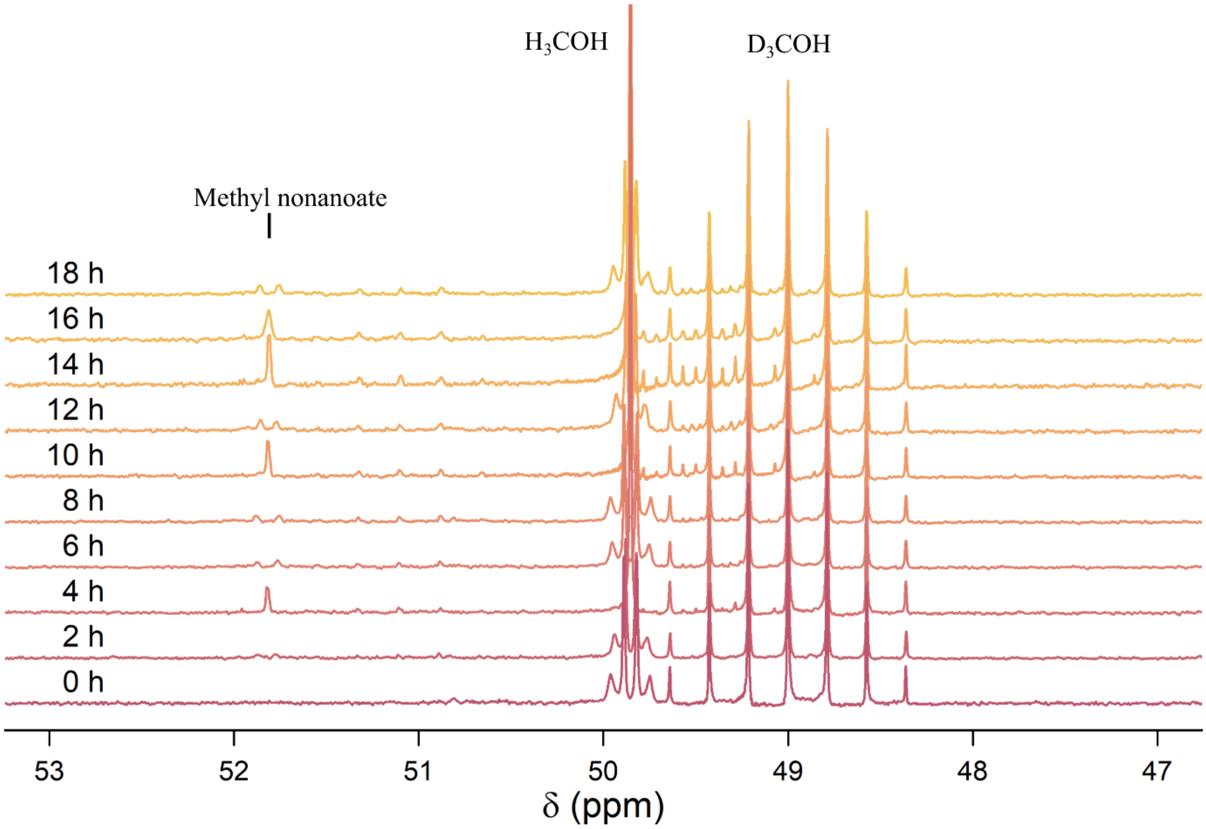

Figure S32. Ex-situ, background corrected ^{13}C-NMR spectra recorded at 25 °C of the hydroesterification of 1-octene with HCOOPh and [Pd(d^{t}bpx)] in d^4-MeOH. Field of view from 47 to 53 ppm. HCOOPh transforms rapidly into HCOOMe under acidic conditions and an excess of d^4-MeOH.

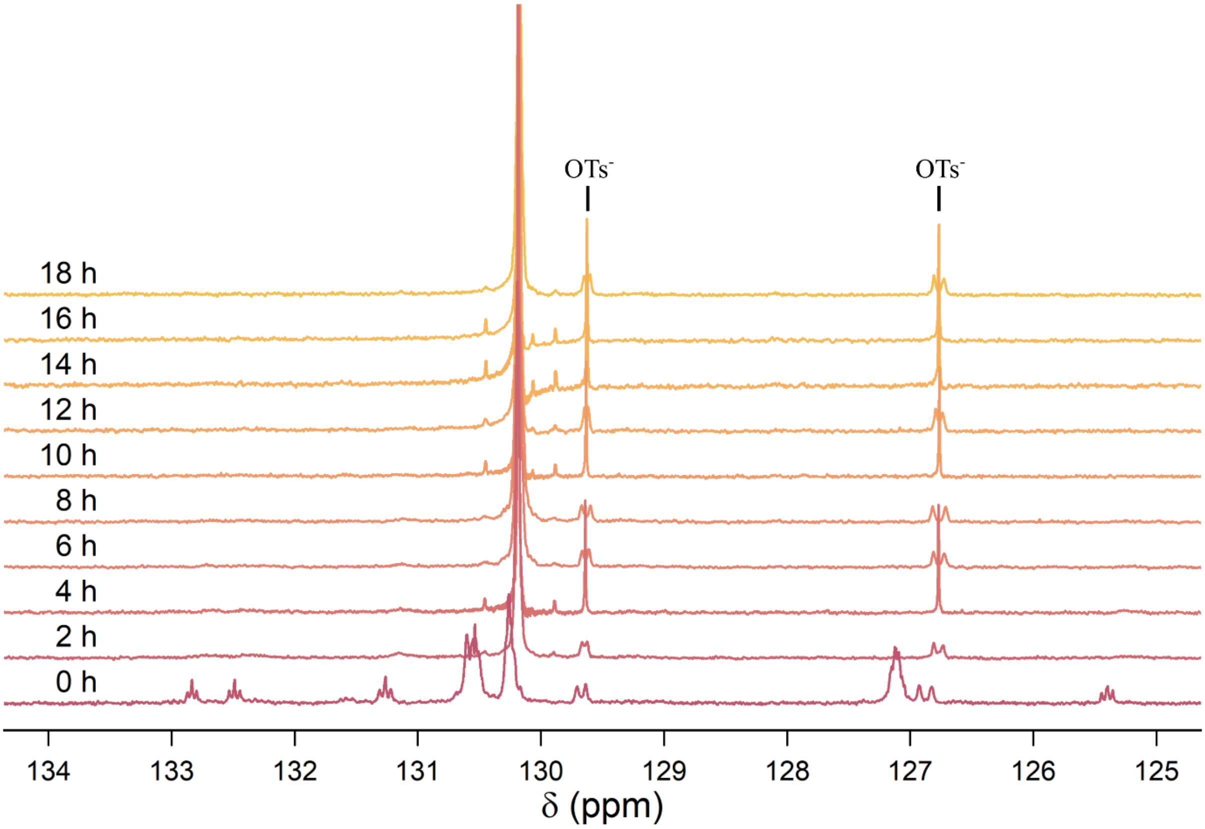

Figure S33. Ex-situ, background corrected ^{13}C-NMR spectra recorded at 25 °C of the hydroesterification of 1-octene with HCOOPh and [Pd(d^{t}bpx)] in d^4-MeOH. Field of view from 125 to 134 ppm. HCOOPh transforms rapidly into HCOOMe under acidic conditions and an excess of d^4-MeOH.

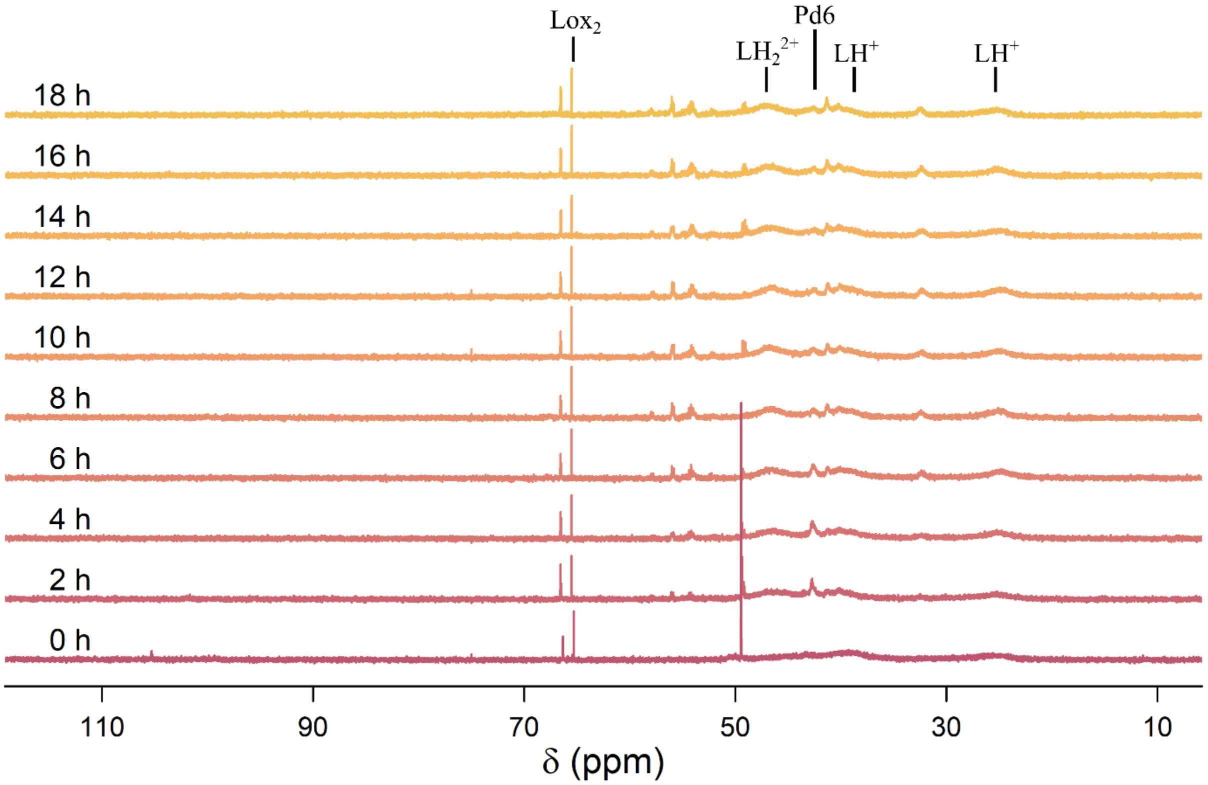

Figure S34. Ex-situ, background corrected ^{31}P-NMR spectra recorded at 25 °C of the hydroesterification of 1-octene with HCOOPh and [Pd(d^{t}bpx)] in d^4-MeOH. Field of view from 10 to 120 ppm. HCOOPh transforms rapidly into HCOOMe under acidic conditions and an excess of d^4-MeOH. Legend: **LH$^+$** = [H-d^{t}bpx]$^+$, **LH$_2^{2+}$** = [2H-d^{t}bpx]$^{2+}$, **Lox$_2$** = o-$C_6H_4(CH_2P(O)(^tBu)_3)_2$, **Pd6** = [Pd(d^{t}bpx)(μ-H)]$_2$.

4. In-situ NMR spectra

^{13}C-formaldehyde

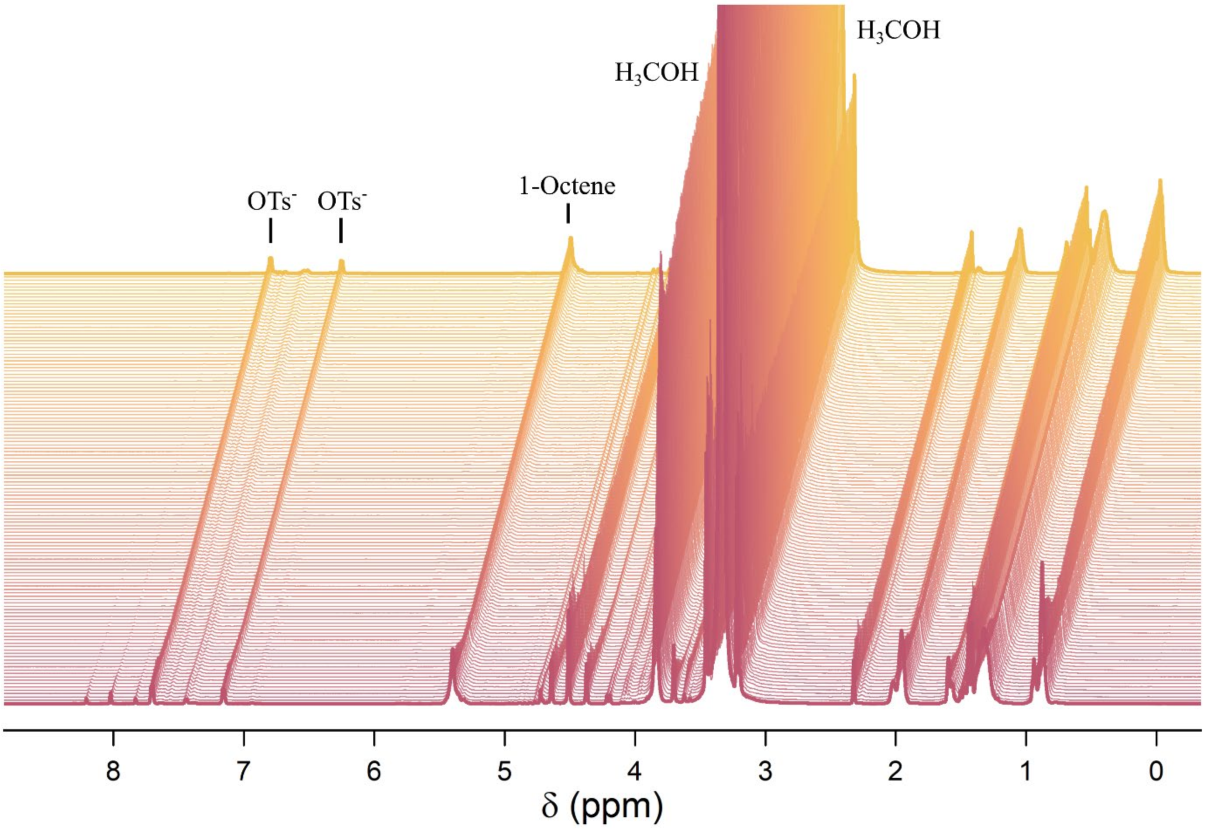

Figure S35. In-situ, background corrected ^{1}H-NMR spectra recorded at 100 °C of the hydroesterification of 1-octene with ^{13}C-PFA and [Pd(d^{t}bpx)] in d^{4}-MeOH. Field of view ranges from 0.5 to 8.5 ppm and is slightly tilted to increase readability.

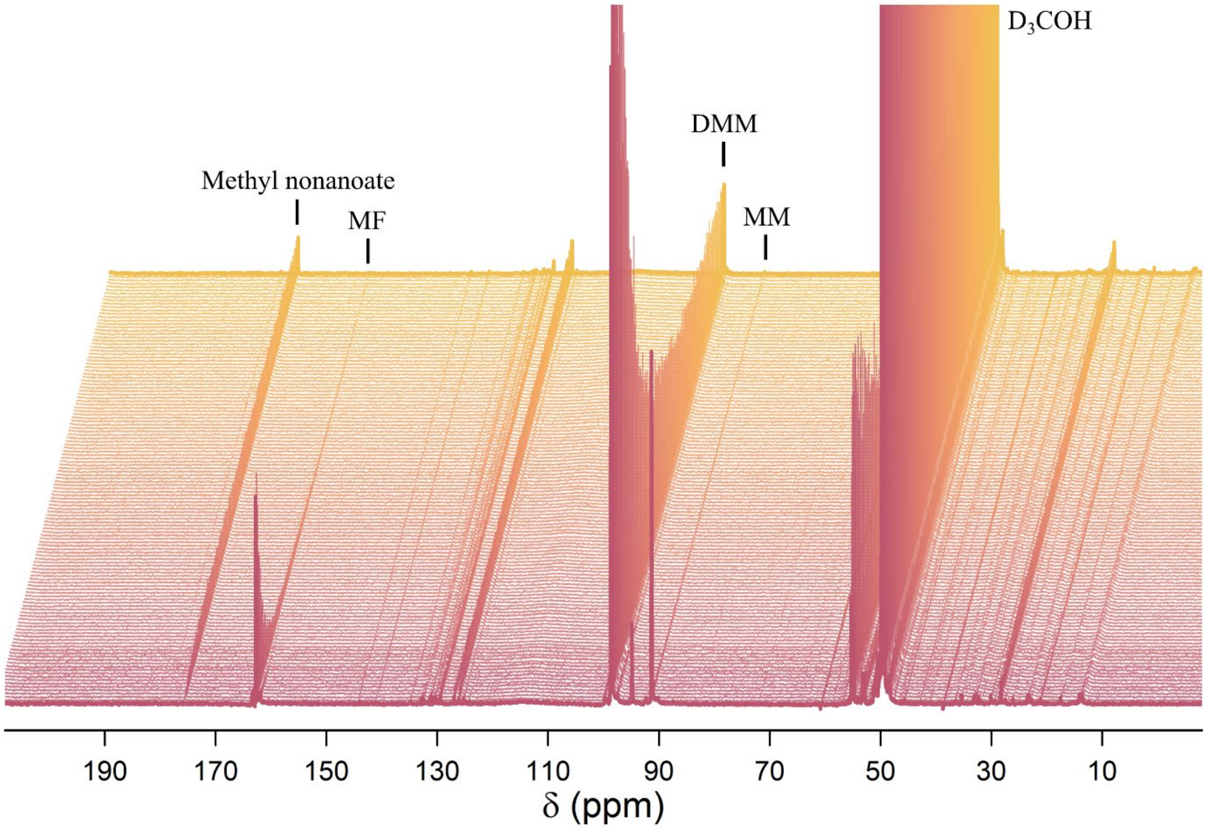

Figure S36. In-situ, background corrected ^{1}H-NMR spectra recorded at 100 °C of the hydroesterification of 1-octene with ^{13}C-PFA and [Pd(d^{t}bpx)] in d^{4}-MeOH. Field of view ranges from 0 to 200 ppm and is slightly tilted to increase readability. Legend: **MM** = $H_2C(OMe)(OH)$, **DMM** = $H_2C(OMe)_2$, **MF** = HCOOMe.

HCOOMe

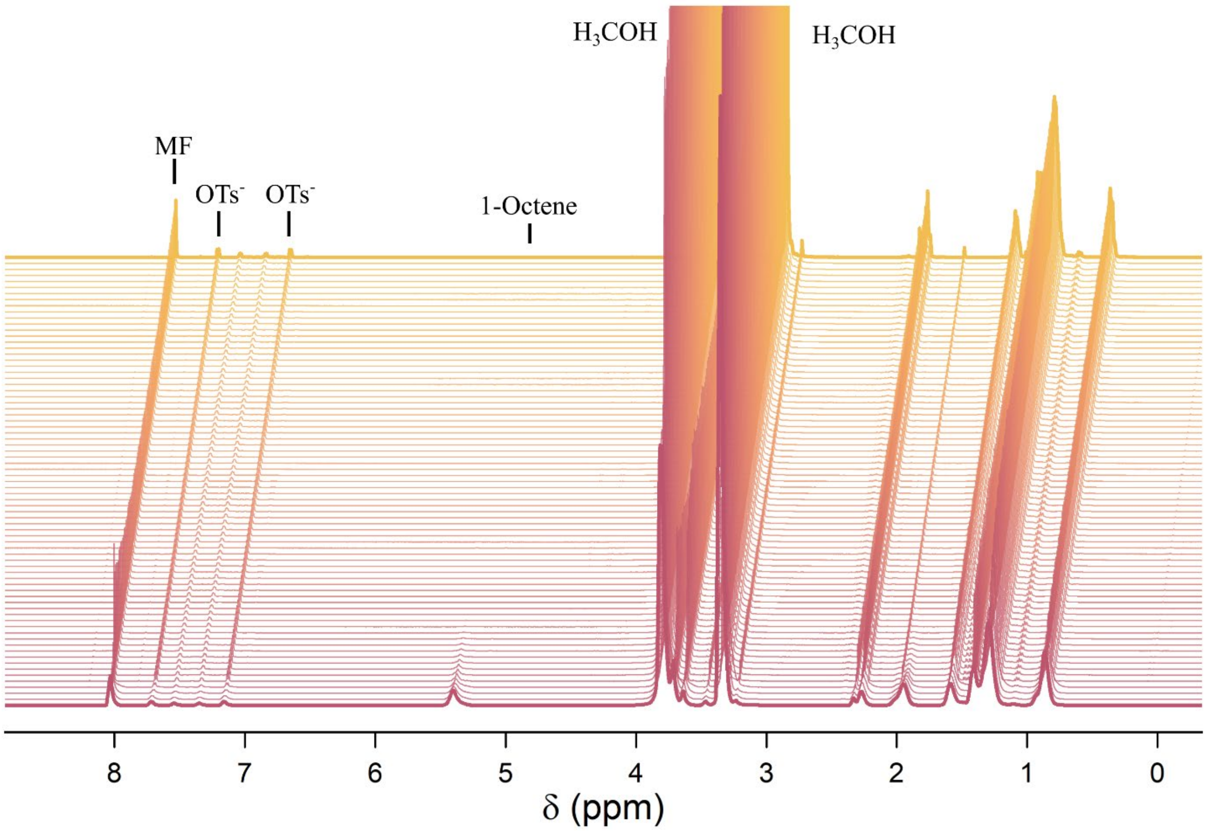

Figure S37. In-situ, background corrected ^{1}H-NMR spectra recorded at 100 °C of the hydroesterification of 1-octene with HCOOMe and [Pd(d^{t}bpx)] in d^{4}-MeOH. Field of view ranges from 0.5 to 8.5 ppm and is slightly tilted to increase readability. Legend: **MF** = HCOOMe.

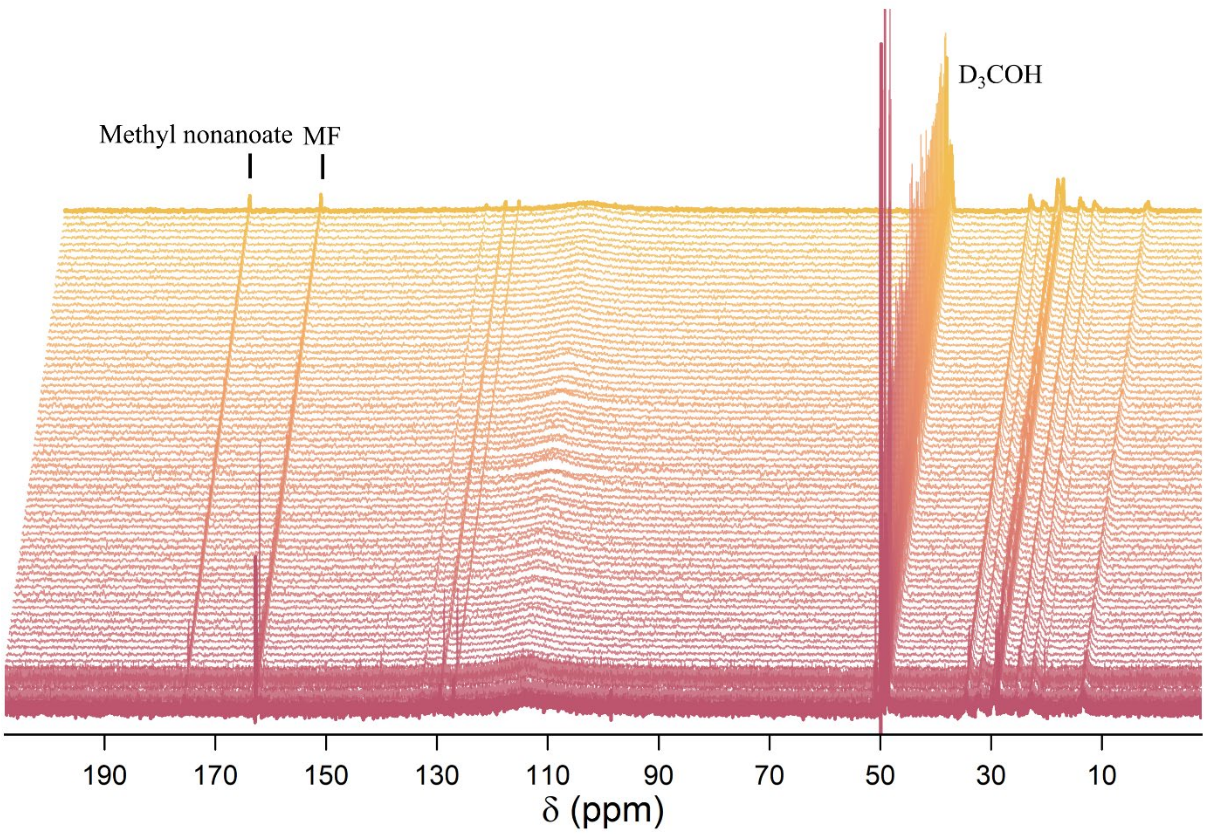

Figure S38. In-situ, background corrected ^{1}H-NMR spectra recorded at 100 °C of the hydroesterification of 1-octene with HCOOMe and [Pd(d^{t}bpx)] in d^4-MeOH. Field of view ranges from 0 to 200 ppm and is slightly tilted to increase readability. The first five spectra show an increased level of noise due to suboptimal shimming. Legend: **MF** = HCOOMe.

HCOOH

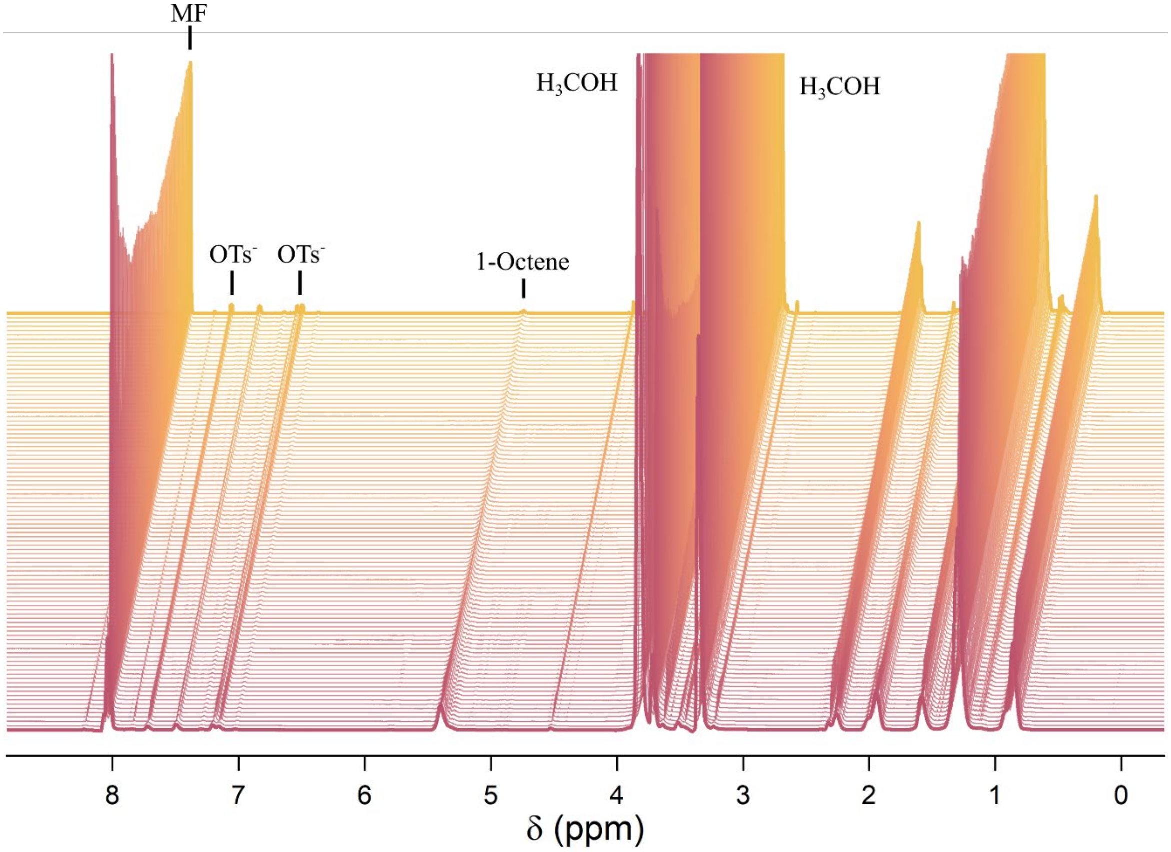

Figure S39. In-situ, background corrected ^{1}H-NMR spectra recorded at 100 °C of the hydroesterification of 1-octene with HCOOH and [Pd(d^{t}bpx)] in d[4]-MeOH. Field of view ranges from 0.5 to 8.5 ppm and is slightly tilted to increase readability. Legend: **MF** = HCOOMe.

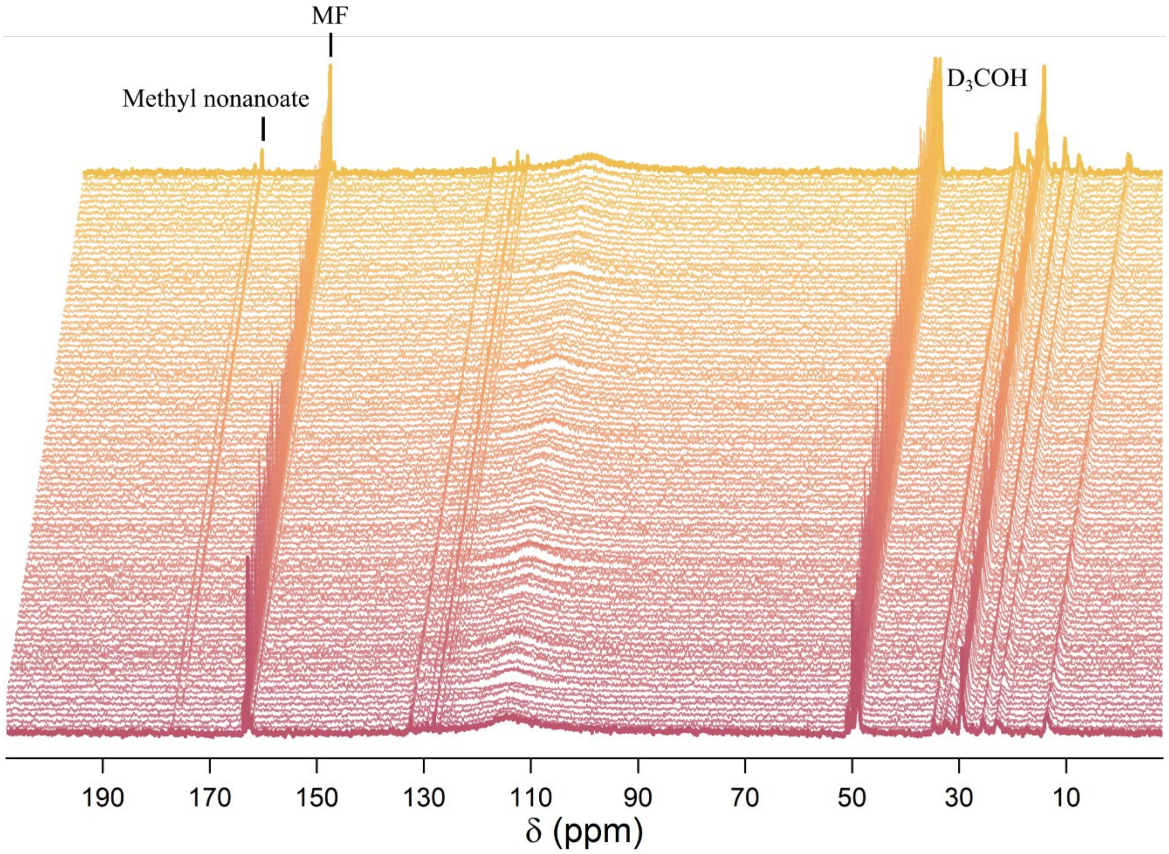

Figure S40. In-situ, background corrected ^{1}H-NMR spectra recorded at 100 °C of the hydroesterification of 1-octene with HCOOH and [Pd(d^{t}bpx)] in d^4-MeOH. Field of view ranges from 0 to 200 ppm and is slightly tilted to increase readability. Legend: **MF** = HCOOMe.

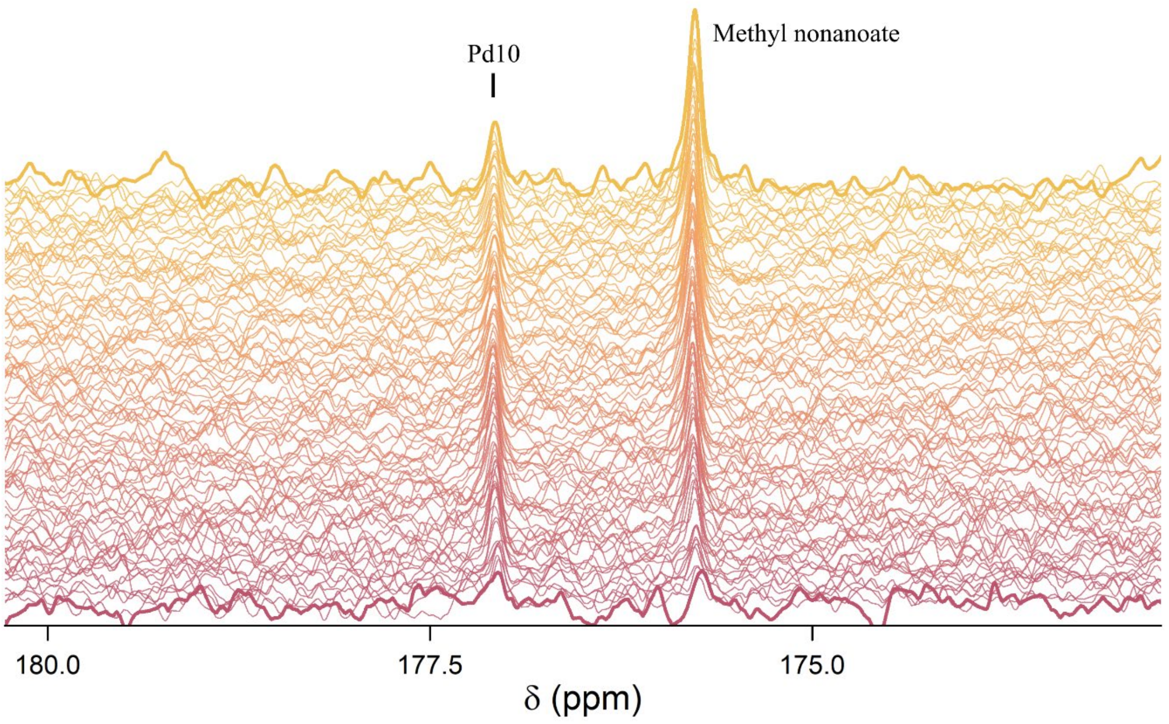

Figure S41. In-situ, background corrected ^{1}H-NMR spectra recorded at 100 °C of the hydroesterification of 1-octene with HCOOH and [Pd(d^{t}bpx)] in d^{4}-MeOH. Field of view ranges from 172 to 180 ppm. The signal at 177 ppm is assigned to an intermediary Pd-C(O)OMe group. Legend: **Pd8** = [Pd(d^{t}bpx)(C(O)OMe)(H)]$^{+}$.

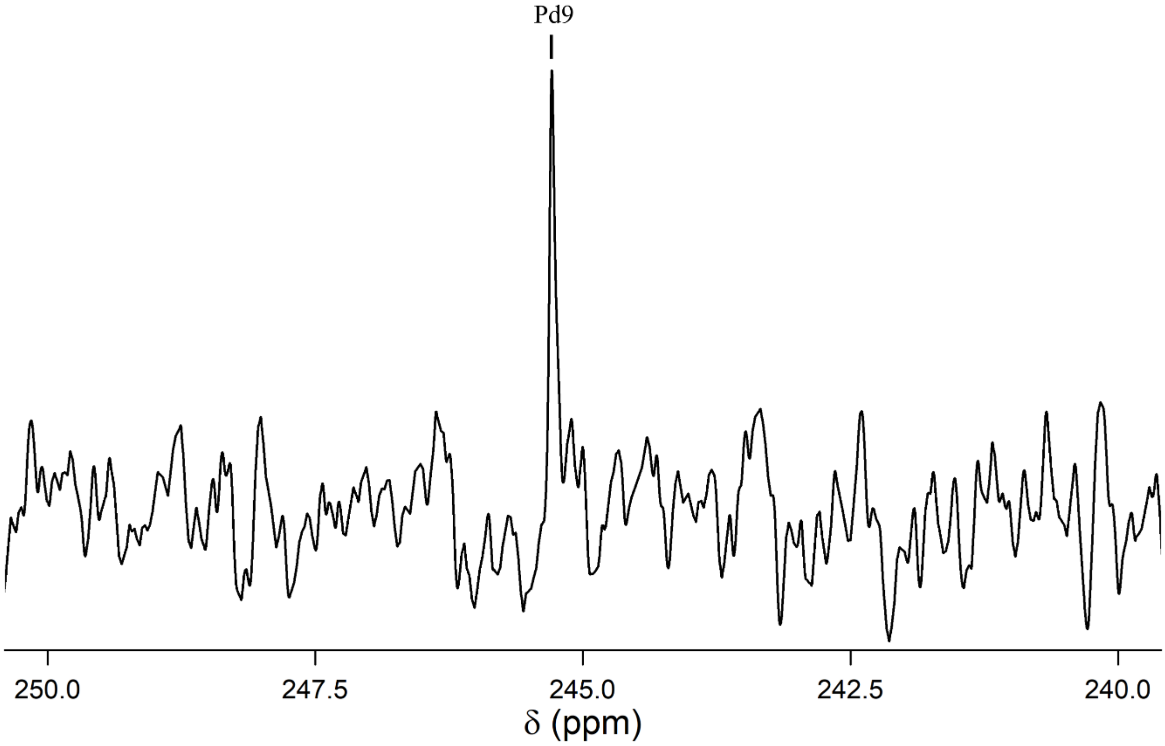

Figure S42. In-situ, background corrected ^{1}H-NMR spectrum recorded at 100 °C after 1471 min of the hydroesterification of 1-octene with HCOOH and [Pd(d^{t}bpx)] in d^{4}-MeOH. Field of view ranges from 240 to 250 ppm. The signal is assigned to intermediary Pd-CHO group. Legend: **Pd7** = [Pd(d^{t}bpx)(CHO)(H)]$^{+}$.

Pd(OAc)$_2$ + d^{t}bpx + TsOH + H^{13}COOMe

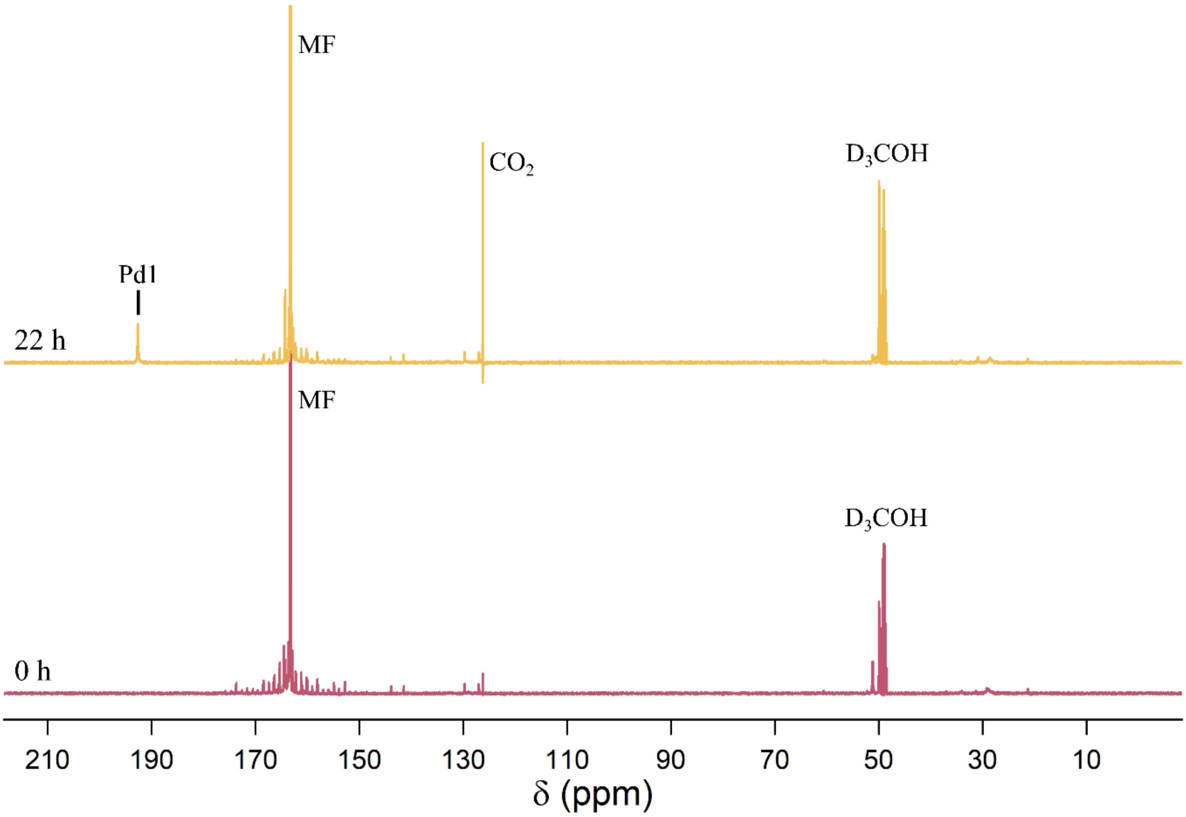

Figure S43. Background corrected ^{13}C-NMR spectra recorded at 25 °C of the decomposition of H^{13}COOMe by [Pd(d^{t}bpx)] in d^4-MeOH. Field of view from 0 to 210 ppm. The signal at 192.6 ppm can be assigned to [Pd(d^{t}bpx)(CO)]. Legend: **MF** = HCOOMe, **Pd1** = [Pd(d^{t}bpx)(CO)].

5. Kinetic modeling

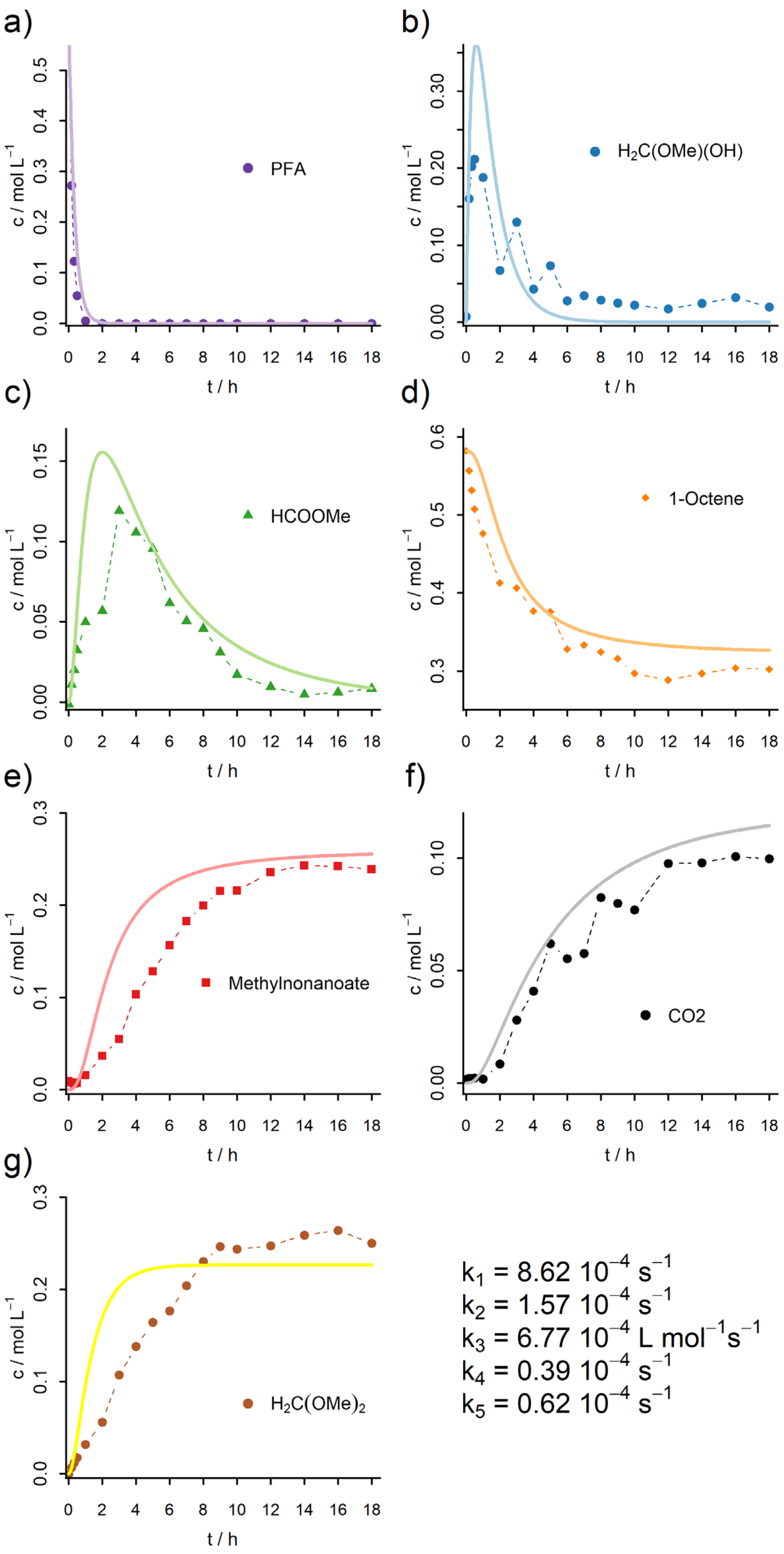

Figure S44. Experimental concentrations and their kinetic fits from the hydroesterification of 1-octene with ^{13}C-PFA and [Pd(d^{t}bpx)] in d^4-MeOH at 100 °C.

6. NMR spectra of simple mixtures

d^{t}bpx + TsOH

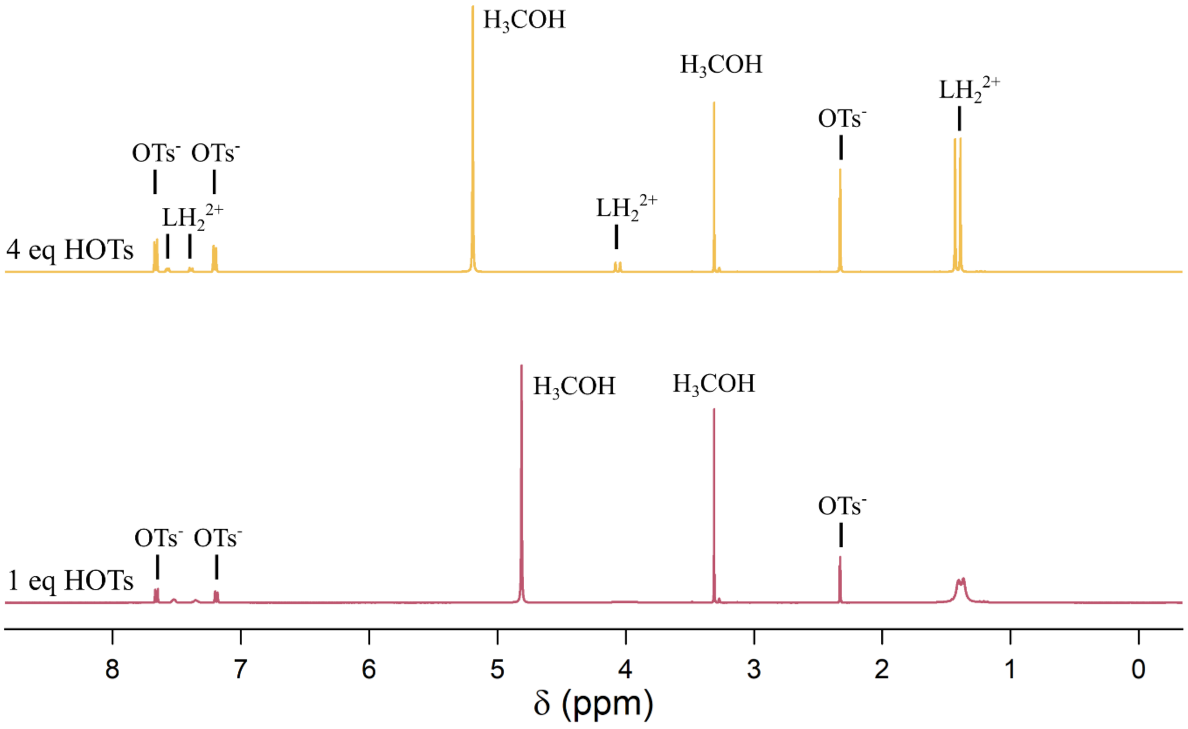

Figure S45. Background corrected ^{1}H-NMR spectra recorded at 25 °C of the protonation of d^{t}bpx with TsOH in d^4-MeOH. Field of view from -0.5 to 9 ppm. Legend: **LH$_2$$^{2+}$** = [2H-d^{t}bpx]$^{2+}$.

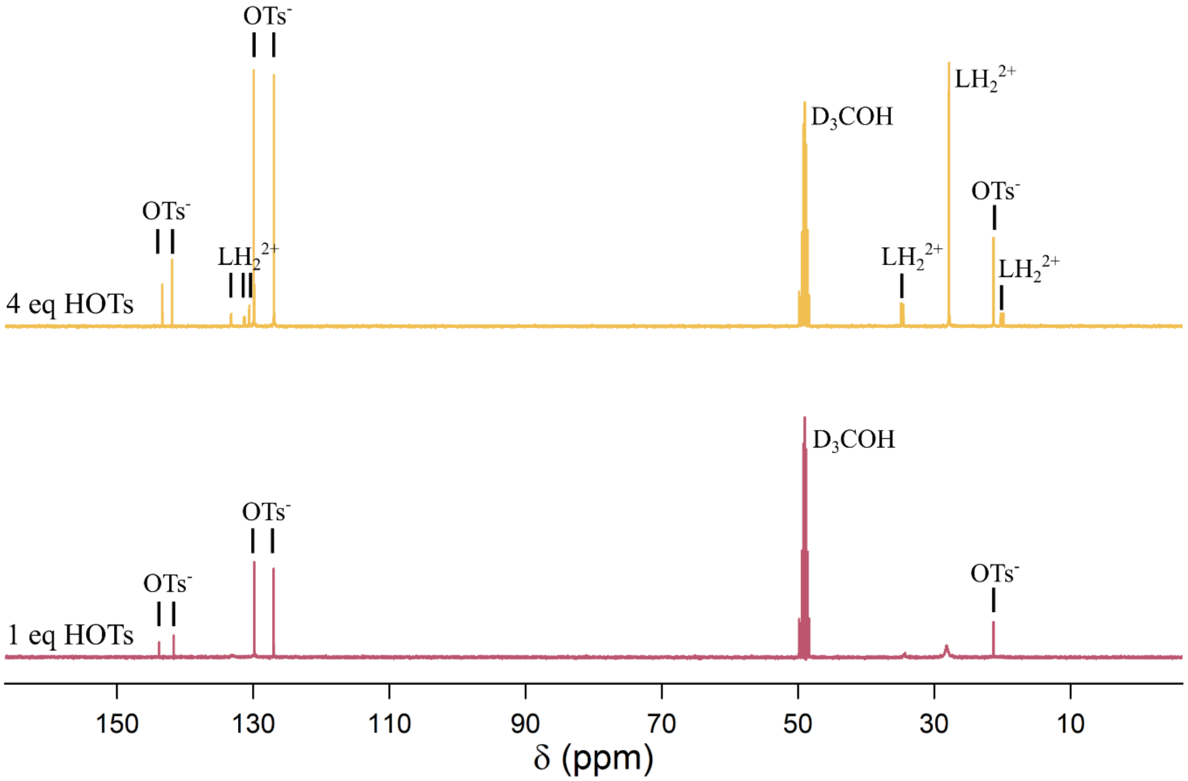

Figure S46. Background corrected ^{13}C-NMR spectra recorded at 25 °C of the protonation of d^{t}bpx with TsOH in d^4-MeOH. Field of view from 0 to 160 ppm. Legend: $\mathbf{LH_2^{2+}}$ = [2H-d^{t}bpx]$^{2+}$.

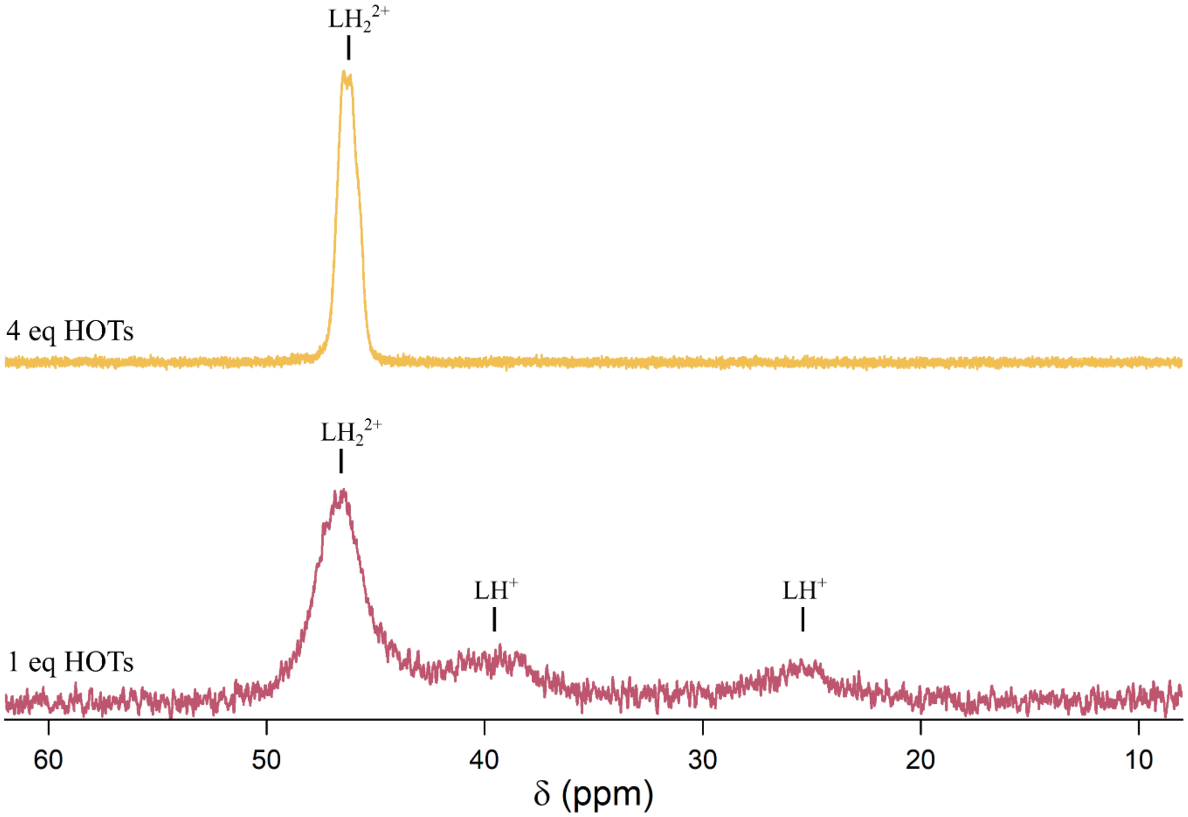

Figure S47. Background corrected ^{31}P-NMR spectra recorded at 25 °C of the protonation of d^{t}bpx with TsOH in d^4-MeOH. Field of view from 10 to 60 ppm. Legend: **LH$^+$** = [H-d^{t}bpx]$^+$, **LH$_2^{2+}$** = [2H-d^{t}bpx]$^{2+}$.

Pd(OAc)₂ + d*ᵗ*bpx + TsOH + H₂O

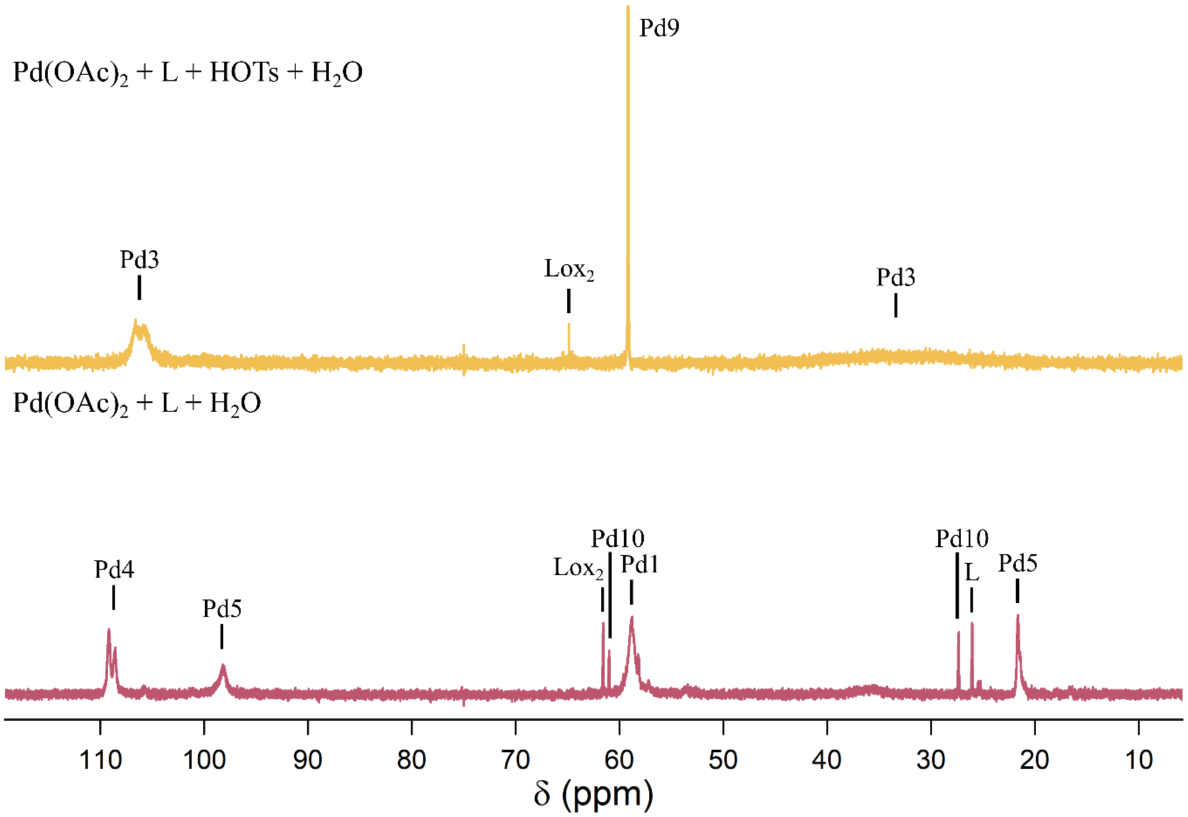

Figure S48. Background corrected ^{31}P-NMR spectra recorded at 25 °C of the mixture of $Pd(OAc)_2$ + d^{t}bpx (bottom) and $Pd(OAc)_2$ + d^{t}bpx + TsOH in D_2CCl_2. Field of view from 10 to 115 ppm. All signals can be assigned to different Pd-d^{t}bpx complexes or the oxidized ligand. Legend: **L** = d^{t}bpx, **Lox₂** = o-$C_6H_4(CH_2P(O)(^tBu)_3)_2$, **Pd3** = [Pd($\eta^2$-CP-d^{t}bpx-H)($\eta^2$-OTs)][OTs], **Pd4** = [$Pd_2$($\eta^4$-CP-d^{t}bpx)($\mu$-OAc)$_2$]$_2$, **Pd5** = [Pd($\eta^2$-CP-d^{t}bpx)($\eta^2$-OAc)], **Pd9** = [Pd(d^{t}bpx)$(H_2O)_2$]$^{2+}$, **Pd10** = [Pd(d^{t}bpx)(H_2O)(OAc)]$^+$.

7. DFT calculated structures and energies

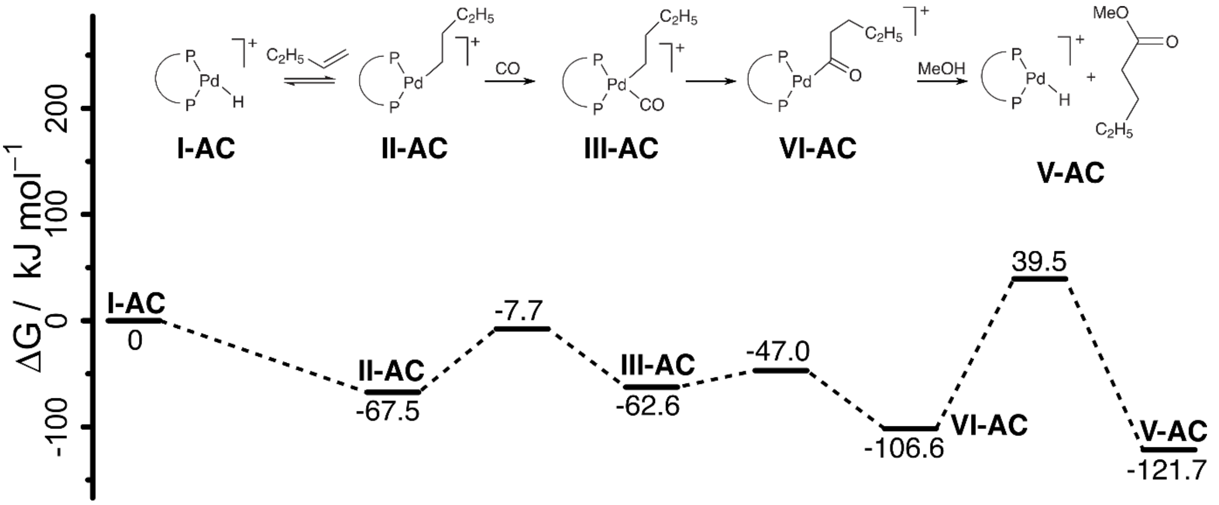

Figure S49. Reaction sequence as well as the respective Gibbs free energies and activation energies for the alternative alkoxy carbonylation reaction pathway (compare Scheme 3). The activation energy for the final elimination step is very high with 141.1 kJ mol^{-1}.

Table S2. Aggregation of DFT calculated (absolute) enthalpies (*H*) and Gibbs free energies (*G*) at 298.15 K.

Compound	*H* / kJ mol^{-1}	*G* / kJ mol^{-1}
HCOOMe (**MF**)	-600800.0	-600884.7
CO	-297252.0	-297310.9
CO_2	-494723.6	-494780.8
MeOH	-303454.5	-303525.3
CH_4	-106179.1	-106234.7
C_4H_8	-412220.9	-412309.5
C_4H_9COOMe	-1013143.4	-1013267.4
[Pd(d^{t}bpx)] (**I**)	-4595705.6	-4595963.8
[Pd(d^{t}bpx)(H)(COOMe)] (**II**)	-5196554.4	-5196837.1
[Pd(d^{t}bpx)(C_4H_9)(COOMe)] (**IVa**)	-5608836.9	-5609153.8
[Pd(d^{t}bpx)(H)(C_4H_8COOMe)] (**IVb**)	-5608841.1	-5609152.2
[Pd(d^{t}bpx)(CO)] (**IV-CO**)	-4893093.0	-4893368.8
[Pd(d^{t}bpx)(CO_2)] (**IV-CO$_2$**)	-5090456.0	-5090738.4
[Pd(d^{t}bpx)(H)]$^+$ (**I-AC**)	-4596744.5	-4597000.0
[Pd(d^{t}bpx)(C_4H_9)]$^+$ (**II-AC**)	-5009086.0	-5009376.9
[Pd(d^{t}bpx)(C_4H_9)(CO)]$^+$ (**III-AC**)	-5306382.3	-5306683.0
[Pd(d^{t}bpx)(C(O)C_4H_9)]$^+$ (**IV-AC**)	-5306409.7	-5306722.0

8. Pure component NMR spectra

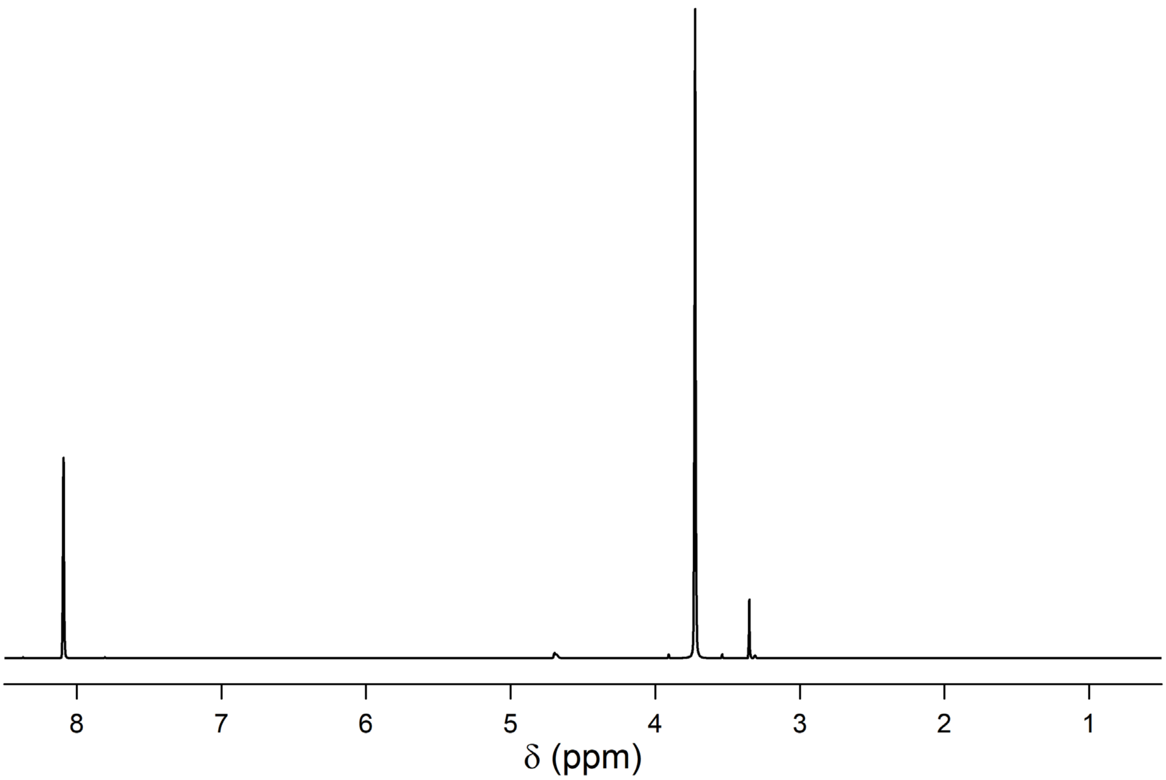

Figure S50. Background corrected ^{1}H-NMR spectrum of HCOOMe in d^{4}-MeOH.

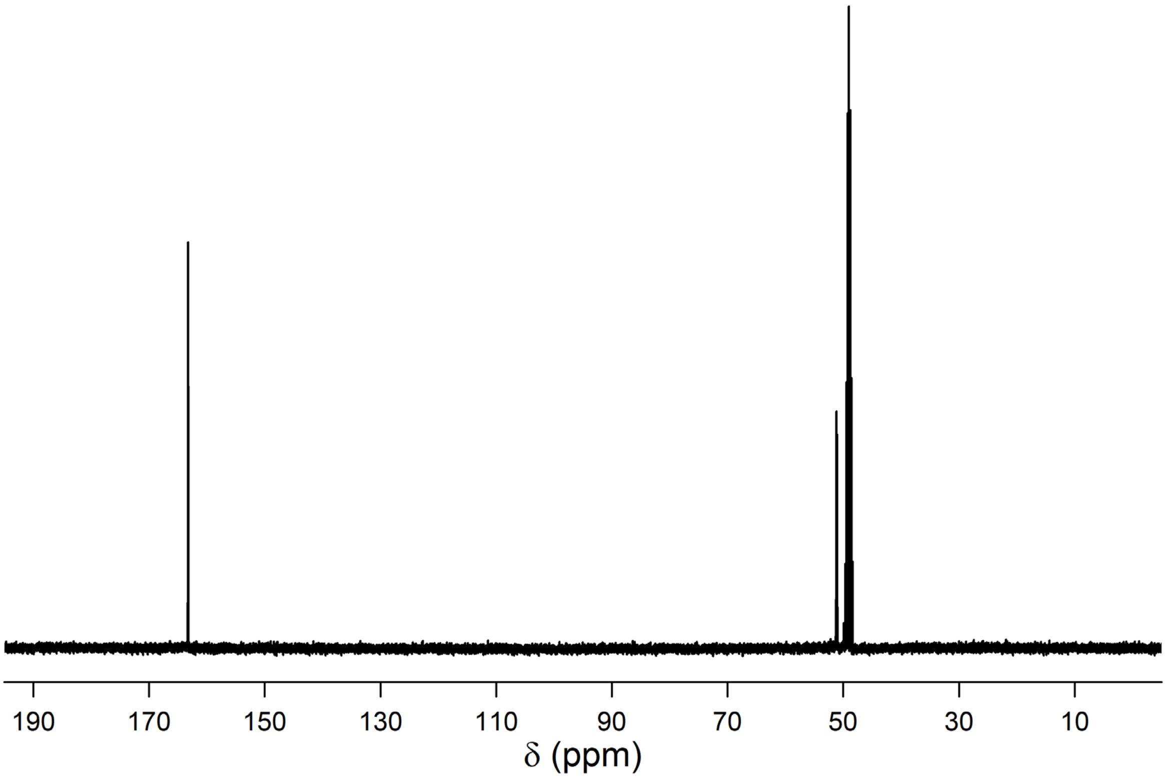

Figure S51. Background corrected ^{13}C-NMR spectrum of HCOOMe in d^{4}-MeOH.

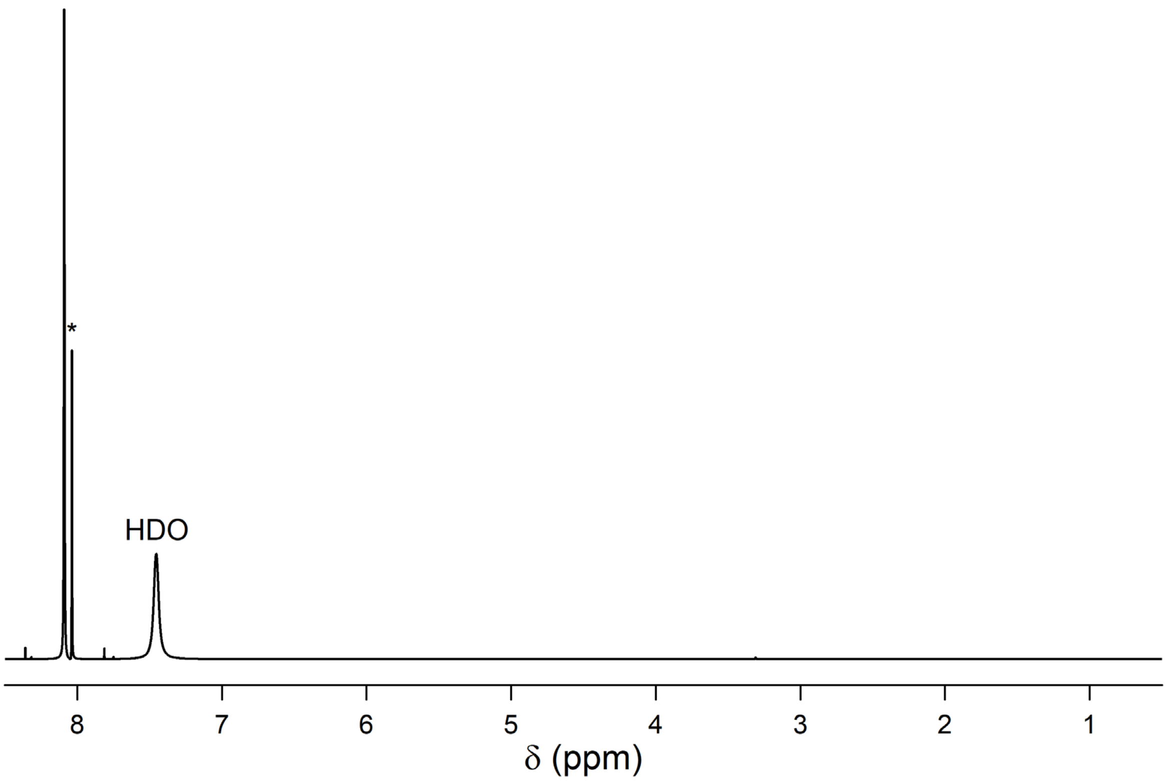

Figure S52. Background corrected ^{1}H-NMR spectrum of HCOOH. The star (*) marks residual peaks from HCOOMe in d^4-MeOH.

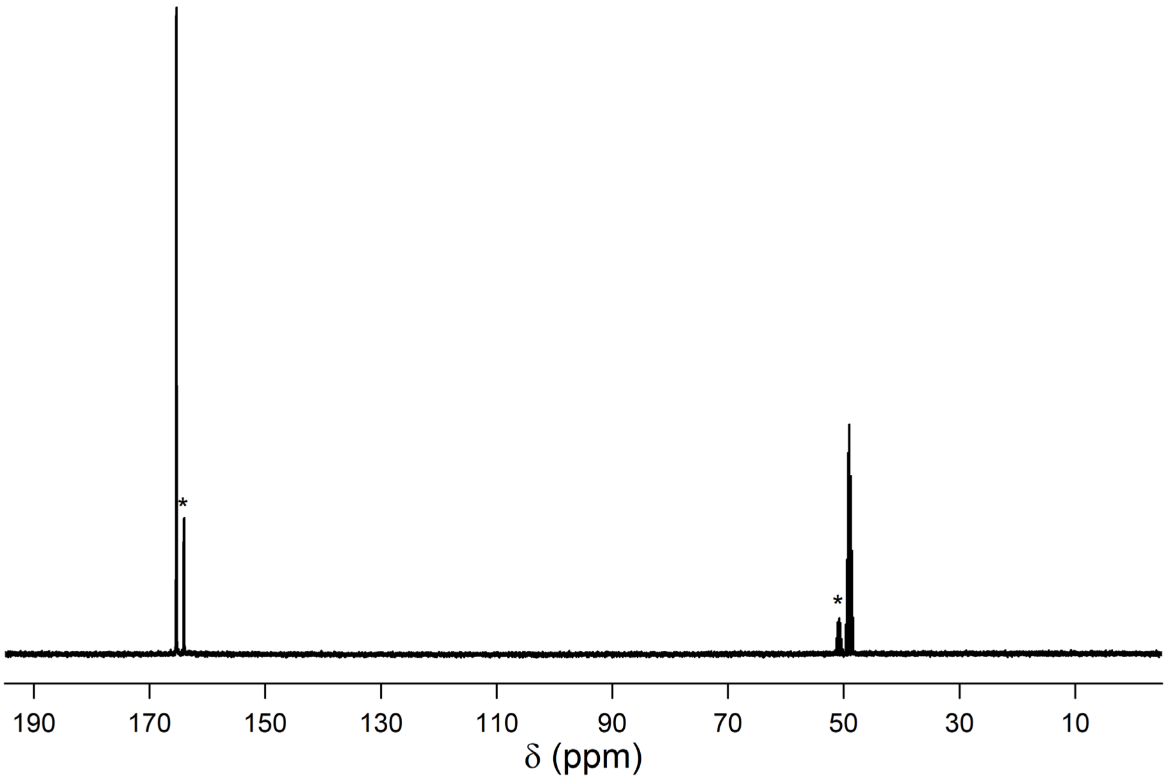

Figure S53. Background corrected ^{13}C-NMR spectrum of HCOOH. The star (*) marks residual peaks from HCOOMe in d^4-MeOH.

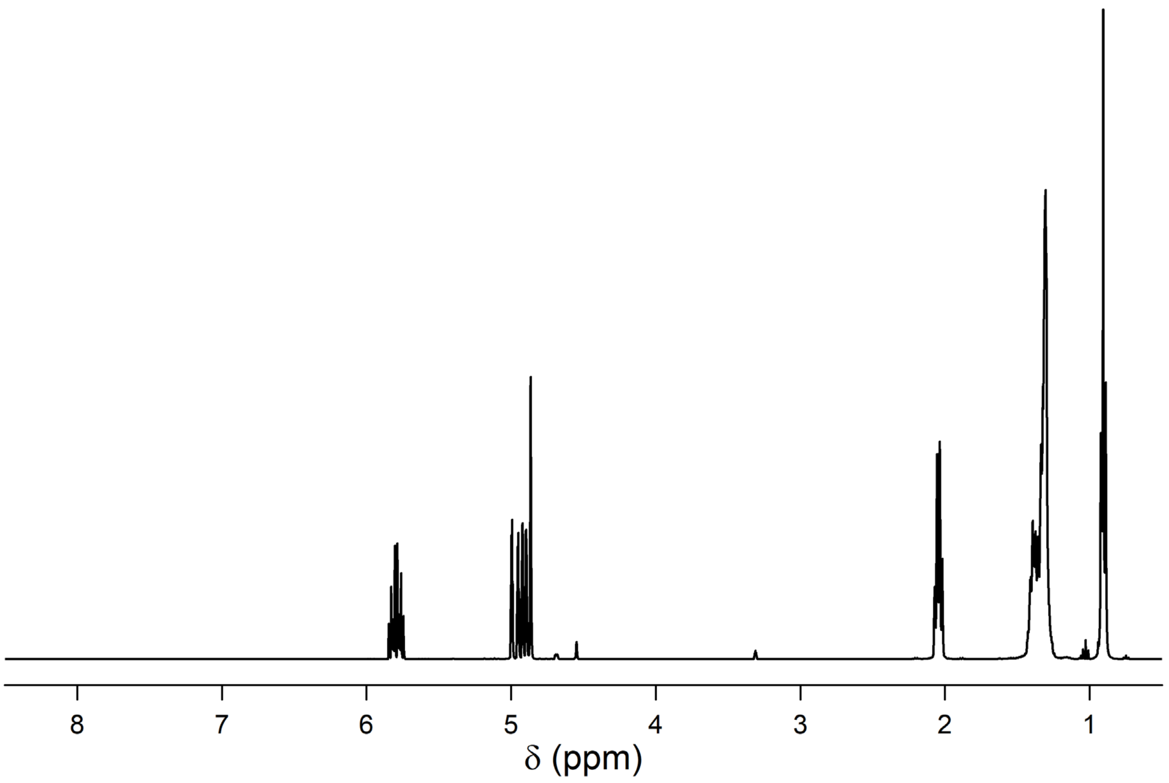

Figure S54. Background corrected ^{1}H-NMR spectrum of 1-octene in d^4-MeOH.

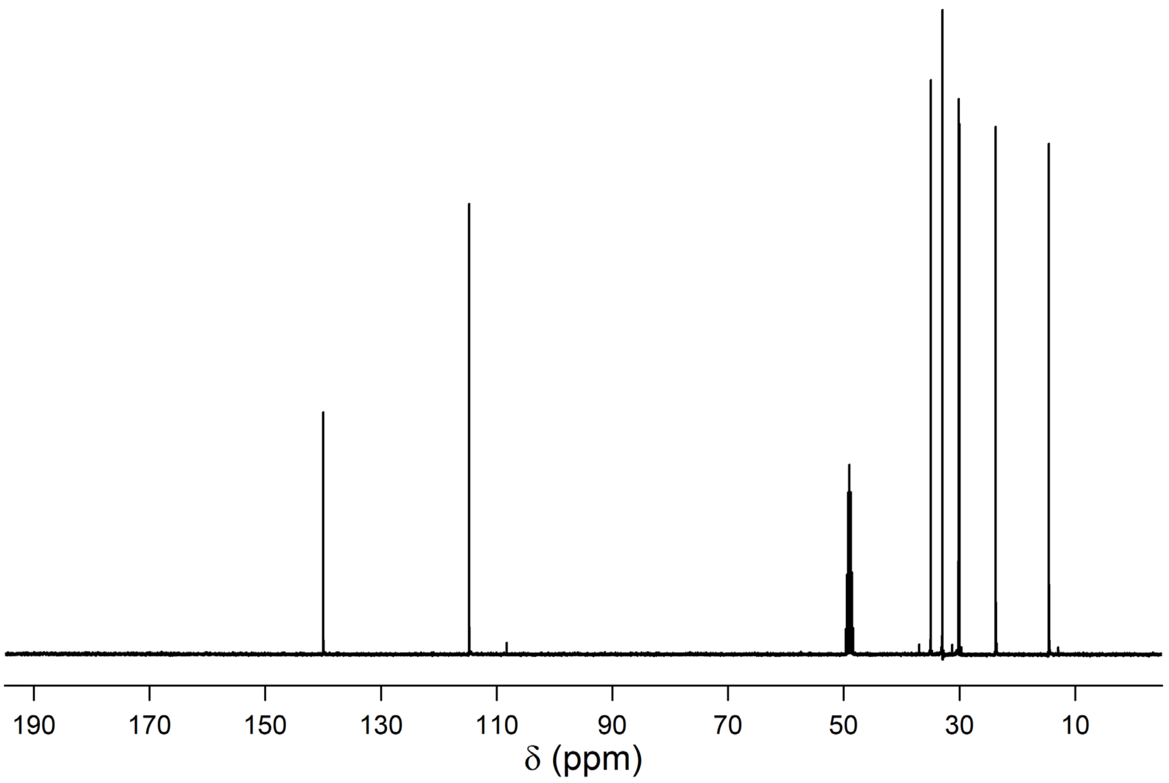

Figure S55. Background corrected ^{13}C-NMR spectrum of 1-octene in d^4-MeOH.

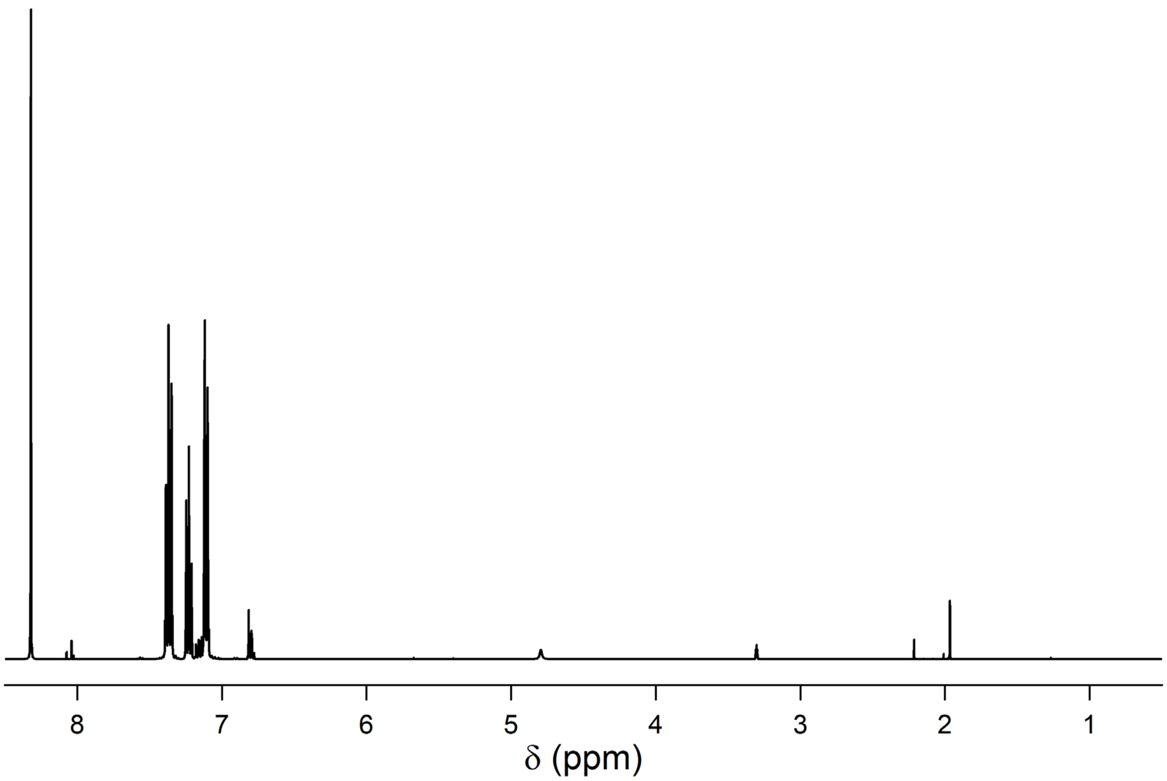

Figure S56. Background corrected ^{1}H-NMR spectrum of HCOOPh in d[4]-MeOH.

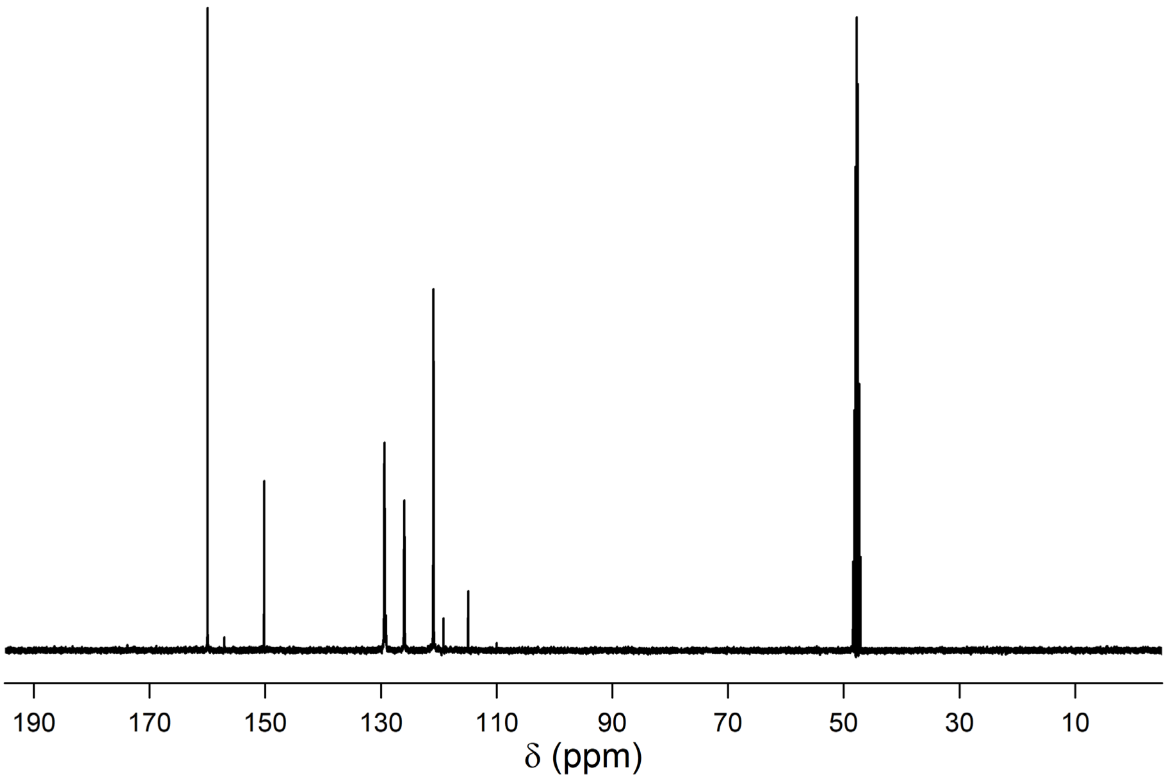

Figure S57. Background corrected ^{13}C-NMR spectrum of HCOOPh in d[4]-MeOH.

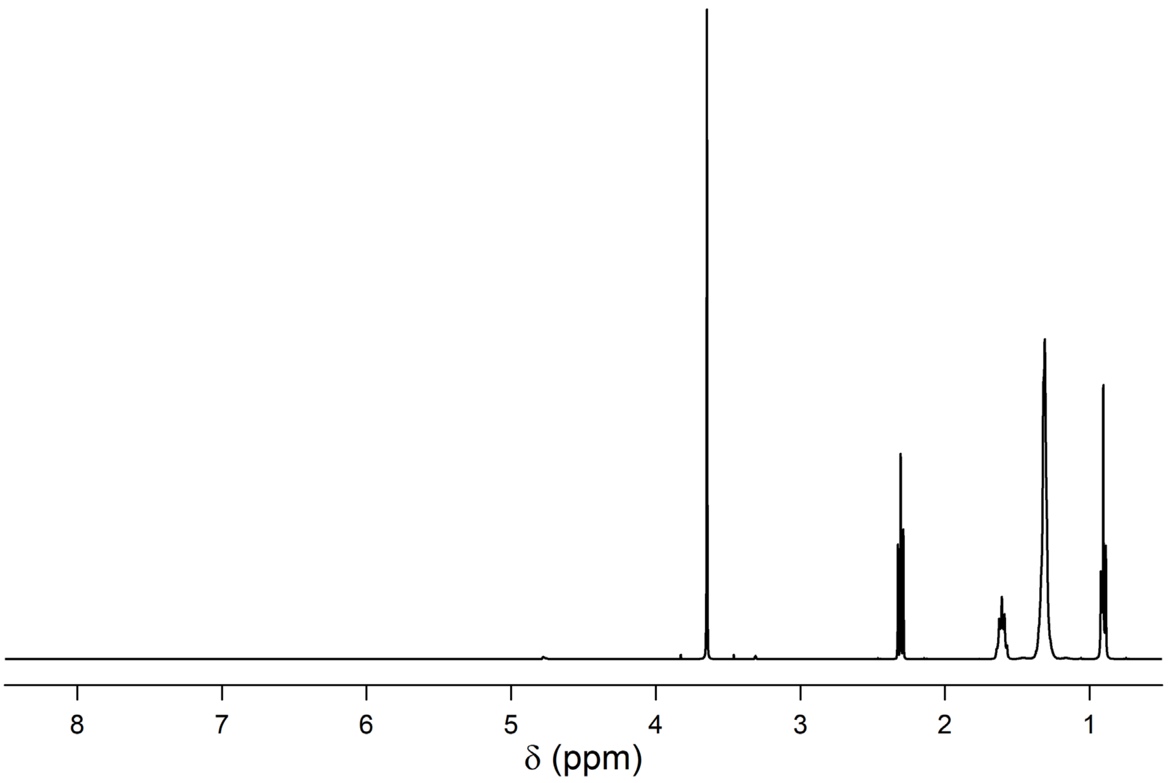

Figure S58. Background corrected ^{1}H-NMR spectrum of methyl nonanoate in d^4-MeOH.

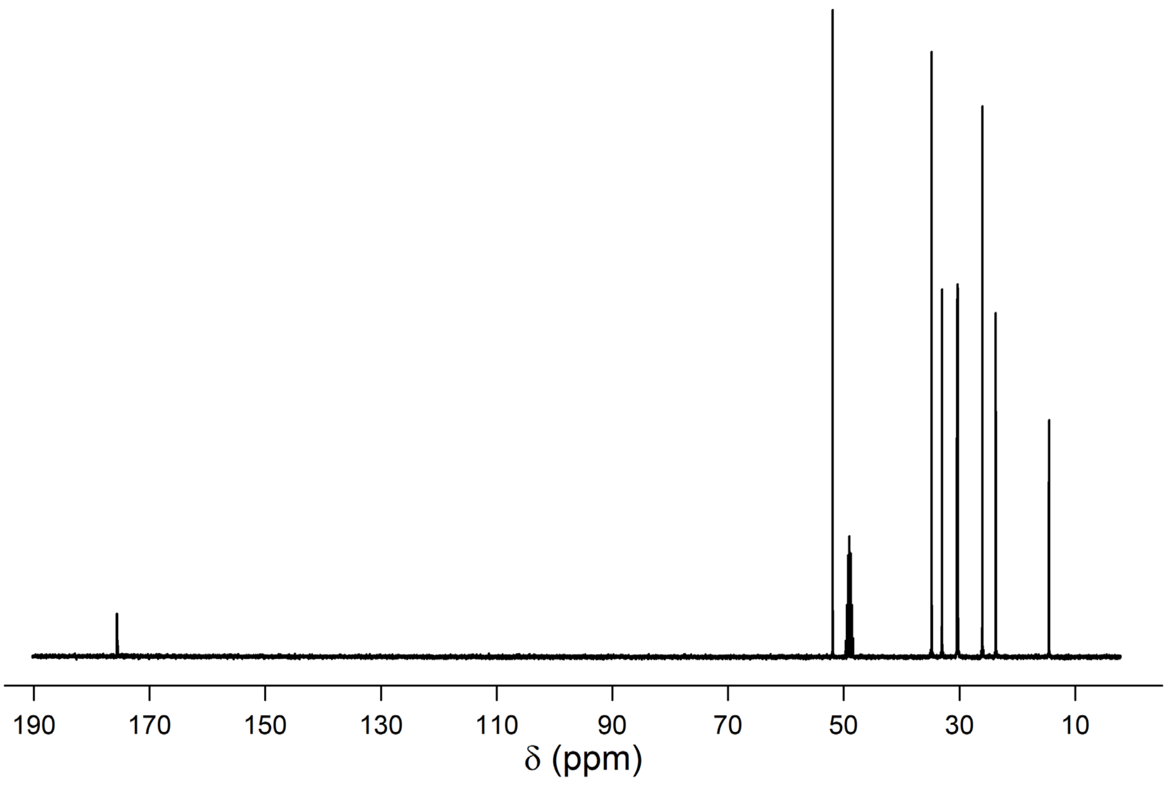

Figure S59. Background corrected ^{13}C-NMR spectrum of methyl nonanoate in d^4-MeOH.

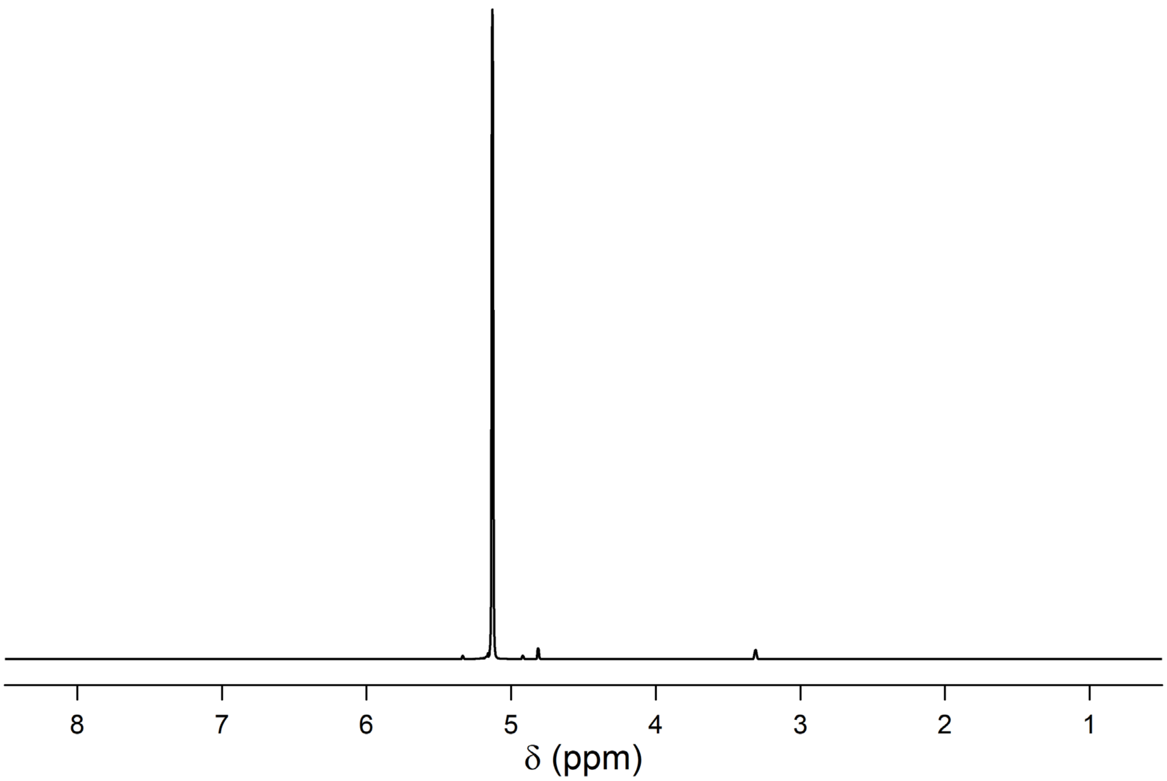

Figure S60. Background corrected ^{1}H-NMR spectrum of 1,3,5-trioxane in d^4-MeOH.

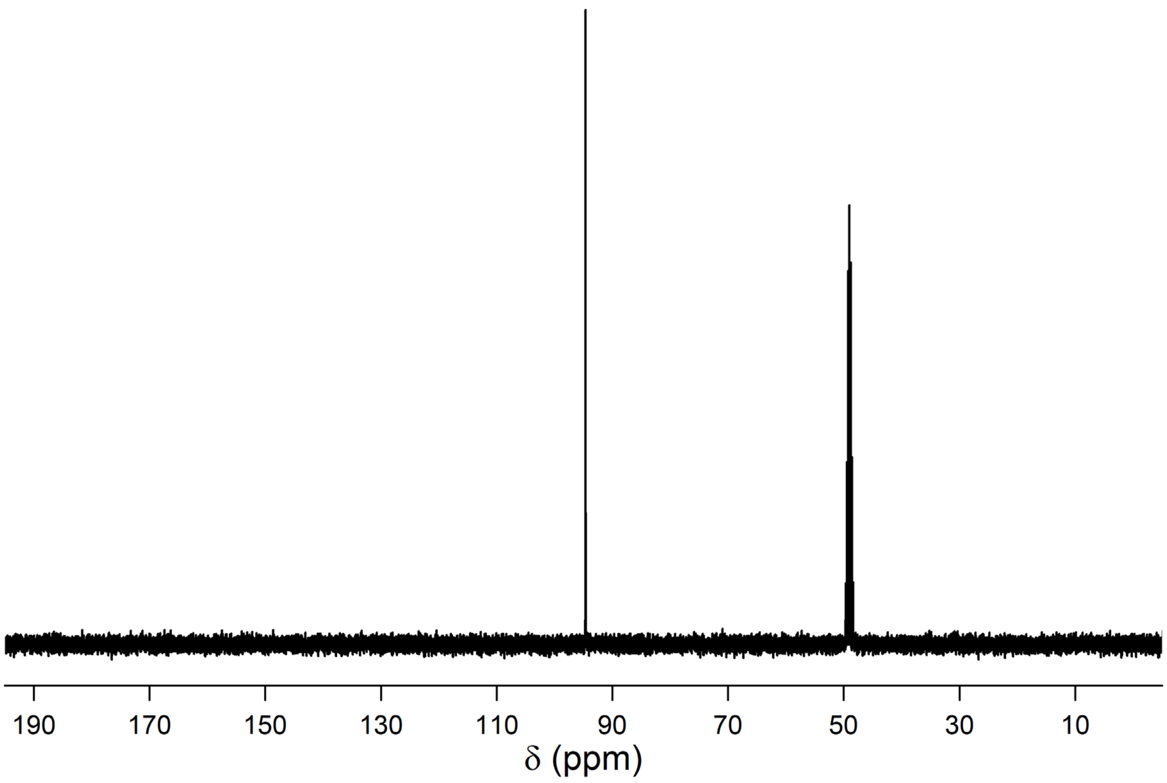

Figure S61. Background corrected ^{13}C-NMR spectrum of 1,3,5-trioxane in d^4-MeOH.

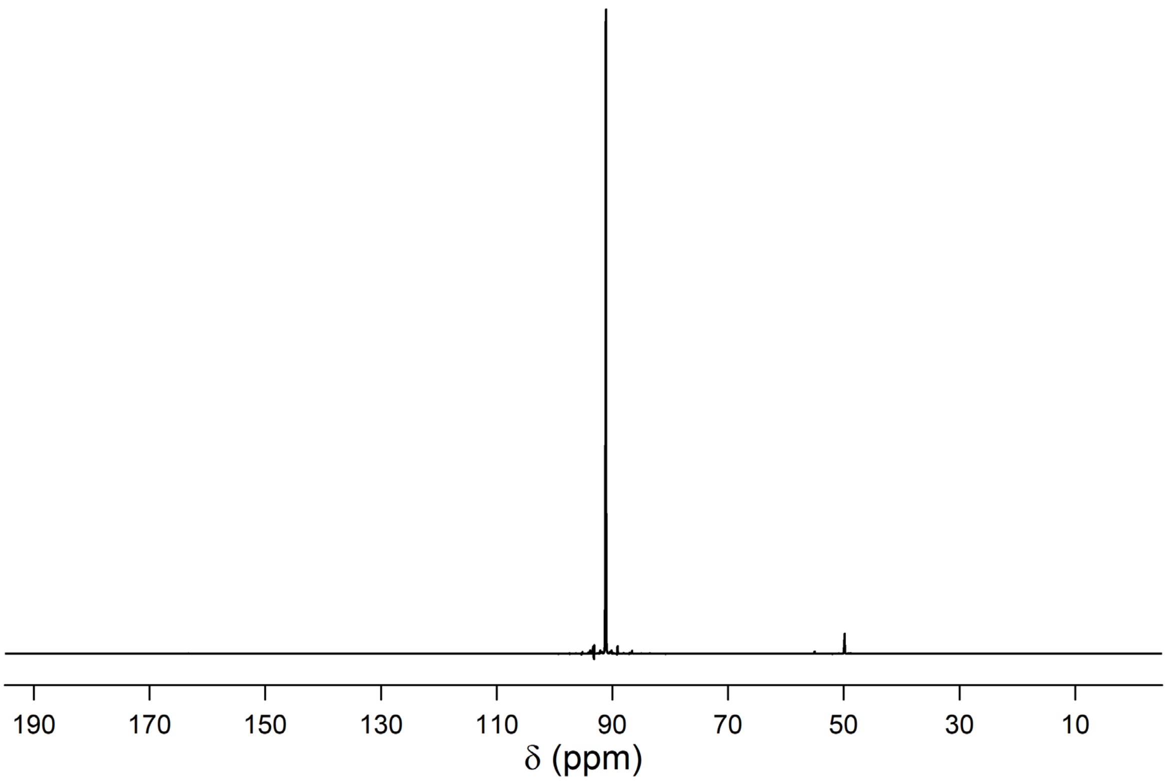

Figure S62. Background corrected ^{13}C-NMR spectrum of $H_2{}^{13}C(OMe)(OH)$ (**MM**) in d^4-MeOH.

9. Variable temperature NMR spectra

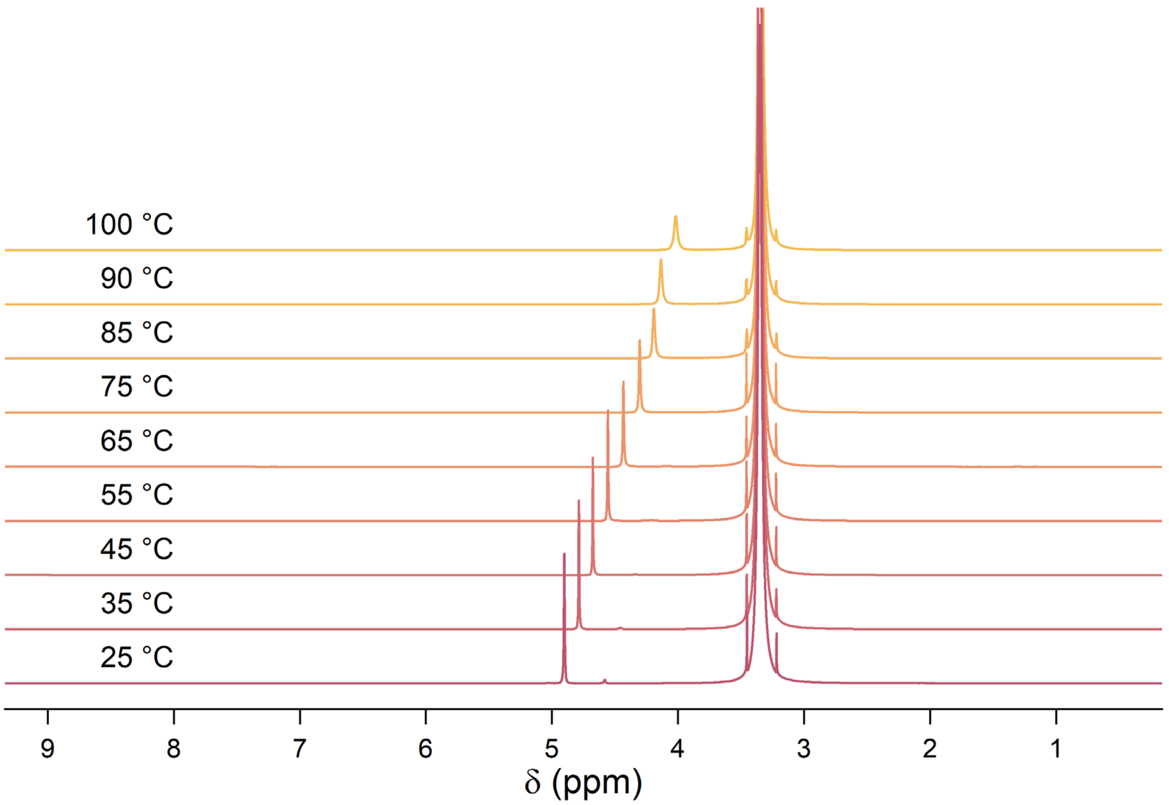

Figure S63. Background corrected ^{1}H-NMR spectra of MeOH at different temperatures.

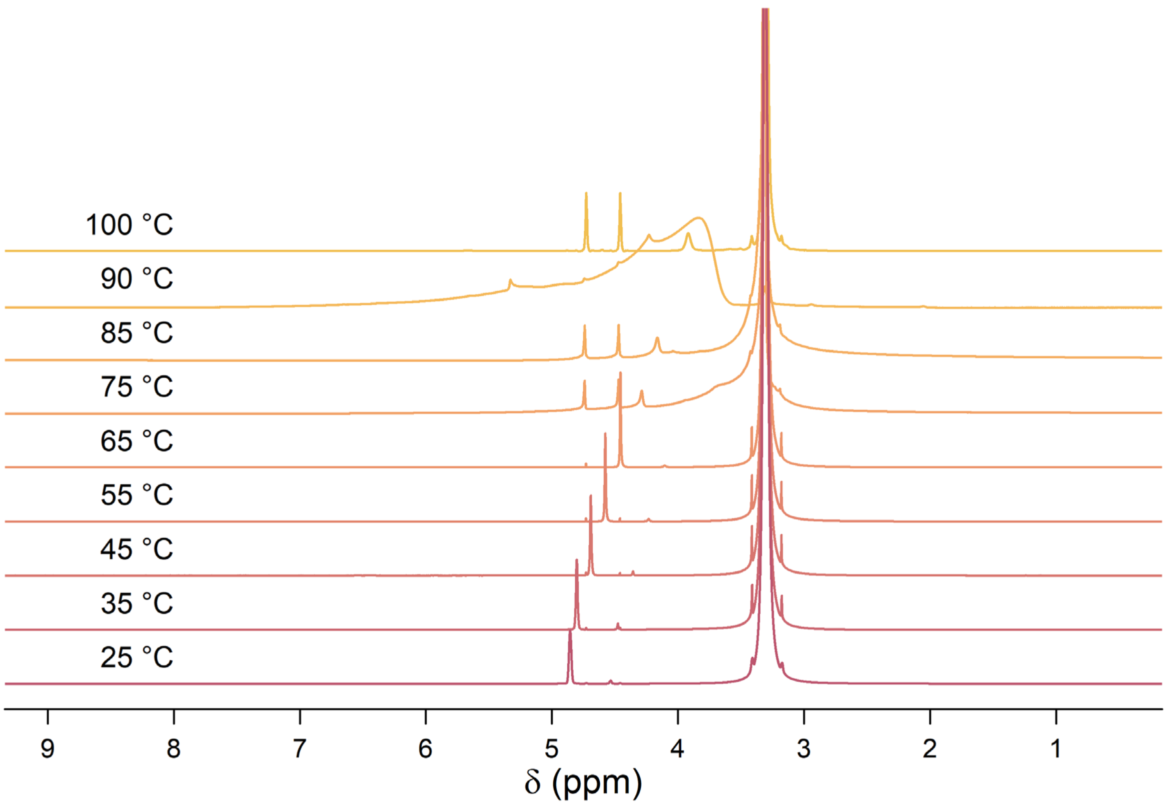

Figure S64. Background corrected ^{1}H-NMR spectra of the dissolution process of ^{13}C-**PFA** in d^{4}-MeOH at different temperatures. Spectrum at 90 °C is distorted due to bubble formation inside the NMR tube.

10. DFT calculated IR spectra

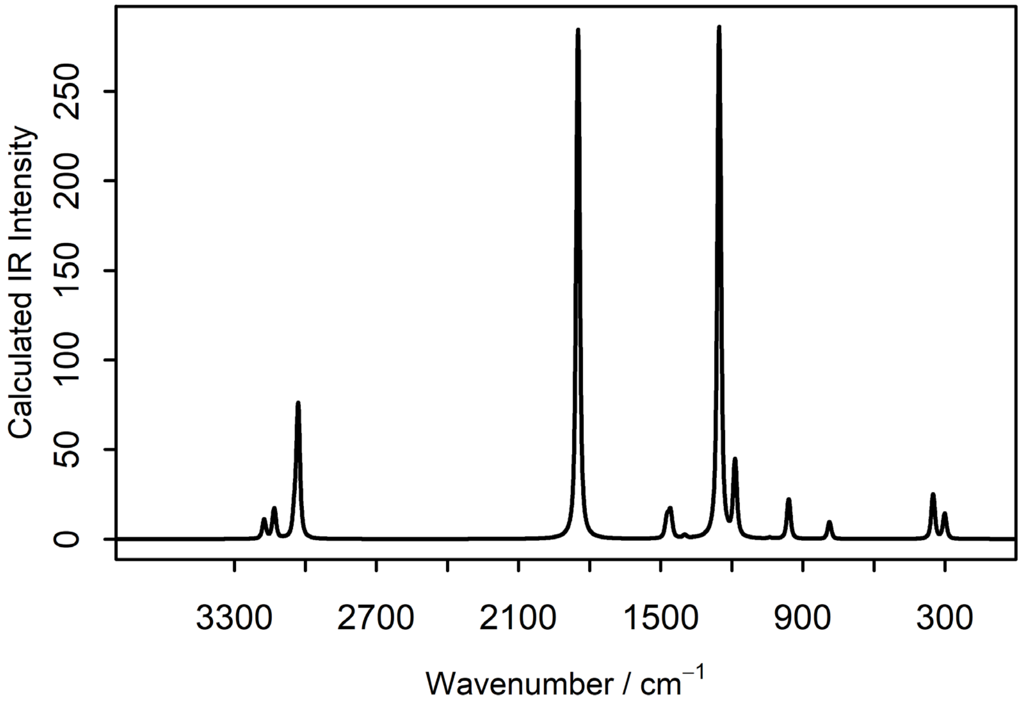

Figure S65. DFT calculated IR spectrum of HCOOMe (FWHM: 18 cm^{-1}).

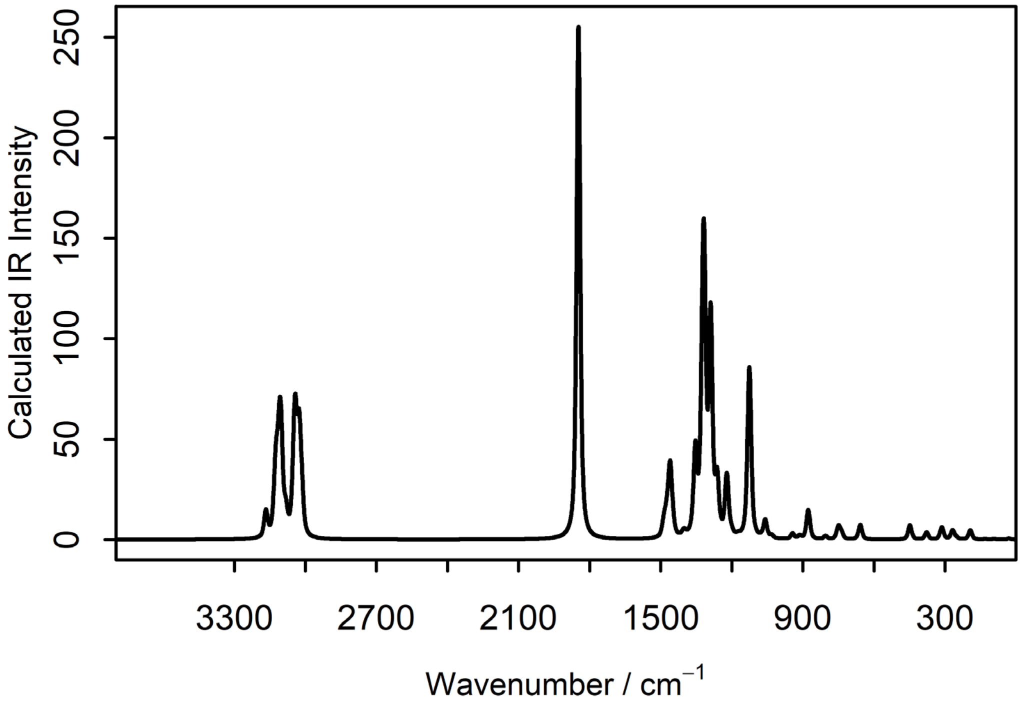

Figure S66. DFT calculated IR spectrum of C_4H_9COOMe (**MF**) (FWHM: 18 cm^{-1}).

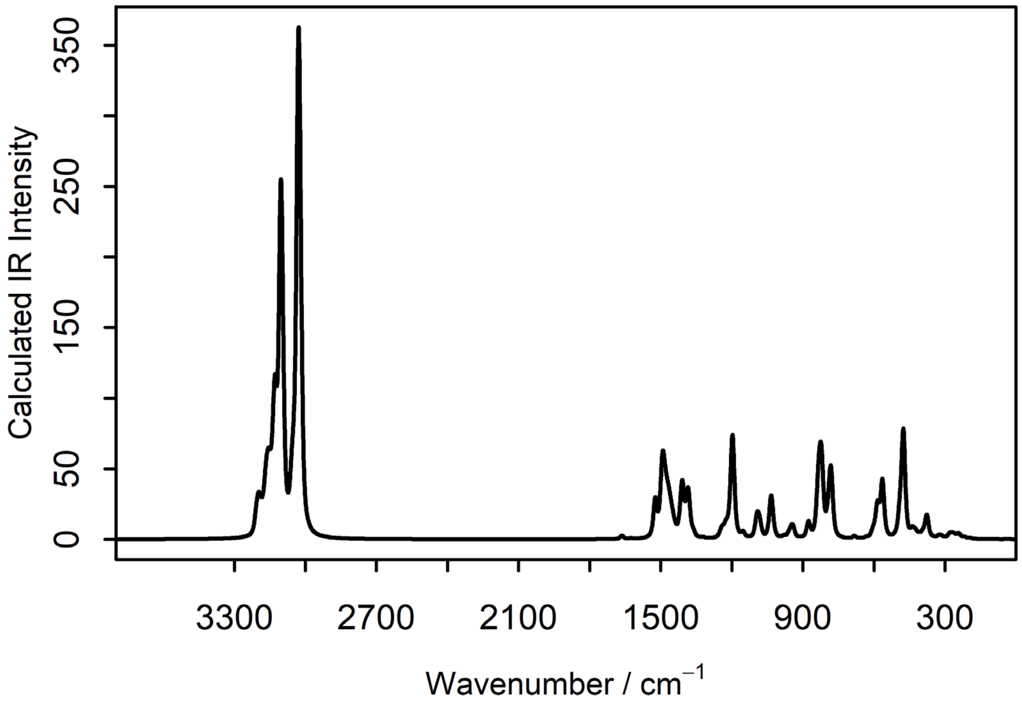

Figure S67. DFT calculated IR spectrum of [Pd(d^{t}bpx)] (**I**) (FWHM: 18 cm^{-1}).

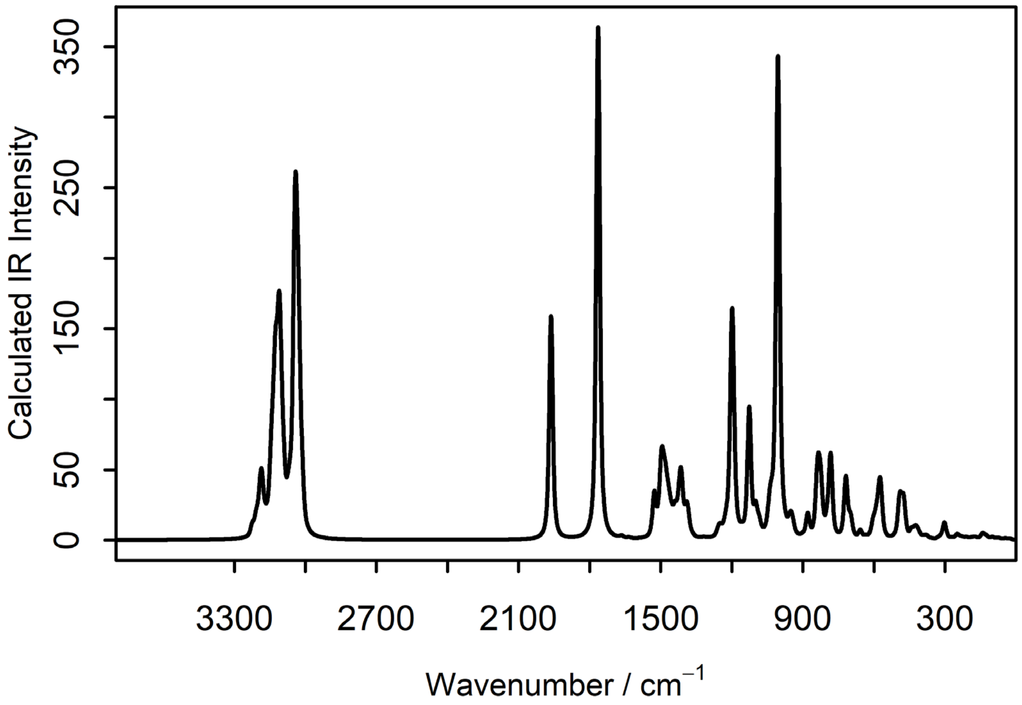

Figure S68. DFT calculated IR spectrum of [Pd(d^{t}bpx)H(COOMe)] (**II**) (FWHM: 18 cm^{-1}).

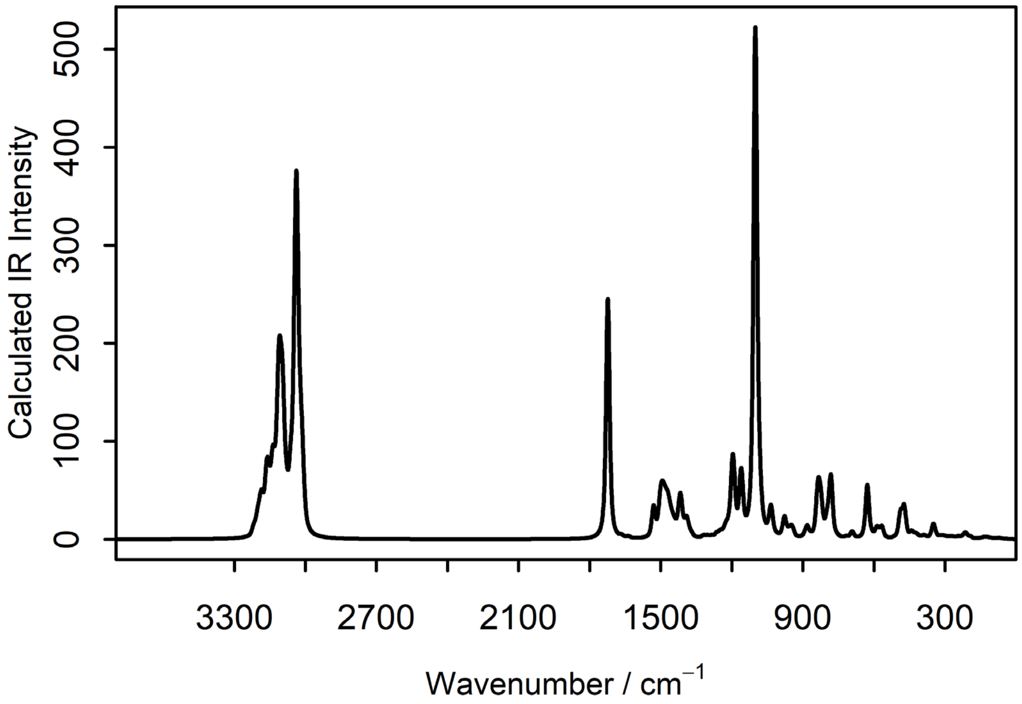

Figure S69. DFT calculated IR spectrum of [Pd(d*t*bpx)(C_4H_9)(COOMe)] (**IVa**) (FWHM: 18 cm^{-1}).

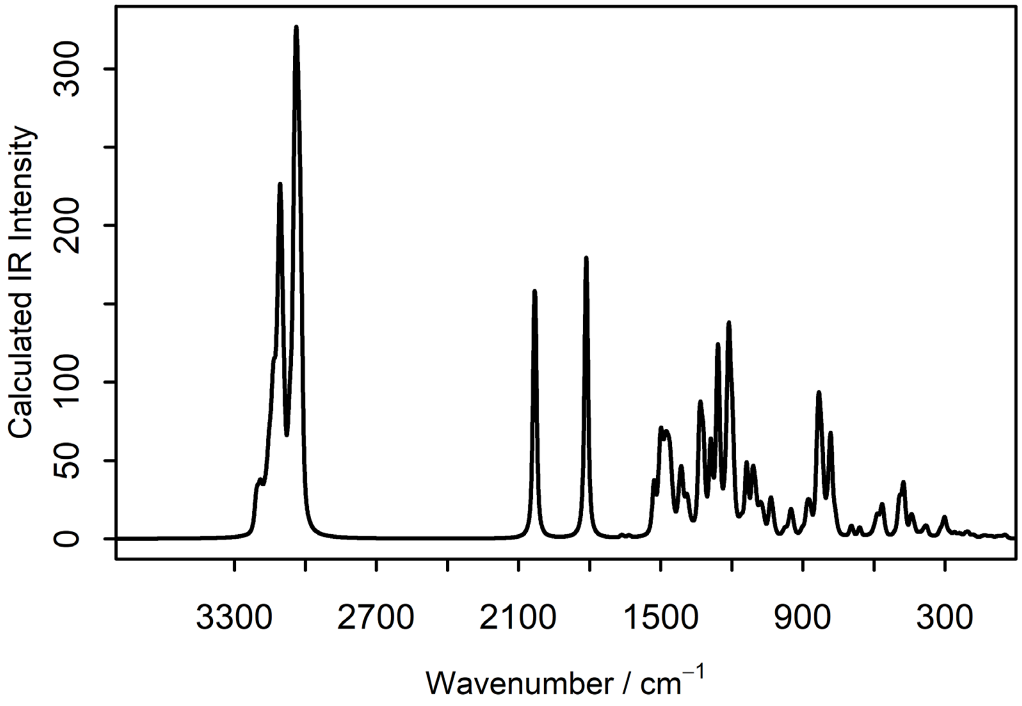

Figure S70. DFT calculated IR spectrum of [Pd(d*t*bpx)H(C_4H_8COOMe)] (**IVb**) (FWHM: 18 cm^{-1}).

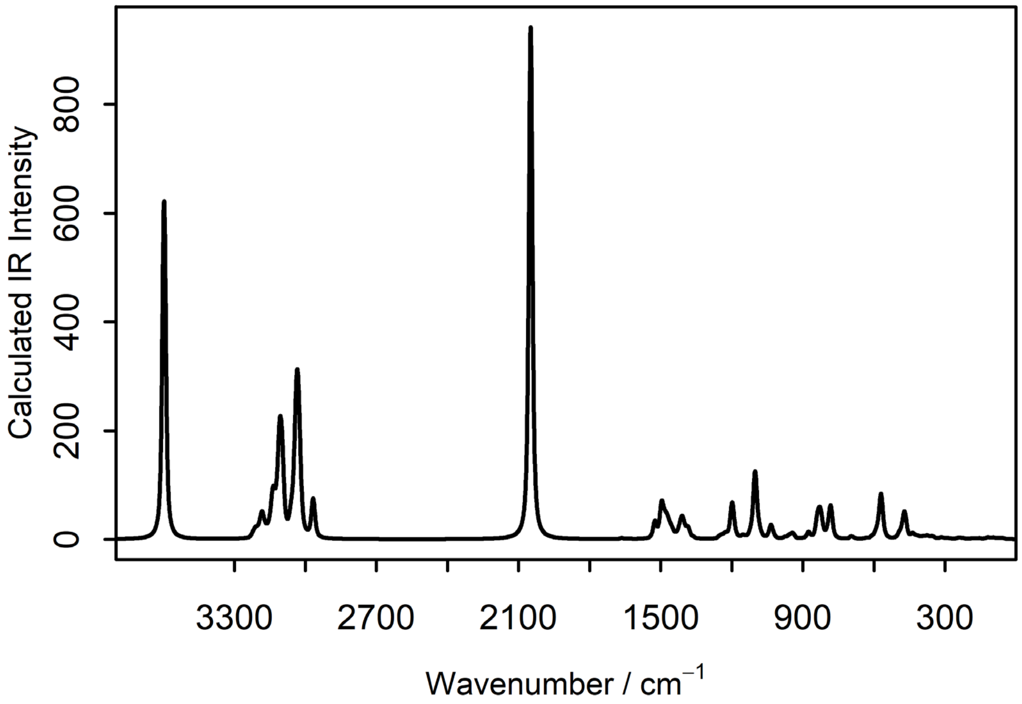

Figure S71. DFT calculated IR spectrum of [Pd(d^{t}bpx)(CO)(MeOH)] (**III-CO**) (FWHM: 18 cm^{-1}).

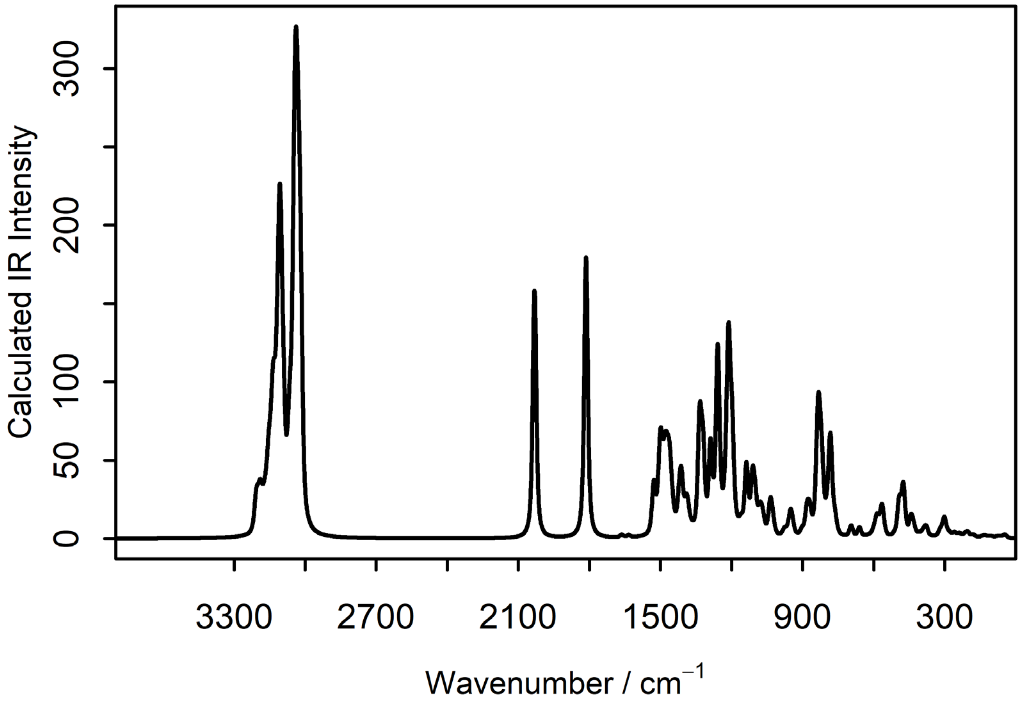

Figure S72. DFT calculated IR spectrum of [Pd(d^{t}bpx)(CO)] (**IV-CO**) (FWHM: 18 cm^{-1}).

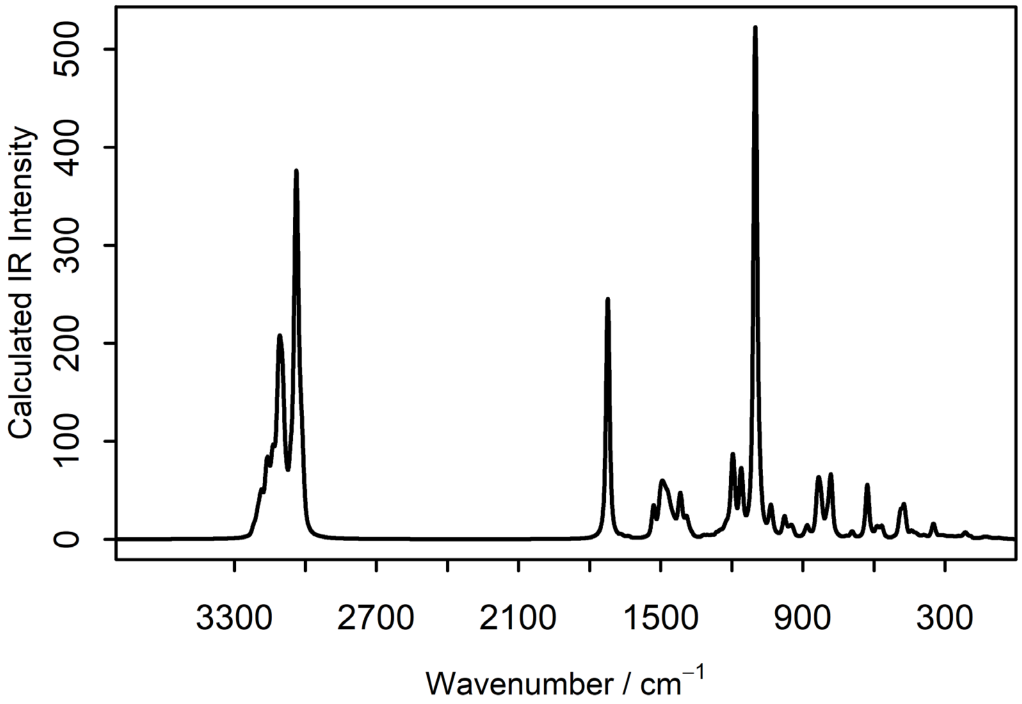

Figure S73. DFT calculated IR spectrum of [Pd(d^{t}bpx)(CO_2)(CH_4)] (**III-CO$_2$**) (FWHM: 18 cm^{-1}).

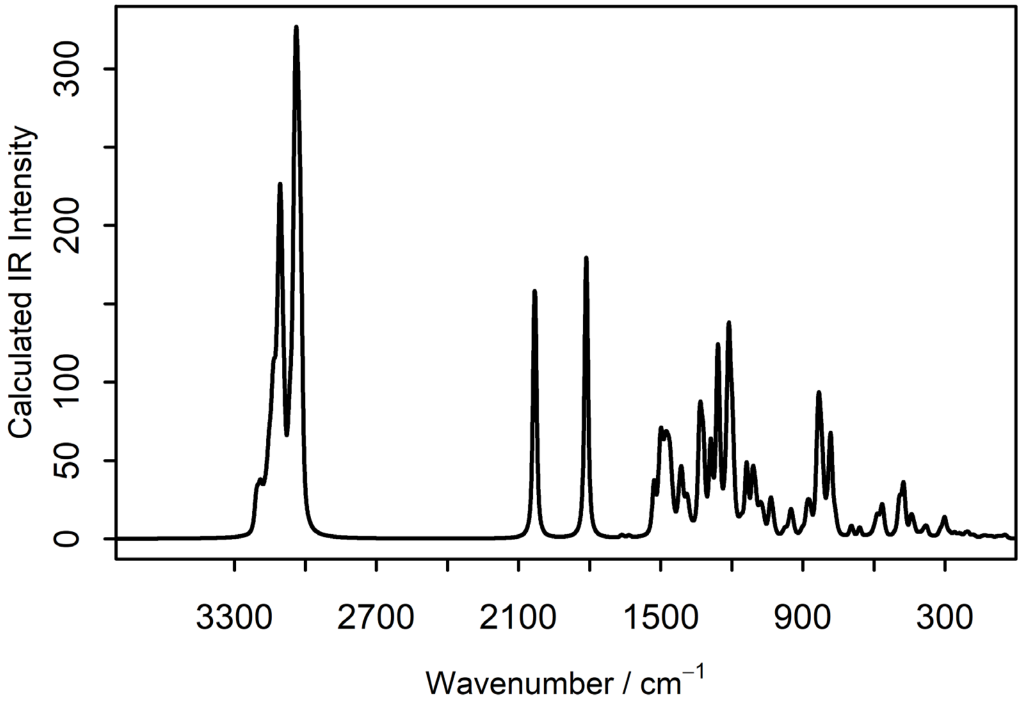

Figure S74. DFT calculated IR spectrum of [Pd(d^{t}bpx)(CO_2)] (**IV-CO$_2$**) (FWHM: 18 cm^{-1}).

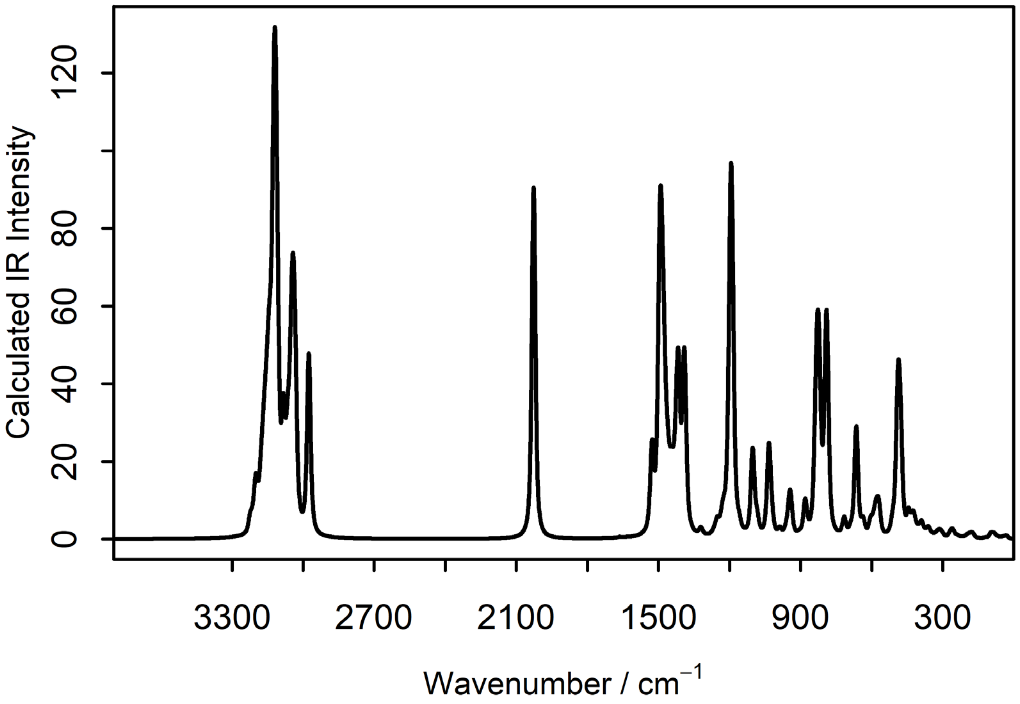

Figure S75. DFT calculated IR spectrum of [Pd(d^{t}bpx)H]$^+$ (**I-AC**) (FWHM: 18 cm^{-1}).

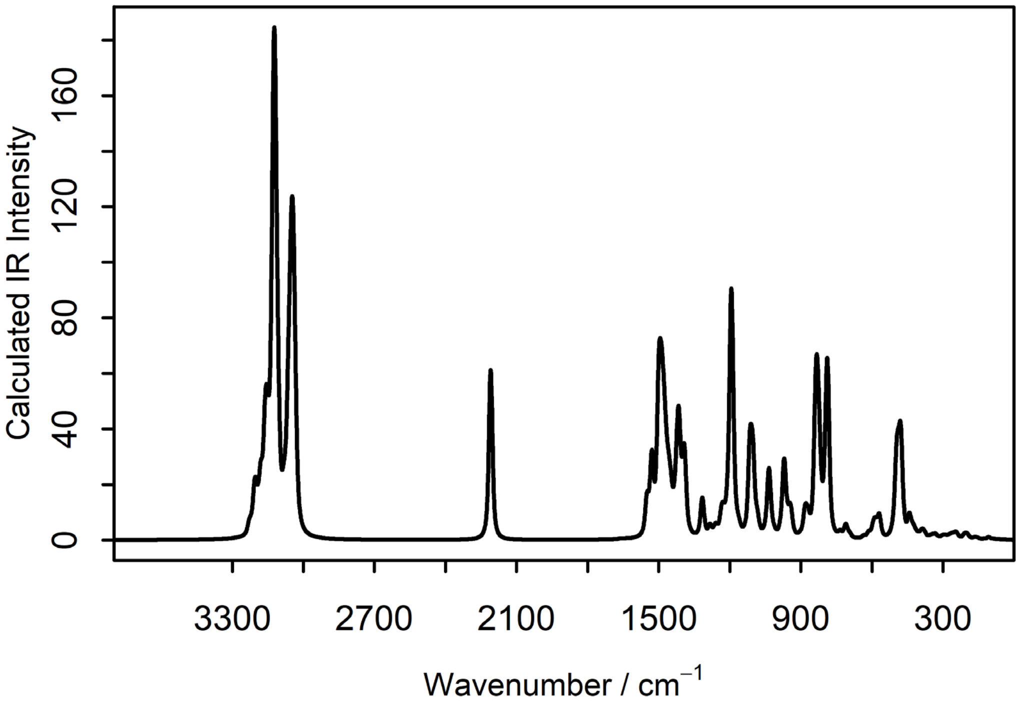

Figure S76. DFT calculated IR spectrum of [Pd(d^{t}bpx)(C_4H_9)]$^+$ (**II-AC**) (FWHM: 18 cm^{-1}).

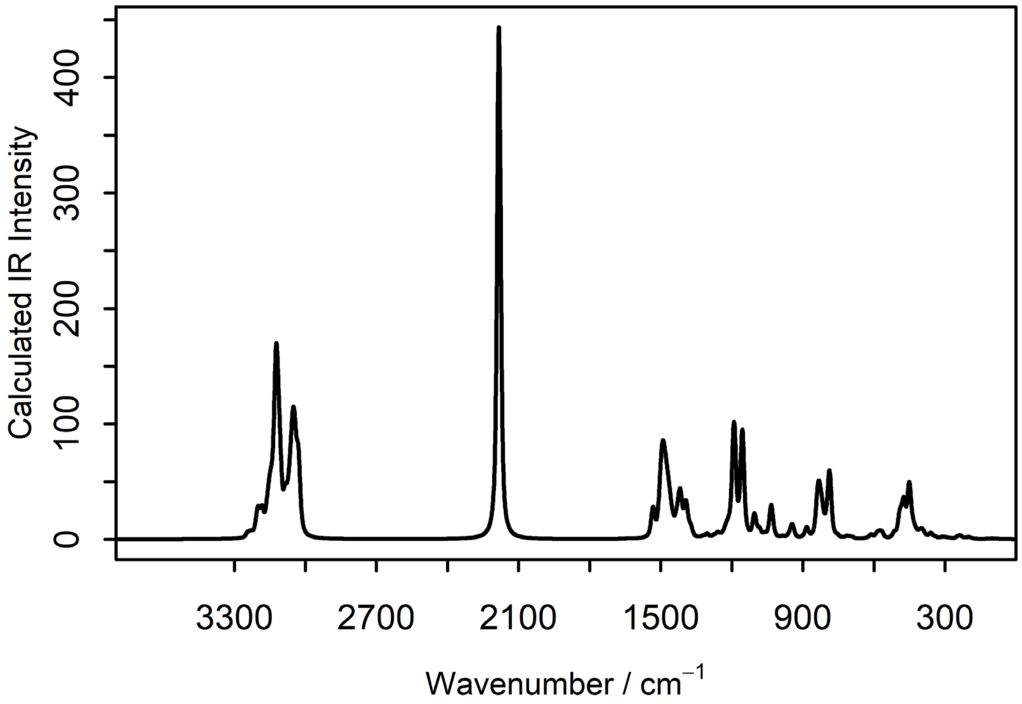

Figure S77. DFT calculated IR spectrum of [Pd(d^{t}bpx)(C_4H_9)(CO)]$^+$ (**III-AC**) (FWHM: 18 cm^{-1}).

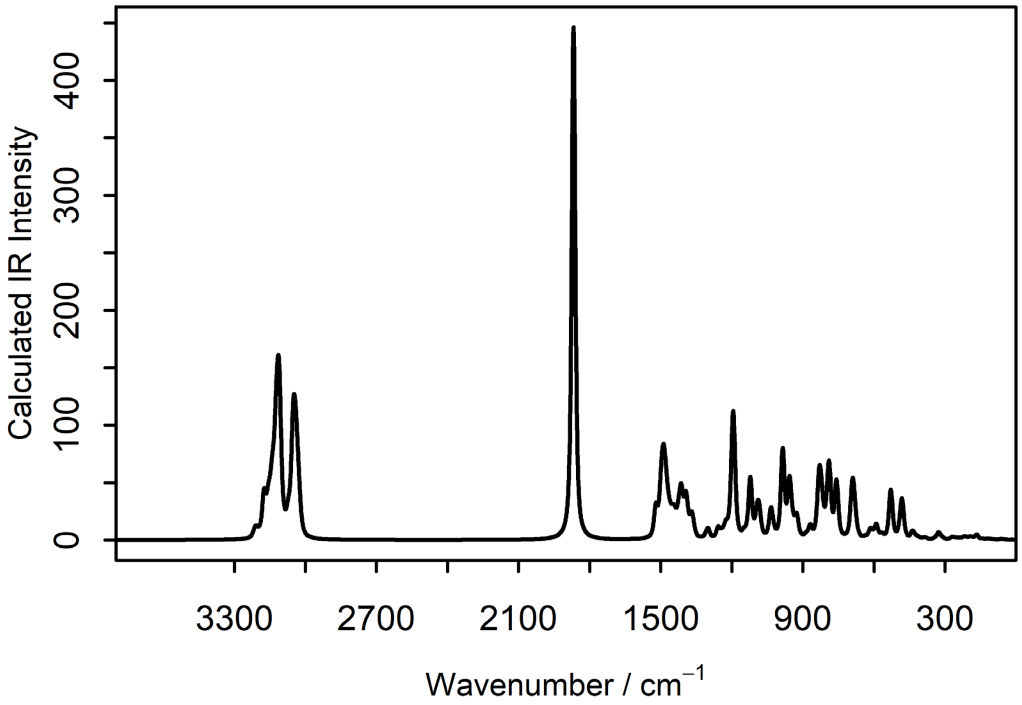

Figure S78. DFT calculated IR spectrum of [Pd(d^{t}bpx)(C(O)C_4H_9)]$^+$ (**IV-AC**) (FWHM: 18 cm^{-1}).

11. References

[1] R. Sang, P. Kucmierczyk, K. Dong, R. Franke, H. Neumann, R. Jackstell, M. Beller, *J. Am. Chem. Soc.* **2018**, *140*, 5217–5223.

[2] P. Luehring, A. Schumpe, *J. Chem. Eng. Data* **1989**, *34*, 250–252.

[3] M. Nič, J. Jirát, B. Košata, A. Jenkins, A. McNaught, *IUPAC Compendium of Chemical Terminology*, IUPAC, Research Triagle Park, NC, **2009**.

[4] E. Moser, E. Laistler, F. Schmitt, G. Kontaxis, *Front. Phys.* **2017**, *5*, 5–19.

[5] Mestrelab Research S.L., *MestReNova*, Mestrelab Research S.L., Santiago de Compostela, Spain, **2015**.

[6] G. R. Fulmer, A. J. M. Miller, N. H. Sherden, H. E. Gottlieb, A. Nudelman, B. M. Stoltz, J. E. Bercaw, K. I. Goldberg, *Organometallics* **2010**, *29*, 2176–2179.

[7] S. N. Bernstein, *Commun. Soc. Math. Kharkov* **1912**, *12*, 1–2.

[8] R Core team, *R: A Language and Environment for Statistical Computing*, R Foundation for Statistical Computing, Vienna, Austria, **2020**.

[9] Karline Soetaert, Thomas Petzoldt, R. Woodrow Setzer, *J. Stat. Softw.* **2010**, *33*, 1–25.

[10] K. Soetaert, T. Petzoldt, *J. Stat. Softw.* **2010**, *33*, 1–28.

[11] E. Neuwirth, *RColorBrewer: ColorBrewer Palettes*, **2014**.

[12] R. Ihaka, P. Murrell, K. Hornik, J. C. Fisher, A. Zeileis, *colorspace: Color*, **2016**.

[13] R. Geitner, B. M. Weckhuysen, *Chem. Eur. J.* **2020**, *26*, 5297–5302.

[14] M. J. Frisch, G. W. Trucks, H. B. Schlegel, G. E. Scuseria, M. A. Robb, J. R. Cheeseman, G. Scalmani, V. Barone, G. A. Petersson, H. Nakatsuji et al., *Gaussian16 (Revision B.01)*, Gaussian, Inc, Wallingford, CT, USA, **2016**.

[15] A. D. Becke, *J. Chem. Phys.* **1993**, *98*, 5648–5652.

[16] F. Weigend, R. Ahlrichs, *Phys. Chem. Chem. Phys.* **2005**, *7*, 3297–3305.

[17] S. Grimme, S. Ehrlich, L. Goerigk, *J. Comput. Chem.* **2011**, *32*, 1456–1465.

[18] G. Henkelman, B. P. Uberuaga, H. Jónsson, *J. Chem. Phys.* **2000**, *113*, 9901–9904.

[19] J. Steinmetzer, S. Kupfer, S. Gräfe, *Int. J. Quantum Chem.* **2020**, e26390.

[20] a) S. Grimme, C. Bannwarth, P. Shushkov, *J. Chem. Theory Comput.* **2017**, *13*, 1989–2009; b) C. Bannwarth, S. Ehlert, S. Grimme, *J. Chem. Theory Comput.* **2019**, *15*, 1652–1671.

[21] H. B. Schlegel, *J. Comput. Chem.* **1982**, *3*, 214–218.

[22] J.J. Olivero, R.L. Longbothum, *J. Quant. Spectrosc. Radiat. Transfer* **1977**, *17*, 233–236.

[23] M. Maiwald, H. H. Fischer, M. Ott, R. Peschla, C. Kuhnert, C. G. Kreiter, G. Maurer, H. Hasse, *Ind. Eng. Chem. Res.* **2003**, *42*, 259–266.

[24] A. Peter, S. M. Fehr, V. Dybbert, D. Himmel, I. Lindner, E. Jacob, M. Ouda, A. Schaadt, R. J. White, H. Scherer et al., *Angew. Chem. Int. Ed.* **2018**, *57*, 9461–9464.

[25] K. Mistry, P. G. Pringle, H. A. Sparkes, D. F. Wass, *Organometallics* **2020**, *39*, 468–477.

[26] R. M. Bellabarba, R. P. Tooze, A. M. Z. Slawin, *Chem. Commun.* **2003**, 1916–1917.

[27] W. Clegg, G. R. Eastham, M. R. J. Elsegood, B. T. Heaton, J. A. Iggo, R. P. Tooze, R. Whyman, S. Zacchini, *J. Chem. Soc., Dalton Trans.* **2002**, 3300–3308.

[28] a) Y. Pan, J. T. Mague, M. J. Fink, *Organometallics* **1992**, *11*, 3495–3497; b) M. D. Fryzuk, B. R. Lloyd, G. K. B. Clentsmith, S. J. Rettig, *J. Am. Chem. Soc.* **1994**, *116*, 3804–3812.

[29] J. Liu, B. T. Heaton, J. A. Iggo, R. Whyman, *Chem. Commun.* **2004**, 1326–1327.

[30] a) Y. Zhao, L. Jin, P. Li, A. Lei, *J. Am. Chem. Soc.* **2008**, *130*, 9429–9433; b) I. Tóth, C. J. Elsevier, *J. Chem. Soc., Chem. Commun.* **1993**, 529–531.

[31] J. A. Buonomo, C. G. Eiden, C. C. Aldrich, *Chem. Eur. J.* **2017**, *23*, 14434–14438.

[32] F. Eisenträger, A. Göthlich, I. Gruber, H. Heiss, C. A. Kiener, C. Krüger, J. Ulrich Notheis, F. Rominger, G. Scherhag, M. Schultz et al., *New J. Chem.* **2003**, *27*, 540–550.

[33] W.-C. Shih, O. V. Ozerov, *Organometallics* **2015**, *34*, 4591–4597.

[34] F. Dornhaus, S. Scholz, I. Sänger, M. Bolte, M. Wagner, H.-W. Lerner, *Z. Anorg. Allg. Chem.* **2009**, *635*, 2263–2272.

[35] K. B. Renkema, M. Ogasawara, W. E. Streib, J. C. Huffman, K. G. Caulton, *Inorg. Chim. Acta* **1999**, *291*, 226–230.

[36] R. Dyapa, L. T. Dockery, M. A. Walczak, *Organic & biomolecular chemistry* **2016**, *15*, 51–55.

[37]W. Clegg, G. R. Eastham, M. R. J. Elsegood, B. T. Heaton, J. A. Iggo, R. P. Tooze, R. Whyman, S. Zacchini, *Organometallics* **2002**, *21*, 1832–1840.
[38]G. R. Eastham, *PhD Thesis*, University of Durham, Durham, **1998**.
